# Osteopathy for Musculoskeletal Pain: A Systematic and Umbrella Review of Effectiveness and Safety

**DOI:** 10.3390/healthcare14070928

**Published:** 2026-04-02

**Authors:** Lucia Gassner, Viktoria Hofer, Ingrid Zechmeister-Koss, Inanna Reinsperger

**Affiliations:** HTA Austria—Austrian Institute for Health Technology Assessment GmbH, Josefstädter Straße 39, 1080 Vienna, Austria; viktoria.hofer@aihta.at (V.H.); ingrid.zechmeister-koss@aihta.at (I.Z.-K.);

**Keywords:** osteopathy, musculoskeletal pain, systematic review

## Abstract

**Background:** Musculoskeletal pain affects an estimated 1.7 billion people worldwide and ranks among the leading causes of global disability. This review evaluates the effectiveness and safety of osteopathy in treating musculoskeletal pain across multiple body regions and conditions. **Methods:** A systematic literature review following PRISMA guidelines was conducted across five databases (Embase, Medline via Ovid, The Cochrane Library, PEDro, and INAHTA), yielding 964 citations. Eligible studies were RCTs published in English or German up to May 2022; conference abstracts were excluded. A hybrid design was employed: a systematic review of RCTs for neck, shoulder, knee, foot, osteoporosis, and fibromyalgia was combined with a pre-specified umbrella review component for chronic non-specific low back pain (registered in PROSPERO) to avoid duplication of an existing high-confidence evidence synthesis. From 35 critically appraised articles, the best available evidence (*n* = 15) was selected per body region based on a risk of bias (RoB) assessment (Cochrane Collaboration tool, version 1); the existing review was appraised with AMSTAR 2. An updated search (2022–July 2025) was performed without a RoB assessment. Data were synthesised qualitatively and reported narratively. **Results:** Fifteen RCTs and one systematic review were included, covering eight body regions and conditions (2408 participants). Pain improved immediately post-treatment in most regions; statistically significant between-group differences were less consistent at mid- and long-term follow-ups. Key findings: neck pain (*n* = four RCTs)—improvement in three of four studies immediately post-treatment; shoulder pain (*n* = two RCTs)—improvements across all follow-up points in one study; low back pain (*n* = one systematic review, 10 RCTs, 1160 participants)—pain reduced immediately and at mid-term follow-up; knee pain (*n* = two RCTs)—significant reduction in one study; foot pain (*n* = two RCTs)—improvement in both studies post-treatment and at mid-term follow-up; osteoporosis (*n* = one RCT)—no improvement immediately post-treatment; fibromyalgia (*n* = two RCTs)—significant between-group differences in one study post-treatment and at mid-term follow-up. Functional outcomes were heterogeneous across regions. Adverse events were minor and transient; no serious side effects were reported across any included study. The updated search (2022–July 2025) identified 12 additional RCTs across five regions, with findings broadly consistent with the primary analysis, though results for the neck region were marginally less favourable. **Discussion:** Based on current evidence, osteopathy can improve neck and low back pain for up to three months and may reduce shoulder and foot pain; evidence for other body regions remains inconclusive. RoB was unclear to high across studies, largely due to the inherent inability to blind patients and practitioners in manual therapy trials. Substantial heterogeneity in interventions, outcome measures, and study designs limits comparability. Overall certainty of evidence was low to moderate, warranting cautious interpretation. The consistent absence of serious adverse events across all included studies supports osteopathy as a safe therapeutic option. High-quality research with standardised interventions, rigorous designs, long-term follow-ups, and a focus on technique, dosage, and safety is needed to inform clinical practice and healthcare policy. This research did not receive any specific grant from funding agencies in the public, commercial, or not-for-profit sector.

## 1. Introduction

Musculoskeletal pain-related conditions are among the leading contributors to global disability, estimated to affect 1.7 billion people worldwide according to the Global Burden of Disease study 2019 [1]. This health burden accounts for approximately a quarter of general practice consultations in countries such as the United Kingdom (UK), and frequently impairs quality of life (QoL), functioning, and well-being [2]. Pain can adversely affect patients’ social and psychological well-being, particularly in those experiencing high levels of stress or struggling with self-management. A high rate of comorbidity between pain and mental health conditions is well established [3]. Musculoskeletal pain may arise in various body regions, from the neck to the foot [2], and is managed in primary care by a range of healthcare professionals, including medical doctors, physiotherapists, chiropractors, and osteopaths [4].

Osteopaths treat a variety of health conditions, with musculoskeletal pain—most commonly low back pain—representing the primary indication. Rheumatic conditions are another frequent indication. In the UK, osteopathy is included in national guidelines for the management of chronic low back pain [5,6]. The Osteopathic International Alliance (OIA) confirmed in its 2020 report that osteopaths predominantly treat patients with musculoskeletal conditions and pain [7]. As a health discipline, osteopathy emphasises the role of the musculoskeletal system in health [8].

According to the World Health Organization (WHO), osteopathy is a patient-centred primary healthcare discipline that relies on manual contact for diagnosis and treatment, grounded in the interrelationship between the body’s structure and function and the integration of body, mind, and spirit in health and disease [3,9,10,11]. The osteopathic philosophy is consistent with the biopsychosocial model, acknowledging that psychological factors may influence homeostasis and physiology and adopting a whole-person approach to illness. Treatment technique, frequency, and duration are tailored to the individual patient’s needs [3]. Osteopathy currently lacks consistent standards in education, training, and practice [12], and considerable heterogeneity exists in its regulation and recognition across countries [9]. These inconsistencies have direct implications for healthcare policy: they complicate the integration of osteopathy into national health systems and the development of evidence-based clinical guidelines, underscoring the need for a rigorous, policy-relevant evidence synthesis.

While previous systematic reviews have addressed osteopathic treatment for low back pain [13,14] and musculoskeletal pain more broadly [2,4], prior reviews either included only a limited number of randomised controlled trials (RCTs) without stratification by body region [2], focused primarily on comparative effectiveness and health economics [6], or were restricted to a single technique or anatomical site—limiting their utility for practitioners and health technology assessors. The evidence for other musculoskeletal regions remains sparse, methodologically heterogeneous, and insufficiently synthesised to support clinical or policy decision-making. Furthermore, no recent review has systematically evaluated both effectiveness and safety across multiple musculoskeletal regions simultaneously, nor has any review been conducted within an explicit health technology assessment (HTA) framework that prioritises internal validity for policy recommendations. A comprehensive, methodologically rigorous synthesis is therefore needed to inform clinical guidelines, reimbursement decisions, and the integration of osteopathy into primary healthcare systems—areas of increasing relevance given the growing recognition of osteopathy in national health policies across Europe and beyond [7,11].

To address this gap, we conducted a hybrid systematic and umbrella review evaluating the effectiveness and safety of osteopathic interventions across multiple musculoskeletal body regions and conditions. A de novo systematic search of RCTs was performed for most regions; chronic non-specific low back pain was addressed via a pre-specified umbrella review component, given the availability of a recent high-confidence synthesis [13]. The review was prospectively registered on PROSPERO (CRD42022330778) and conducted in accordance with PRISMA guidelines [15].

## 2. Methods

### 2.1. Study Design and Scope

This systematic review with umbrella review component was conducted in accordance with PRISMA guidelines [15] and registered prospectively on PROSPERO (CRD42022330778). The primary component comprised a systematic search and synthesis of RCTs addressing osteopathic treatment for musculoskeletal pain across multiple body regions and conditions (neck, shoulder, knee, foot, osteoporosis, fibromyalgia, and mixed neck/low back pain). Chronic non-specific low back pain was addressed through a prespecified umbrella review component rather than a de novo RCT search, based on the recent publication of a high-quality systematic review and meta-analysis on this topic [13]. Conducting a parallel search would have duplicated an existing high-confidence synthesis without adding methodological value [16]. The pre-specified review [13] was appraised using A Measurement Tool to Assess Systematic Reviews Version 2 (AMSTAR 2), and its findings are reported alongside the primary RCT results. This hybrid design—combining a de novo RCT search with a prespecified umbrella review component—means the evidence base for low back pain differs in its assembly from that of other body regions, which is an acknowledged limitation.

### 2.2. Search Strategy

A systematic literature search was conducted on 18–19 May 2022 across five databases: Embase (*n* = 621), Medline via Ovid (*n* = 505), The Cochrane Library (*n* = 458), PEDro (*n* = 69), and INAHTA (*n* = 9). Medical Subject Headings (MeSH) terms included: chronic pain, flank pain, metatarsalgia, musculoskeletal pain, neck pain, nociceptive pain, intractable pain, postoperative pain, procedural pain, referred pain, pelvic girdle pain, and piriformis muscle syndrome. Full search strategies are provided in the Supplementary Materials. The search was restricted to English and German publications from database inception to May 2022; conference abstracts were excluded.

An updated search covering May 2022 to July 2025 identified 486 additional articles, of which 12 met the inclusion criteria. The same eligibility criteria were applied; however, no formal risk of bias (RoB) assessment was conducted for updated search studies due to resource constraints. This constitutes a deviation from PRISMA standards and from the methodology applied in the primary analysis. Consequently, updated search findings are reported separately and treated as exploratory and hypothesis-generating only, not as additions to the primary evidence base. The search strategies are available in the Supplementary Materials.

### 2.3. Eligibility Criteria

Inclusion and exclusion criteria were defined a priori according to the Population, Intervention, Comparator, Outcome, Study design (PICOS) framework (Table 1).

Population: Adults (≥18 years) with musculoskeletal pain conditions, encompassing both regionally localised disorders and systemic conditions affecting the musculoskeletal system. Chronic non-specific low back pain was excluded from the primary RCT search (addressed via the umbrella review component).

Intervention: Any osteopathic technique—delivered alone or adjunctively, and by osteopaths or other qualified therapists—within an osteopathic treatment framework, including craniosacral therapy, osteopathic manipulative treatment (OMT), myofascial release, strain–counterstrain, muscle energy technique, high-velocity low-amplitude manipulation, soft tissue technique, and pressure release technique. Dry needling was eligible if used as an adjunct to a broader osteopathic intervention, but not as a sole or primary intervention. Chiropractic as a distinct profession, proprioceptive neuromuscular facilitation, electrotherapy, and self-applied techniques were excluded. Spinal manipulative treatment and lumbopelvic manipulation were eligible when delivered within an osteopathic framework. Several included techniques (e.g., myofascial release, craniosacral therapy) are also applied outside osteopathic practice; their inclusion reflects the WHO definition of osteopathy as a manual contact-based discipline encompassing a range of techniques, consistent with the heterogeneous nature of osteopathic practice internationally. Observed effects should therefore be interpreted as effects of osteopathic techniques delivered within an osteopathic treatment framework, rather than as effects of osteopathy as a uniquely differentiated discipline.

Comparator: Any control condition (no treatment, waiting list, sham/placebo, physiotherapy, standard care, or pharmacological treatment). Studies using surgical comparators were excluded.

Outcomes: The primary outcome was pain assessed by any validated instrument (e.g., visual analogue scale (VAS), numerical rating scale (NRS), McGill Pain Questionnaire). Secondary outcomes included functional status, range of motion (ROM), QoL, mental and physical health, fatigue, depression, anxiety, body awareness, and symptom improvement, as well as adverse events and side effects.

Study design: RCTs only. At abstract screening, studies with fewer than 25 randomised participants were excluded; at full-text assessment, the threshold was raised to 50 participants, in line with evidence that smaller trials are associated with substantially inflated treatment effect estimates [21].

### 2.4. Study Selection

**Figure 1:**
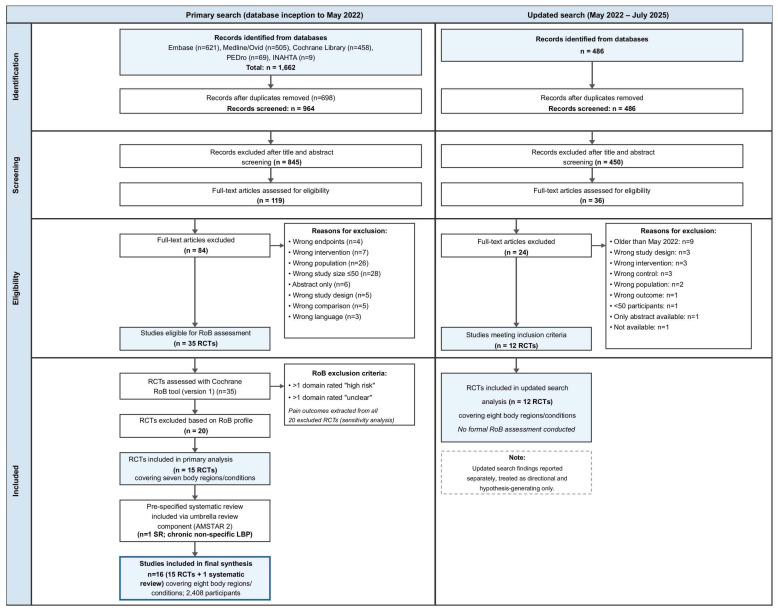
Flow chart of study selection (PRISMA flow diagram). Abbreviations: AMSTAR 2—A Measurement Tool to Assess Systematic Reviews (Version 2); Embase—Excerpta Medica Database; INAHTA—International Network of Agencies for Health Technology Assessment; LBP—low back pain; Medline/Ovid—Medical Literature Analysis and Retrieval System Online; n—number; PEDro—Physiotherapy Evidence Database; PRISMA—Preferred Reporting Items for Systematic Reviews and Meta-Analyses; RCT—randomised controlled trial; RoB—risk of bias; SR—systematic review.

After deduplication, 964 citations were identified from the primary search. Following title and abstract screening, 845 records were excluded; 119 full texts were independently assessed by two researchers (LG, VH), yielding 35 RCTs eligible for RoB assessment. Of these, 20 were subsequently excluded based on their RoB profile [22,23,24,25,26,27,28,29,30,31,32,33,34,35,36,37,38,39,40,41]; these studies and their primary reason for exclusion are listed in Supplementary Materials, Table S2. Disagreements were resolved by discussion or, where necessary, by a third researcher (IR). The full selection process is shown in Figure 1. For the updated search, 486 articles were identified, 36 full texts were assessed, and 12 met the inclusion criteria.

### 2.5. Risk of Bias Assessment and Study Selection for Primary Analysis

All 35 eligible RCTs were assessed using the Cochrane Collaboration’s RoB tool (version 1), consistent with HTA methodology, which regards the Cochrane RoB tool as the gold standard for assessing internal validity of RCTs to support healthcare decision-making [42,43] (see Tables S1 and S2 from the Supplementary Materials). To ensure inter-rater reliability, the first three studies were assessed independently and blinded prior to joint calibration; subsequent studies were divided between the two researchers, and all high-risk ratings were discussed before final exclusion.

As stated in PROSPERO, we initially planned to exclude all RCTs with high RoB. However, strict application of this criterion would not have yielded coverage across all seven target body regions and conditions. Following completion of the RoB assessments and prior to data extraction, the inclusion strategy was revised in a two-step procedure to ensure complete regional coverage. This revision constitutes a deviation from the strategy pre-registered on PROSPERO and is acknowledged as a limitation. In a first step, for each body region or condition, up to two studies with the most favourable RoB profile were selected to ensure coverage of all seven regions and conditions. This step yielded 13 RCTs. In a second step, all studies meeting a minimum threshold—defined as a maximum of one domain rated ‘high risk’ and one domain rated ‘unclear’—were retained. This threshold was defined post hoc by the review authors as a pragmatic minimum standard for inclusion; while no specific threshold is prescribed in HTA guidance, this approach reflects the principle of differentiating between studies based on their RoB profile, in line with EUnetHTA guidance on internal validity [42]. Application of this second step identified two additional studies on the neck region, bringing the total to 15 RCTs for the primary analysis.

As an exception for fibromyalgia, two studies with identical RoB profiles [22,44] were both eligible; [44] was selected to enable comparison of two distinct osteopathic techniques (craniosacral therapy and myofascial release). All deviations from the registered protocol are transparently reported in PROSPERO. To assess potential reporting bias, the primary pain outcome was additionally extracted from all 20 excluded RCTs and compared narratively with the included results, serving as a narrative sensitivity analysis.

The prespecified systematic review on chronic non-specific low back pain [13] was appraised separately using AMSTAR-2 [45] (Supplementary Materials, Table S3) and added to the evidence base via the umbrella review component. In total, 16 studies were included in the final synthesis: 15 RCTs from the primary search covering seven body regions and conditions, and one systematic review and meta-analysis addressing chronic non-specific low back pain via the umbrella review component.

### 2.6. Data Extraction

Data were extracted into standardised tables (Supplementary Materials, Tables S4–S21) using single extraction with independent verification (LG extracted; VH verified). Data were clustered by body region or condition. For updated search studies, one researcher (LG) extracted and verified the primary outcome (pain) from the 12 included studies; (serious) adverse events and safety outcomes were synthesised narratively, descriptively summarising the nature, frequency, and severity of reported harms.

### 2.7. Data Synthesis

A qualitative narrative synthesis was performed, with results reported by body region, including means, confidence intervals, and *p*-values as reported in individual studies. Follow-up (FU) time points were categorised as: immediate (0–7 days post-treatment), short-term (1 month), mid-term (3–6 months), and long-term (1 year).

A meta-analysis was not conducted owing to substantial clinical and methodological heterogeneity across studies, including variation in populations, interventions (techniques, session number, duration, and practitioner background), comparators, outcome instruments, and measurement time points. Furthermore, the small number of studies per body region (one to four RCTs) and the absence of any trial at low overall RoB precluded meaningful pooling. Pooling under these conditions would risk producing a statistically precise but clinically misleading estimate [46]. Reporting bias was assessed narratively as a substitute for funnel plot analysis. As recommended by the Cochrane Handbook, tests for funnel plot asymmetry require at least ten studies per meta-analysis to have sufficient power [46]; given that studies per body region ranged from one to four RCTs, funnel plots were not feasible. Instead, the primary pain outcome was extracted from all 20 excluded RCTs and compared narratively with the included results to assess whether methodological quality-based selection introduced bias. Standardised mean differences were not calculated retrospectively, as the diversity of outcome instruments and the small number of studies per region would not meaningfully improve cross-study comparability.

A formal GRADE assessment was not performed. Instead, evidence quality was evaluated using the Cochrane RoB tool and AMSTAR 2, consistent with established HTA practice [42]. As noted in the EUnetHTA methodology guidelines, the scope of GRADE extends beyond the assessment of internal validity alone, whereas the Cochrane RoB tool focuses specifically on this domain [42]. Given that no included RCT achieved a low overall risk of bias and that substantial clinical and methodological heterogeneity precluded meta-analysis, a formal GRADE rating would have been of limited interpretability in the absence of pooled effect estimates.

## 3. Results

The primary search yielded 15 RCTs and one prespecified systematic review and meta-analysis on chronic non-specific low back pain [13], covering eight body regions and conditions and involving a total of 2408 participants.

### 3.1. Study Characteristics

Detailed study characteristics are provided in Table 2 and Table 3 and in the Supplementary Materials (Tables S4–S12). The number of randomised participants per RCT ranged from 54 [47,48] to 201 [49]; participant age ranged from 16 [49] to 77 [50] years. Dropout rates ranged from zero [47,50,51,52,53] to 20 participants [54]. The prespecified systematic review included 1160 patients (mean age 43 years), with dropout rates ranging from 0 to 77% [13]. Studies originated predominantly from Spain [44,47,52,54] and Italy [13,50,55]. The number of osteopathic sessions ranged from one [51,52,55] to 50 [54], delivered mainly by osteopaths [50,55,56,57] or physiotherapists with advanced osteopathic qualifications [44,48,58]. Osteopathic techniques were applied as a sole intervention in 12 studies; three studies combined techniques [50,57,59]. The most frequently used technique was myofascial release; control conditions consisted predominantly of sham treatment [44,48,50,51,55,58,59].

### 3.2. Effectiveness Outcomes

**Figure 2:**
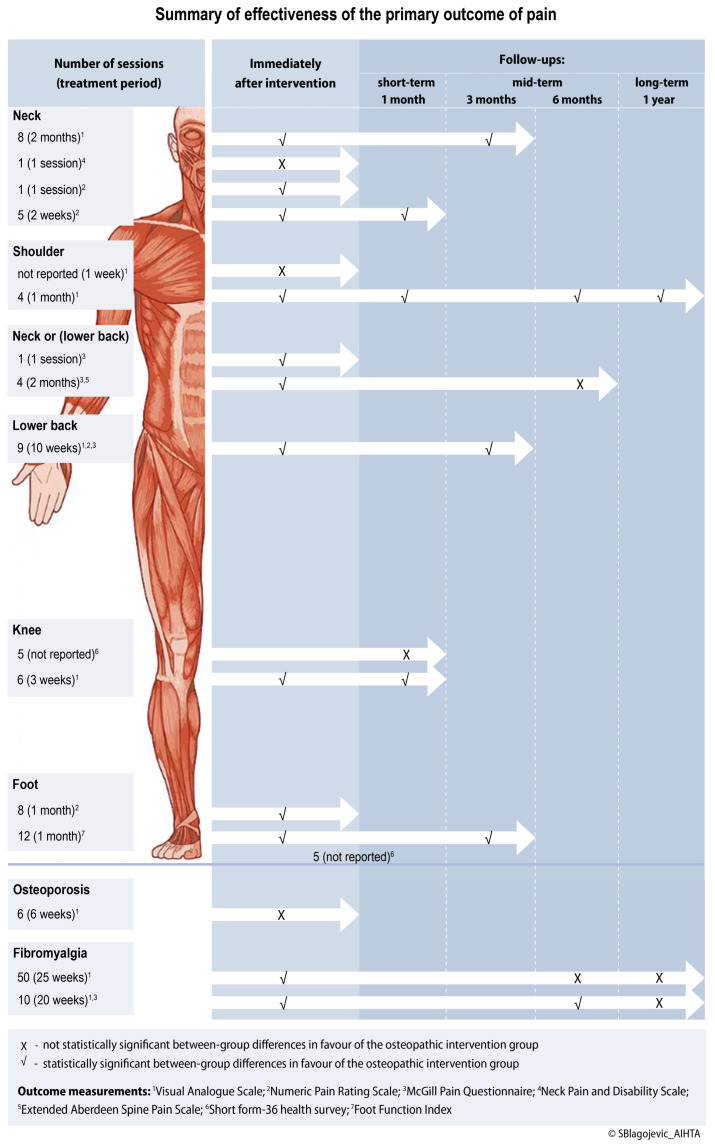
Summary of the effectiveness of osteopathic interventions on the primary outcome of pain.

Table 3 summarises effectiveness outcomes by region and time point, where ✓ denotes a statistically significant between-group difference favouring the intervention, X denotes no significant difference, and ‡ denotes a clinically meaningful intra-group improvement. Mean group differences and effect sizes, where available, are reported in the Supplementary Materials (Tables S13–S21). Figure 2 summarises the effectiveness of the primary outcome of pain.

### 3.3. Neck Pain

Four RCTs (unclear to high RoB) reported improvements in pain intensity immediately post-intervention [47,48,51,52]; the only study that found no improvement assessed a single treatment session [51]. Statistically significant between-group differences were found at short-term [47] or mid-term FU [48] in one study each, with clinically meaningful intra-group improvements in three of four studies [47,48,52]. Further pain-related outcomes (pain on movement [48], myofascial trigger points [52], pressure pain thresholds [47]) partially improved post-treatment or at FU; pressure pain sensitivity and cervical joint ROM showed no significant between-group differences. Functional and QoL outcomes were heterogeneous.

### 3.4. Shoulder Pain

Two RCTs (unclear to high RoB) assessed shoulder pain. One study (four sessions over four weeks, FU up to one year) reported improvements in pain, shoulder pain and disability at all time points [56]. The other (unspecified sessions within seven days, no FU) found no significant between-group difference immediately post-treatment, though a clinically meaningful intra-group improvement was observed at seven days [53]. ROM did not improve in either study. The remaining outcomes (arm, shoulder, and hand disability; activity and functionality) were heterogeneous [56].

### 3.5. Neck or (Low) Back Pain

Two RCTs (high RoB) assessed a mixed neck/low back pain population. One found a significant between-group pain reduction [55]; the other reported improvement in spinal pain and disability immediately post-treatment but no significant differences at mid-term FU [49]. Mental health improved significantly in one study post-treatment and at mid-term FU; physical health and QoL showed no significant between-group differences [49].

### 3.6. Low Back Pain

One systematic review of ten RCTs (high overall confidence [13]) found that pain was significantly reduced immediately after an average of nine osteopathic sessions over ten weeks, and remained improved at mid-term FU. Functional status improved post-treatment but not at mid-term FU.

### 3.7. Knee Pain

Two RCTs (unclear RoB) assessed five [59] and six sessions within three weeks [57], both with a one month FU. One study found significant pain reductions immediately post-treatment and at short-term FU, with a clinically meaningful intra-group difference [57]; the other found no improvement in general health, including bodily pain, at short-term FU [59]. Functionality and plantar pressure improved at both time points in one study [57]; the remaining outcomes were heterogeneous or non-significant [59].

### 3.8. Foot Pain

Two RCTs (unclear RoB) applied eight [60] and twelve sessions [58] over four weeks; one included a mid-term FU [58]. Pain intensity, pressure pain thresholds, and pain/disability/activity restriction improved significantly post-intervention and at mid-term FU [58]. A clinically meaningful reduction in pain was also observed in the other study [60].

### 3.9. Osteoporosis

One RCT (high RoB) assessed outcomes immediately after six OMT sessions over six weeks. The primary pain outcome did not improve; however, the pain subscale of the QoL instrument showed a reduction, and overall QoL, health perception, and path/mobility improved. Mental well-being, daily activities, housework, and leisure activities showed no significant improvements [50].

### 3.10. Fibromyalgia

Two RCTs (unclear to high RoB) applied 50 sessions over 25 weeks [54] and ten sessions over 20 weeks [44], both with FU to one year. Significant between-group pain differences were found post-treatment and at mid-term FU in one study [44], with clinically meaningful improvements on the VAS and McGill Pain Questionnaire. In the other study, pain was reduced only immediately post-treatment [54]. QoL subscales (physical and social function, general health, vitality—but not emotional role or mental health) improved post-treatment but not at FU in one RCT [54]; sleep quality results were heterogeneous, with some improvements persisting at mid- and long-term FU [54]. In the other RCT, significant between-group differences were observed for physical functioning, mood, fatigue, stiffness, clinical severity, and clinical improvement—though not all persisted to long-term FU—but not for postural stability or tiredness on walking [44].

A narrative comparison of pain outcomes between included and excluded studies is presented in the Discussion. No substantial discrepancies were identified, except in the foot region, suggesting no major reporting bias in the included evidence.

### 3.11. Professionals and Intervention Types

Osteopathic techniques were delivered by osteopaths (four studies [50,55,56,57]), physiotherapists with advanced osteopathic qualifications (three studies [44,48,58]), a physiotherapist (one study [53]), a therapist with osteopathic certification [47], a therapist [60], an expert craniosacral therapist [54], medical students from a Department of Osteopathic Manipulative Medicine [59], and general practitioners with osteopathic training [49,51]. Two studies did not report on the operator’s profession [13,52].

The most frequently applied technique was myofascial release [44,47,53,58,60], followed by craniosacral therapy [48,54] and OMT [50,57]. Additional techniques included strain–counterstrain [51], pressure release [52], fascial release [55], osteopathic spinal manipulation [49], and muscle energy technique [56]. One RCT applied one or a combination of myofascial release, strain–counterstrain, muscle energy, soft tissue, high-velocity low-amplitude, and craniosacral techniques [59].

### 3.12. Safety

Ten of 16 studies reported on adverse events [13,44,47,48,49,50,51,54,56,58]. For neck pain, two of three reporting studies found no serious adverse events [48,51]; minor adverse events (pain, shivering, tiredness, strong emotional reactions, weeping, dizziness) occurred in a small number of patients [48,51]. For low back pain, the systematic review reported increased pain in ten subjects and increased back muscle spasticity on one occasion across ten RCTs; one study did not collect adverse event data, and the remaining seven studies did not report adverse events [13]. No side effects attributable to osteopathy were reported in any of the 16 included studies.

### 3.13. Updated Search (2022–July 2025)

Twelve RCTs were identified across five regions: neck (*n* = 8) [25,61,62,63,64,65,66,67], shoulder/elbow (*n* = 1) [68], foot (*n* = 1) [69], general musculoskeletal complaints (*n* = 1) [70], and fibromyalgia (*n* = 1) [71] (Table 4, Table 5, Table 6, Table 7, Table 8 and Table 9). As no RoB assessment was conducted, findings are reported descriptively for exploratory purposes only and cannot be directly compared with the primary analysis.

Four of eight neck pain studies found statistically significant between-group improvements favouring osteopathic intervention [25,61,63,66]; four found no significant differences [62,64,65,67]. The shoulder/elbow study reported significant pain improvements favouring osteopathic intervention (myofascial release plus conventional physiotherapy vs. kinesiotaping) [68]. The foot study found no significant between-group improvement after 12 myofascial release sessions over four weeks [69]. One study reported significant improvement in pressure pain threshold after 24 pompage sessions over eight weeks, compared with no treatment [70]. The fibromyalgia study found no significant between-group improvements for craniosacral therapy compared to Bowen therapy, static touch, or standard exercise over 12 weeks [71].

In the neck area, no serious adverse events were reported across the eight included studies of the updated search. In one study [25], a total of 187 adverse events occurred across 298 OMT sessions, of which 37 were classified as at least “possibly related” to treatment. One adverse event—increased rib pain—was rated as severe. The remaining related adverse events were mild to moderate and included increased neck pain (*n* = 16), muscle soreness (*n* = 15), headache (*n* = 2), and other unspecified events (*n* = 3). One study [62] stated that no adverse events occurred during the follow-up period. The remaining studies on neck pain did not report or discuss adverse events [61,63,64,65,66,67]. For fibromyalgia, no serious adverse events occurred in any treatment group throughout the study period. However, two patients in the craniosacral therapy group experienced tiredness and a mild increase in pain lasting for one day following treatment [71]. The remaining studies included in the updated search did not report or discuss adverse events, including those addressing shoulder and elbow [68], foot [69], and musculoskeletal complaints [70].

Overall, updated search findings are consistent with the direction of the primary analysis; results for the neck region are marginally less favourable than those of the primary search.

## 4. Discussion

Based on current evidence, osteopathy can reduce neck and low back pain for up to three months and may reduce shoulder and foot pain; no definitive conclusions can be drawn for other body regions. No statistically or clinically significant deteriorations were attributable to osteopathic interventions. The absence of serious adverse events, the very low rate of minor adverse events, and the absence of reported side effects support osteopathy as a safe therapeutic option, though safety terminology was not consistently defined across primary studies.

The overall quality of evidence must be treated with caution: none of the included trials achieved a low RoB; RoB in the RCTs was unclear (*n* = 8) or high (*n* = 7). The systematic review on chronic non-specific low back pain [13] was rated as having high overall confidence, yet none of its constituent RCTs demonstrated low RoB. The primary limitation across studies was the inability to blind patients and treating practitioners—a constraint inherent to manual interventions. Five of the 15 included RCTs used double-blind designs, employing strategies such as disconnected ultrasound as a sham treatment or lead-in periods that prevented clinicians from knowing the allocation sequence. Statistically significant and some clinically meaningful pain reductions were observed; however, these findings must be interpreted with caution, given the overall moderate quality of evidence.

Beyond the 16 included studies, 20 thematically relevant RCTs were excluded due to lower methodological quality. To avoid missing substantive findings, the primary pain outcome was extracted from these excluded studies and compared narratively with included results. No fundamental discrepancies were identified, with the exception of the foot region, where results among excluded studies were inconsistent: one excluded study showed no between-group improvement after a single OMT session versus standard care [24], while another demonstrated pain reduction after 16 sessions [23], suggesting that a single session may be insufficient for foot pain.

The systematic review on low back pain [13] searched the literature until April 2020, leaving a two-year gap relative to the primary search. A supplementary search for this period identified three relevant RCTs [72,73,74]: two reported significant between-group pain improvements favouring osteopathy [72,74], and one showed no significant between-group differences, though both groups improved significantly from pre- to post-treatment [73]. A recent systematic review and meta-analysis examining whether OMT is clinically superior to sham or placebo for low back or neck pain reported no statistically significant differences in pain intensity across 1173 patients [14].

Neck and low back pain represent the leading causes of years lived with disability, accounting for 5.6% of all disability-adjusted life years in 2019 [75]. Consistent with this epidemiological burden, the majority of identified studies addressed these regions [76]. Evidence for other regions—particularly the shoulder and foot—remains sparse and warrants further investigation.

For fibromyalgia, multiple diagnostic criteria exist (e.g., American College of Rheumatology (ACR) criteria [77]), and evidence suggests possible neuropathic mechanisms or coexistence with neuropathic pain [78,79], which may complicate the interpretation of results. Future research should apply more specific diagnostic tools to classify and stratify fibromyalgia patients, including by degree of neuropathic involvement.

Considerable heterogeneity across included RCTs—in professional background, sample sizes, blinding procedures, comparators, number of sessions, treatment duration and frequency, and FU intervals—may have influenced results. Pain outcomes were predominantly assessed using subjective patient-reported measures such as VAS and NRS, which are accepted as valid given the inherently subjective nature of pain [80]. Clinically meaningful improvements were observed in seven of 15 included RCTs; reported minimal clinically important difference values varied widely (e.g., 1.4–5.2 cm for VAS [81,82,83,84]).

Regarding intervention heterogeneity, a single isolated technique may not constitute osteopathy in the full clinical sense, as osteopathic practice involves individualised technique selection based on palpatory findings rather than a fixed protocol. Techniques such as myofascial release are also applied by physiotherapists and manual medicine practitioners, complicating attribution of effects. Nonetheless, all osteopathic techniques share the therapeutic aim of promoting optimal tissue function to restore the body’s regulatory capacity. Variability in technique, dosage, and treatment duration is an inherent feature of person-centred osteopathic practice, though it limits precise estimation of the therapeutic contribution of osteopathy [13], consistent with Cochrane Handbook guidance against pooling when substantial clinical or methodological heterogeneity is present [46].

The decision not to conduct a meta-analysis requires explicit justification. Although pain was the primary outcome across all regions, pooling was precluded by five sources of heterogeneity: populations varying in pain chronicity and diagnostic criteria; interventions differing in technique, session number (1–50), and practitioner background; comparators ranging from no treatment to active physiotherapy; outcome instruments that differed even within the same region; and only one to four RCTs per body region. Pooling under these conditions would risk producing a statistically precise but clinically misleading estimate, consistent with established guidance on the conditions required for meaningful meta-analysis [46]. Future reviews may be better positioned to conduct subgroup meta-analyses as the evidence base within individual regions grows and interventions become more standardised.

A related concern is construct validity. Several included techniques—notably myofascial release and craniosacral therapy—are not exclusive to osteopathic practice and are routinely applied by physiotherapists and other manual therapists. This review did not isolate osteopathy-specific effects from technique effects—doing so would require head-to-head comparisons between professional groups applying identical protocols, a design absent from the current evidence base. Findings should therefore be interpreted as evidence for the effectiveness of osteopathic techniques as delivered within osteopathic practice, rather than as evidence for osteopathy as a uniquely differentiated discipline. Future research should address this by using comparative effectiveness designs that explicitly examine the role of practitioners’ professional backgrounds.

Control group heterogeneity—ranging from no treatment and waiting lists to sham, placebo, and active comparators—may have influenced observed effect sizes, as no-treatment controls tend to produce larger apparent effects than active comparators. Specific, non-specific (e.g., therapeutic alliance), and contextual factors may contribute to observed effects in both conditions, particularly in sham-controlled trials [85]. The qualitative synthesis did not reveal systematic differences in results across control group types, consistent with findings from the low back pain review [13]. Future research should employ well-defined, standardised control groups, multiple control arms where feasible, and designs that enable subgroup analyses. Network meta-analyses or meta-regression in future systematic reviews would allow more rigorous exploration of control group heterogeneity.

Regarding treatment dosage, sessions ranged from one [13,51,52,55] to 50 [54] across a treatment period of one session to 25 weeks. Short-term FU (1 month) was assessed in four studies, mid-term FU (3–6 months) in seven, and long-term FU (1 year) in three. Although improvements were detected after a single session, optimal treatment intensity and duration remain undetermined. Patient behaviours between the end of treatment and FU (e.g., exercise, additional manual therapy, medication) represent an uncontrolled source of variability; future studies should document these activities systematically, for example, through patient logbooks.

Two included RCTs applied OMT. A recent overview of systematic reviews found OMT more effective than comparators for reducing pain and improving functional status in musculoskeletal disorders, with no adverse events reported in most included reviews [86]. Despite the near absence of methodologically robust evidence for craniosacral therapy [9], effects were observed in three included studies across all FU intervals. High-velocity low-amplitude techniques were applied in one knee pain study; published literature supports their role in pain modulation in musculoskeletal disorders [87].

The majority of included studies were conducted in Europe (Spain, Italy, Germany, the UK, and Poland); five RCTs were from outside Europe (Australia, Brazil, India, the USA, and Qatar), which may limit generalisability. Osteopathic practice in the USA differs substantially from elsewhere: US osteopathic practitioners train as physicians before specialising in OMT, whereas European and Australasian training focuses on OMT without conferring medical licensure [6]. Given the relevance of psychosocial factors alongside physical factors in several included studies [3], osteopathic treatment should be considered a component of a comprehensive, multimodal treatment plan [13].

The updated search identified 12 additional RCTs whose findings are reported in Section 3. Overall, results are consistent with the primary analysis. For the neck region, findings are marginally less favourable, potentially reflecting greater heterogeneity in intervention protocols and comparator groups in more recent trials. As no RoB assessment was conducted for the updated search studies, their findings must be treated as contextual and exploratory only.

## 5. Limitations

This review employed a hybrid design, combining a de novo systematic search of RCTs for most body regions with a pre-specified umbrella review component for chronic non-specific low back pain. Although this approach was justified a priori and registered in PROSPERO, the evidence base for low back pain differs from that of other regions in its assembly: the pre-specified review [13] applied slightly different inclusion thresholds and searched the literature only until April 2020, limiting direct comparability across regions.

The selection of best available evidence per body region—retaining 15 of 35 eligible RCTs based on RoB profile via a two-step selection procedure—departs from conventional systematic review practice, which typically includes all eligible studies and addresses quality through sensitivity analyses. This approach was chosen within the HTA framework to prioritise internal validity. To mitigate the risk of missing important findings, pain outcomes from all 20 excluded studies were extracted and compared narratively; no substantial discrepancies were identified, except in the foot region.

The updated search was conducted without a formal RoB assessment, which deviated from PRISMA reporting standards and from the methodology applied in the primary analysis. This limits the interpretability of updated search findings and precludes formal reassessment of overall evidence certainty. Future updates should apply a full RoB assessment to all identified studies.

Additional deviations from uniform inclusion criteria are documented as deviations from the pre-registered PROSPERO protocol: low back pain was addressed via umbrella review; a two-step best-available-evidence approach was performed to ensure coverage across all body regions, with the second step applying a minimum threshold of one domain rated ‘high risk’ and one domain rated ‘unclear’, a criterion defined post hoc by the review authors as a pragmatic minimum standard; and the 50 participant threshold was not applied to the pre-specified low back pain review [13]. Applying the 50 participant threshold may have reduced comprehensiveness; although it was intended to minimise overestimation of treatment effects [21], the same authors have recommended sensitivity analyses rather than outright exclusion.

No single osteopathic technique was prioritised or mandated as the primary intervention; all eligible techniques were included regardless of type, which precludes direct comparative effectiveness assessment between techniques, equivalence claims across techniques, and conclusions on cross-country consistency in their application. Evidence quality was evaluated using the Cochrane RoB tool (version 1) and AMSTAR 2, consistent with HTA methodology [42]; a formal GRADE assessment was not performed. This decision was deliberate: no included RCT achieved a low overall risk of bias, necessitating downgrading in this domain for all outcomes, and the substantial heterogeneity precluding meta-analysis would have required additional downgrading for inconsistency, likely resulting in very low certainty ratings across most outcomes.

## 6. Conclusions

Based on current evidence, osteopathy can improve neck and low back pain in the short and mid-term and may reduce shoulder and foot pain; evidence for other body regions and conditions remains uncertain. The overall certainty of evidence is low to moderate, and findings should therefore be interpreted cautiously.

From a clinical perspective, osteopathy may be considered as one component within a multimodal treatment approach for patients with neck or low back pain, particularly where first-line treatments have provided insufficient relief. The consistent absence of serious adverse events across all included studies supports its safety profile and may inform shared decision-making between practitioners and patients.

At the policy level, these findings are relevant to HTA and guideline development, given the integration of osteopathy into primary healthcare systems, particularly in countries where formal recognition is under discussion. High-quality research with rigorous study designs, standardised interventions, mid- and long-term follow-ups, and a systematic focus on technique, treatment dosage, and safety reporting is needed to strengthen the evidence base and support evidence-informed reimbursement decisions.

## Figures and Tables

**Table 1:** Inclusion criteria (all body regions and diseases except for lower back).

Description	Project Scope
**P**opulation	Adults (male, female; over 18 years) with musculoskeletal pain in various body regions and diseases Inclusion: musculoskeletal pain (back/neck/cervical/shoulder/pelvic/ankle pain, etc.), osteoarthritis, shoulder impingement syndrome, epicondylopathia, epicondylitis, rheumatic conditions in the musculoskeletal system (ankylosing spondylitis, fibromyalgia, etc.), postoperative pain ^1^, chronic/persistent pain, adhesive capsulitis, piriformis syndrome Exclusion: chronic low back pain ^2^, migraine/headache, carpal tunnel syndrome, neurofibromatosis, cancer patients/survivors, pregnant/postpartum women, patients with dysmenorrhea, prostatitis, hemophilic arthropathy, bruxism, suboccipital tenderness, temporomandibular disorder ^3^
**I**ntervention ^4^	Osteopathy Operational definition: Any osteopathic technique (i.e., single technique or in combination with other techniques) alone or in addition to other interventions delivered by osteopaths or other therapists (i.e., osteopaths or non-osteopaths) Inclusion ^5^: craniosacral therapy/treatment, osteopathic manipulative treatment/medicine, cranial osteopathy, myofascial release, osteopathic visceral manipulation, dry needling ^6^, strain—counterstrain technique, high-velocity low-amplitude (spinal) manipulation, thrust manipulation, (Spencer) muscle energy technique, soft tissue technique, pressure release technique, spinal manipulative treatment, lumbopelvic manipulation Exclusion: proprioceptive neuromuscular facilitation, chiropractic, electrotherapy treatment ^7^, self-applied interventions/techniques (e.g., self-myofascial release)
**C**ontrol	Standard care, no therapy, or alternative therapy (e.g., waiting list, no care, sham treatment, massage, physiotherapy, conservative therapy, pharmacological treatment, other non-surgical treatment) Exclusion: surgical treatment
**O**utcomes	Effectiveness Primary outcome: ■ Pain ^8^ Secondary outcomes: ■ Physical/Mental/General health ■ Functional status ■ Mobility restriction ■ Range of motion (ROM)/Stiffness ■ Symptom improvement ■ Quality of life (QoL) ■ Fatigue ■ Body awareness ■ Depression/Anxiety Safety ■ Adverse events ^9^ ■ Side effects ^10^
**S**tudy design Publication period Languages Sample size	RCTs From inception until May 2022 ^11^ English, German Abstract screening: ≤25 patients excluded; full texts assessment: ≤50 patients excluded ^12^

^1^ Postoperative pain is methodologically unsuitable as it might be too heterogeneous to create a comparable patient group dynamic, and is unpredictable in its course, difficult to control regarding the use of rescue medication, and is influenced by routine clinical procedure, causing noisy data due to high variability, which weakens the study’s statistical power. ^2^ Chronic low back pain is not the focus of our report; however, it is covered by a systematic review and meta-analysis to give an overall picture (see Section 2). ^3^ While temporomandibular disorders share specific pain mechanisms with conditions such as fibromyalgia, pain from rheumatic diseases, or low back pain, their exclusion from this study reflects a conscious decision to narrow our focus. This enables us to facilitate clinicians in identifying patient subgroups based on distinct pain mechanisms within a more specific set of conditions, typically treated by a particular group of specialists or characterised by pain in specific body regions. We aim to ensure the internal validity and practical manageability of the study, as we believe comprehending the neurobiological underpinnings of pain within these chosen conditions is crucial for optimising clinical outcomes in their respective contexts. ^4^ Manipulation techniques can be seen as an umbrella term for manual joint interventions. Spinal manipulation is a more focused type of manipulation. In contrast, chiropractic refers to a distinct profession whose core technique is a specialised form of spinal manipulation, often involving high-speed thrusts. ^5^ In the introduction, only interventions are described which are applied by operators in the included studies. ^6^ Dry needling is frequently used by osteopaths and other manual therapists and incorporated into their practice as an adjunctive or complementary tool [17,18]. Furthermore, we ensure that the primary interventions are manual therapy techniques consistent with osteopathic principles. If dry needling as a component of a broader osteopathic intervention is incorporated by any study, we will highlight this in our discussion and acknowledge the potential for confounding. However, studies where dry needling was the sole or primary intervention were generally excluded, as they would fall outside the scope of ‘manual therapy’ for the purposes of this review. ^7^ Electrotherapy treatment involves the application of a therapeutic electrical current to the area of injury, inflammation, dysfunction, or pain. ^8^ All outcome measurements were taken into account. ^9^ An adverse event is defined as “a harmful or undesirable outcome that occurs during or after the use of a drug or intervention but is not necessarily caused by it. When causality is uncertain or the purpose of the relative effectiveness assessment is to establish causality, “adverse event” should generally be the default term over “adverse effect” or “adverse reaction/adverse drug reaction” [19]. ^10^ This unintended effect “does not necessarily imply harm, as some side effects may be beneficial. Furthermore, it tends to understate the importance of harms because “side” may be perceived as denoting secondary importance” [20]. ^11^ May 2022, as the literature search was set for this month. ^12^ Generalisability from small samples is problematic and may produce inconsistency and overestimation of effects.

**Table 2:** Information regarding number of papers, total number of subjects per each body region and disease, number of randomised patients (age mean (SD)), dropout rates, and countries.

Body Region and Disease	Number of Papers	Number of Randomised Patients (Total)	Number of Randomised Patients (Age Mean (SD))	Dropout Rate	Countries
Neck	Four RCTs	244	54 (81.5% female; 44.6 ±10.0) [48] 61 (45 female; IG: 47.9 (10.1); CG: 41.9 (10.4)) [51] 75 (60 female; 20–55) [52] 54 (26 female; inclusion criteria: 20–60) [47]	Lost to assessment at week 8: 3 Lost to assessment at week 20: 9 [48] 0 [47,51,52]	Germany [48,51] Spain [47,52]
Neck or (lower) back	Two RCTs	321	120 (IG: 18 female; 21–58; CG: 18 female; 18–56) [55] 201 (female: NR; 16–65) [49]	NR [55] 18 [49]	Italy [55] UK [49]
Shoulder	Two RCTs	135	60 (31 female; 20–55) [53] 75 (25 per group) (IG: 10 female; 62.0 ± 9.6; placebo group: 9 female; 61.4 ± 11.3; muscle energy technique + soft tissue massage group: 9 female; 56.9 ± 9.2) [56]	0 [53] 18 (until last FU) [56]	India [53] Australia [56]
Lower back	One systematic review and meta-analysis	1160	1160 (female: NR; mean age 43.3 +/− 7.7) [13]	Range: 0–77% [13]	Italy [13]
Knee	Two RCTs	142	82 (48 female; 18–35) [57] 60 (42 female; 69.2 (10.3)) [59]	Withdrawal from eligible patients: 5 [57] Loss to 4 week postdischarge FU: 8 [59]	Brazil [57] USA [59]
Foot	Two RCTs	136	70 (47 female after dropout; 20–49) [60] 66 (49 female; IG: 42.4 ± 4.6; CG: 40.8 ± 7.1) [58]	10 [60] 1 [58]	Poland [60] Qatar [58]
Osteoporosis	One RCTs	72	72 (51 female; IG: 77.2 (5.3); CG: 76.8 (8.2)) [50]	0 [50]	Italy [50]
Fibromyalgia	Two RCTs	198	Randomised: 104 Analysed: 84 (81 females; range 34–63; mean 49.08 ± 14.17) [54] 94 (female: NR; range 45–65; mean 54.4) [44]	20 [54] 8 [44]	Spain [44,54]

**Table 3:** Summary of effectiveness outcomes, test points, number of sessions, treatment period, professionals involved, and type of osteopathic interventions.

Outcomes	Time of Testing, Group Difference (✓ s.s./X n.s.)					Number of Sessions (Treatment Period)	Intervention Applied by (Profession)	Type of Osteopathic Intervention	Comparison	[Ref]
	Immediately After Intervention: 0–7 Days After End of Treatment	Short-Term FU: 1 Month FU	Mid-Term FU		Long-Term FU: 1 Year FU					
			3 Months FU	6 Months FU						
**Neck**										
Pain intensity	✓ ‡		✓ ‡			8 (8 weeks) ^1^	Physiotherapists with advanced craniosacral therapy qualification	Craniosacral therapy	Light-touch sham treatment	[48]
Pain on movement	✓		✓							
Point of max. pain	✓		X							
Pain acceptance	X		X							
Pressure pain sensitivity:										
Musculus levator scapulae	X		X							
Musculus trapezius	✓		X							
Musculus semispinalis capitis	X		X							
Physical health:										
Functional disability	✓		✓							
Physical QoL	✓		✓							
Physical well-being	X		X							
Mental health:										
Mental QoL	X		X							
Anxiety	X		✓							
Depression	X		X							
Stress perception	X		X							
Body awareness:										
Body awareness	✓		X							
Body dissociation	X		X							
Global improvement	✓		✓							
Pain intensity	X					1 (1 session) ^2^	General practitioner with completed full osteopathic curriculum	Strain—counterstrain treatment	Sham treatment	[51]
Mobility restriction	X									
Level of pain (subjective pain)	✓‡					1 (1 session)	NR	Pressure release	Kinesiotaping; placebo	[52]
Myofascial trigger points of sternocleidomastoid muscle right/left (objective pain)	✓									
Cervical joint range (objective pain)	X									
QoL	X									
Pain intensity	✓ ‡	✓ ‡				5 (2 weeks) ^3^	Therapist with experience and certificate in myofascial release therapy	Myofascial release	Standard physical therapy	[47]
Pressure pain thresholds:										
Suboccipita left/right	✓	✓								
Thoracic left	X	X								
Thoracic right	✓	✓								
Cervical active ROM:										
Flexion	X	X								
Extension	X	X								
Side bending left/right	X	X								
Rotation right	X	✓								
Rotation left	✓	✓								
**Neck or (lower back)**										
Pain	✓					One session (1 session) ^4^	Osteopath	Fascial release	Sham treatment	[55]
Spinal pain and disability	✓			X		4 (2 months) ^5^	General practitioner registered as osteopath	Osteopathic spinal manipulation	Usual care	[49]
Pain	X			X						
Physical health	X			X						
Mental health	✓			✓						
QoL	X			X						
**Shoulder**										
Pain	X ‡					NR (7 days) ^6^	Physiotherapists	Myofascial release	Active release technique	[53]
ROM: Cervical flexion/extension, cervical side flexion (right/left) and cervical rotation (right/left)	X									
Neck disability	X									
Pain	✓	✓		✓	✓	4 (4 weeks) ^7^	Osteopath	Muscle energy technique	Muscle energy technique + soft tissue massage; placebo	[56]
Shoulder pain and disability	✓	✓		✓	✓					
Arm, shoulder and hand disability	✓	X		✓	✓					
Change in activities	✓	✓		X	X					
Activity/functionality	✓	X		X	✓					
ROM (standing posture, thoracic flexion, thoracic extension, total thoracic ROM)	X									
**Lower back**										
Pain	✓		✓			9 (10 weeks) ^8^	NR	Osteopathic interventions	No active treatment (sham therapy or no intervention; *n* = 5), active treatment (standard exercise, classic massage; *n* = 5)	[13]
Functional status	✓		X							
**Knee**										
Functional independence	X					5 (NR) ^9^	Osteopathic medical students	One or a combination of: myofascial release, strain—counterstrain, muscle energy, soft tissue, high-velocity low-amplitude (not at the surgical site), or craniosacral manipulation	Sham treatment (range of motion activities, light touch)	[59]
Daily analgesic medication use	X									
Length of stay	X									
Rehabilitation efficiency	✓									
General health (physical functioning, physical role limitations, bodily pain, general health, vitality, social functioning, emotional role limitations, mental health)		X ^10^								
Pain	✓ ‡	✓ ‡				6 (3 weeks) ^11^	Osteopath	OMT	Exercise programme; Waiting list	[57]
Functionality	✓	✓								
Dynamic knee valgus	✓	X								
Plantar pressure in middle foot	✓	✓								
Posterior thigh flexibility	✓	X								
Hip ROM	X	X								
**Foot**										
Pain intensity (left/right foot)	✓ ‡					8 (4 weeks) ^12^	Therapist	Myofascial release	Exercise programme; myofascial release and exercise programme ^13^; no intervention	[60]
Pain, disability and activity restriction	✓		✓			12 (4 weeks) ^14^	Physiotherapists certified in myofascial release	Myofascial release	Sham ultrasound therapy	[58]
Pressure pain thresholds (gastrocnemius, soleus and calcaneus)	✓		✓							
**Osteoporosis**										
Pain	X					6 (6 weeks) ^15^	Osteopath	OMT	Sham manipulative treatment	[50]
QoL	✓									
QoL-subscales:										
Pain	✓									
Perception of health	✓									
Path/mobility	✓									
Mental well-being	X									
Daily activities	X									
Housework	X									
Leisure activities	X									
**Fibromyalgia**										
Pain	✓			X	X	50 (25 weeks) ^16^	Expert craniosacral therapist	Craniosacral therapy	Placebo (simulated treatment with disconnected ultrasound)	[54]
State anxiety	X			X	X					
Trait anxiety	✓			X	X					
Depression	X			X	X					
QoL:										
Physical function	✓			✓	X					
Physical role	✓			X	X					
Body pain	✓			X	X					
General health	✓			X	X					
Vitality	✓			✓	X					
Social functioning	✓			X	X					
Emotional role	X			X	X					
Mental health	X			X	X					
Sleep quality:										
Subjective sleep quality	✓			X	X					
Sleep latency	X			X	X					
Sleep duration	✓			✓	✓					
Habitual sleep efficiency	X			✓	✓					
Sleep disturbance	✓			✓	X					
Daily dysfunction	X			X	✓					
Pain (MPQ)	✓ ‡			✓ ‡	X ‡	10 (20 weeks) ^17^	Physiotherapist specialised in myofascial therapy	Myofascial release	Sham short-wave and ultrasound electrotherapy	[44]
Pain: sensory	✓			✓	✓					
Pain: affective	✓			✓	X					
Pain: sensory + affective	✓			✓	✓					
Pain (VAS)	✓‡			✓	X					
Physical functioning	✓			✓	X					
Mood	✓			✓	✓					
Fatigue	✓			✓	✓					
Tiredness on walking	✓			X	X					
Stiffness	✓			✓	X					
Clinical severity	✓			✓	X					
Clinical improvement	✓			✓	✓					
Postural stability	X			X	X					

Abbreviations: ✓, statistically significant improvement favouring the osteopathic intervention group. X, no statistically significant difference. ‡, clinically meaningful improvement in the osteopathic intervention group. FU, follow-up. max., maximum. MPQ, McGill Pain Questionnaire. NR, not reported. OMT, osteopathic manipulative treatment. QoL, quality of life. ROM, range of motion. s.s., statistically significant. n.s., not statistically significant. VAS, visual analogue scale. ^1^ “Outcomes were assessed before and after treatment (week 8) and again 3 months later (week 20).” ^2^ “After receiving the allocated treatment patients underwent a second measurement.” ^3^ Patients were assessed “at the end of treatment and at 1 month follow-up.” ^4^ Patients were assessed 3 days after the session. ^5^ Patients were assessed “before randomization, after 2 months when treatment in the intervention group was complete, and finally after 6 months.” ^6^ Patients were assessed “on seventh day following intervention.” ^7^ “Measures (were) recorded at baseline, discharge, 4 week follow-up, 6 months, and 12 months.” ^8^ Presented in means. 12 weeks follow-up. ^9^ Measures were taken from rehabilitation unit admission to discharge, at rehabilitation unit discharge, from rehabilitation unit admission to 4 weeks after discharge. ^10^ “The research coordinator subsequently conducted SF-36 telephone interviews 4 weeks after discharge from the rehabilitation unit.” ^11^ Patients were assessed “before the interventions, after the six interventions, and at 30 day follow-up.” ^12^ Patients were assessed before and after therapy. ^13^ The control group ‘myofascial release and exercise programme’ was not compared in this report because it includes an osteopathic technique. ^14^ Measures were taken at “week 1 (pretest score), week 4 (post-test score), and follow-up at week 12 after randomization.” ^15^ Patients were assessed at the first and sixth session of treatment. ^16^ Outcomes “were determined at baseline and at 10 min, 6 months and 1 year post-treatment.” ^17^ Patients were assessed after 20 weeks of myofascial therapy, at six months post intervention and at one year post intervention.

**Table 4:** Excerpt extraction table: summary of effectiveness on pain (neck part 1/2).

Author, Year [Reference]	Cholewicki, 2022 [25]	Deshmukh, 2022 [61]	Groisman, 2023 [62]	Iakovidis, 2023 [63]
Indication	Chronic non-specific neck pain	Non-specific neck pain	Non-specific chronic neck pain	Neck myofascial syndrome
Number of randomised patients	97	100	90	80
Intervention/technique	OMT	MFR	OMT plus exercises	MFR
Intervention applied by (profession)	Osteopathic physicians specialised in OMT	NR	Registered osteopaths	Physical therapist
Comparison	Waiting list	Basic exercise therapy	Exercises	MFR plus transcutaneous electrical nerve stimulation conductive glove, conventional transcutaneous electrical nerve stimulation, placebo transcutaneous electrical nerve stimulation
Effectiveness outcomes	After 3–4 sessions over 4–6 weeks: Average pain: IG vs. CG (95% CI): −1.02 (−1.72, −0.32), ***p* = 0.005** Current pain: IG vs. CG (95% CI): −1.02 (−1.75, −0.30), ***p* = 0.006**	3×/week treatment for 1 week: Pain intensity: after 1 week: IG vs. CG: t-value: 2.14, ***p* = 0.037** Pressure pain threshold: after 1 week: IG vs. CG: t-value: 0.68, *p* = 0.5	One OMT session/week for 4 weeks: Pain intensity: IG vs. CG: (mean ± SE CI(95%): 3 months: −0.9 ± 0.5 (−2.0 to 0.1), *p* = 0.1, 6 months: 0.6 ± 0.7 (−0.8 to 1.9), *p* = 0.4 Pressure pain threshold: IG vs. CG: (mean ± SE CI(95%): 3 months: −0.1 ± 0.6 (−1.4 to 1.2), *p* = 0.8, 6 months: 0.4 ± 0.8 (−1.2 to 2.1), *p* = 0.6 Pain self-efficacy: IG vs. CG: (mean ± SE CI(95%), *p*-value: 3 months: 54.7 ± 40.4 (−24.5 to 134.05), *p* = 0.1; 6 months: −25.0 ± 25.2 (−74.4 to 24.4), *p* = 0.3	Six sessions over a period of 3 weeks: Pain intensity: between-group *p*-value: 3 weeks: **s.s.** between all groups, 1 month: **s.s.** between all groups Pressure pain threshold: between-group *p*-value: *3 weeks*: MFR+TENS vs. TENS: **s.s.**, MFR+TENS vs. MFR: **s.s.**, MFR+TENS vs. placebo: **s.s.**, MFR vs. placebo: **s.s.**, TENS vs. MFR: not s.s. *1 month*: MFR+TENS vs. TENS: **s.s.**, MFR+TENS vs. placebo: **s.s.**, MFR+TENS vs. MFR: not s.s., TENS vs. MFR: not s.s., MFR vs. placebo: not s.s.
Conclusion	“OMT is relatively safe and effective in reducing pain and disability along with improving sleep, fatigue, and depression in patients with chronic neck pain immediately following treatment delivered over approximately 4 to 6 weeks.”	“The study concluded that myofascial release technique is effective in reducing pain intensity, improving neck mobility in patients with nonspecific neck pain.”	“Outcomes of pain and functionality for patients in both groups were improved at 6 months. Our findings show that the combination of OMT and neck exercises for 4 weeks did not improve functionality and reduction in pain in patients with non-specific chronic neck pain.”	“The MFR protocol appears to be more effective in dealing with pain, disability, and lateral flexion range of motion than conventional transcutaneous electrical nerve stimulation. A transcutaneous electrical nerve stimulation conductive glove significantly improves the effects of MFR, possibly due to the combined mechanical and electrical stimulation of the muscle.”

Abbreviations: CG, control group. CI, confidence interval. IG, intervention group. MFR, myofascial release. NR, not reported. OMT, osteopathic manipulative treatment. s.s., statistically significant. SE, standard error. TENS, transcutaneous electrical nerve stimulation.

**Table 5:** Excerpt extraction table: summary of effectiveness on pain (neck part 2/2).

Author, Year [Reference]	Khan, 2022 [64]	Morsi, 2023 [65]	Overmann, 2024 [66]	Tahmaz, 2023 [67]
Indication	Non-specific neck pain	Chronic non-specific neck pain	Chronic neck pain	Non-specific neck pain
Number of randomised patients	60	54	128	115 ^1^
Intervention/technique	MFR	MFR	MFR	MFR
Intervention applied by (profession)	Therapist	Physiotherapist	Therapist	Physiotherapist
Comparison	Post-isometric relaxation	Sustained natural apophyseal glides, sustained natural apophyseal glides plus MFR	Placebo treatment involving sham laser therapy	Manipulation/Mobilisation treatment group
Effectiveness outcomes	Three sessions/week for 2 weeks: Pain intensity: mean between-group differences: 2 weeks: −0.7, ***p* = 0.008 (in favour of the CG)**	After 8 weeks of treatment: Pain intensity: MFR vs. sustained natural apophyseal glides: *p* = 0.99 Pain sensitivity (pressure pain threshold): MFR vs. sustained natural apophyseal glides: *p* = 0.97	Single 12 min session: Pain perception: IG vs. CG: F = 53.88, ***p* < 0.001** Pressure pain threshold left: IG vs. CG: F = 8.00 , ***p* = 0.005** Pressure pain threshold right: IG vs. CG: F = −4.91 , ***p* = 0.03**	Single 5 min session: Pain intensity: IG vs. CG: *p* = 0.906
Conclusion	“The study demonstrated patients with nonspecific neck pain can benefit from the post isometric relaxation with significant improvement in pain, disability, cervical range of motion, and quality of life compared with myofascial release therapy.”	“The findings of this study stressed the idea that the combined effect between sustained natural apophyseal glides and myofascial releases was more effective and promising than the unimodal methodology.”	“The findings suggest that myofascial release has a positive impact on individuals with chronic neck pain and depression, particularly in reducing pain intensity. Integrating myofascial release into treatment approaches may be beneficial. However, further research is needed to confirm and expand upon these findings, explore long-term effects, and better understand the clinical significance of certain outcomes.”	“A single session of myofascial release and manipulation/mobilization therapy has an immediate positive effect on pain, finger grip strength, spine alignment and grip strength. Manual therapy practices can be used for rapid symptom relief in patients with non-specific neck pain.”

Abbreviations: CG, control group. IG, intervention group. MFR, myofascial release. OMT, osteopathic manipulative treatment. ^1^ An error could be observed in the CONSORT 2010 flow diagram (Figure 1) as the authors stated 116 instead of 115 randomised patients.

**Table 6:** Excerpt extraction table: summary of effectiveness on pain (shoulder and elbow).

Author, Year [Reference]	Khanna, 2022 [68]
Indication	Lateral epicondylitis
Number of randomised patients	60
Intervention/technique	MFR plus conventional physiotherapy treatment
Intervention applied by (profession)	Therapist
Comparison	Kinesiotaping plus conventional physiotherapy treatment
Effectiveness outcomes	4 days/week for 4 weeks of intervention: Average pain and function: IG vs. CG: t-value: 6.16, *p* < 0.001 Pain intensity: IG vs. CG: t-value: 3.60, *p* < 0.001
Conclusion	“The study demonstrates that MFR is more effective in decreasing pain, functional disability and improving grip strength in lateral epicondylitis as compared to kinesiotaping.”

Abbreviations: CG, control group. IG, intervention group. MFR, myofascial release.

**Table 7:** Excerpt extraction table: summary of effectiveness on pain (foot).

Author, Year [Reference]	Akter, 2024 [69]
Indication	Plantar heel pain, plantar fasciitis, or calcaneal spur
Number of randomised patients	64
Intervention/technique	MFR
Intervention applied by (profession)	Specialist physiotherapist
Comparison	SDM approach
Effectiveness outcomes	12 sessions over 4 weeks: Pain: IG vs. CG: 12 weeks: mean difference: −0.349, *t* = −0.221, ***p* = 0.001 (in favour of the CG)**
Conclusion	“Both MFR and SDM approaches are effective in reducing pain, improving function, ankle range of motion, and reducing disability in plantar heel pain; however, the SDM approach may be a preferred treatment option.”

Abbreviations: CG, control group. IG, intervention group. MFR, myofascial release. SDM, structural diagnosis and management.

**Table 8:** Excerpt extraction table: summary of effectiveness on pain (musculoskeletal complaints).

Author, Year [Reference]	Andriollo, 2022 [70]
Indication	Female teachers with vocal and musculoskeletal complaints
Number of randomised patients	56
Intervention/technique	Pompage (MFR technique)
Intervention applied by (profession)	Physiotherapy students and previously trained physiotherapists
Comparison	No treatment
Effectiveness outcomes	24 sessions of 40 min each for 3×/week: Pain pressure threshold: IG vs. CG: 2 months after intervention start: **s.s.** in 9 of 12 muscles
Conclusion	“After myofascial release therapy with pompage, the subjects presented a reduction in cervical pain and in functional disability, an increase in pain threshold, and posture improvement.”

Abbreviations: CG, control group. IG, intervention group. MFR, myofascial release. s.s., statistically significant.

**Table 9:** Excerpt extraction table: summary of effectiveness on pain (fibromyalgia).

Author, Year [Reference]	Ughreja 2024 [71]
Indication	Fibromyalgia
Number of randomised patients	132
Intervention/technique	Craniosacral therapy
Intervention applied by (profession)	Certified physiotherapist trained in craniosacral and Bowen therapy
Comparison	Bowen therapy, static touch (placebo; standard exercise programme)
Effectiveness outcomes	1×/week 45 min sessions for 12 weeks: Pressure pain threshold: IG vs. CG (static touch/placebo): week 12: *p* > 0.05, week 24: *p* > 0.05
Conclusion	“Craniosacral therapy and Bowen therapy improved sleep quality, and Bowen therapy and standard exercises improved pain threshold in the short term. These improvements were retained within the groups in the long term by adding exercises. Craniosacral therapy and Bowen therapy are treatment options to improve sleep and reduce pain in fibromyalgia syndrome.”

Abbreviations: CG, control group. IG, intervention group.

## Data Availability

No new data were generated or analysed in this study. All data supporting the findings of this review are derived from previously published studies cited in the reference list. The data extraction tables underlying this review, including study characteristics and extracted outcome data (Tables S4–S21), are available as online Supplementary Materials to this article. The review protocol is publicly available via the PROSPERO registry (registration number: CRD42022330778) at https://www.crd.york.ac.uk/prospero/ (accessed on 21 May 2025).

## Supplementary Materials

The following supporting information can be downloaded at: https://www.mdpi.com/article/10.3390/healthcare14070928/s1, Table S1: Quality appraisal of the included randomised controlled trials using the ‘Cochrane Collaboration Tool 1’—study level; Table S2: Quality appraisal of the excluded randomised controlled trials using the ‘Cochrane Collaboration Tool 1’—study level; Table S3: Quality appraisal of the systematic review and meta-analysis concerning chronic non-specific low back pain using AMSTAR 2; Table S4: Overview of study characteristics and description of interventions of included studies: neck part 1; Table S5: Overview of study characteristics of included studies: neck part 2; Table S6: Overview of study characteristics and description of interventions of included studies: neck or (lower) back; Table S7: Overview of study characteristics and description of interventions of included studies: shoulder; Table S8: Overview of study characteristics and description of interventions of included studies: lower back; Table S9: Overview of study characteristics and description of interventions of included studies: knee; Table S10: Overview of study characteristics and description of interventions of included studies: foot; Table S11: Overview of study characteristics and description of interventions of included studies: osteoporosis; Table S12: Overview of study characteristics and description of interventions of included studies: fibromyalgia; Table S13: Summary of effectiveness of included studies: neck part 1; Table S14: Summary of effectiveness and description of interventions of included studies: neck part 2; Table S15: Summary of effectiveness of included studies: neck or (lower) back; Table S16: Summary of effectiveness of included studies: shoulder; Table S17: Summary of effectiveness of included studies: lower back; Table S18: Summary of effectiveness of included studies: knee; Table S19: Summary of effectiveness of included studies: foot; Table S20: Summary of effectiveness of included studies: osteoporosis; Table S21: Summary of effectiveness of included studies: fibromyalgia. References [14,16,17,18,19,20,21,22,23,24,25,26,27,28,29,30,31,32,33,34,35,36,37,38,39,40,41,42,43,44,45,46,47,48,49,50,51,52,53,54,55,56,57,58,59,60,61,62,63,64,65,66,67,68,69,70,71,72,73,74,75,76,77,78,79,80,81,82,83,84,85,86,87] also be included in the Supplementary Materials.
