# Supplementary material for: Osteopathy for Musculoskeletal Pain: A Systematic and Umbrella Review of Effectiveness and Safety
Source: Healthcare (Basel). 2026 Apr 2;14(7):928. doi: 10.3390/healthcare14070928 (PMC13072756; doi:10.3390/healthcare14070928)
Supplement: Supplementary file 1 [file healthcare-14-00928-s001.zip › healthcare-4172977-supplementary.pdf]

SUPPLEMENTARY MATERIAL

Quality appraisal of the randomised controlled trials using the ’Cochrane Collaboration Tool 1’

Table S1: Quality appraisal of the ***included*** *randomised controlled trials using the ‘Cochrane Collaboration Tool 1’ – study level*

Indication	Trial	Adequate generation of randomisation sequence	Adequate allocation concealment	Blinding		Incomplete outcome data addressed	Selective out-come reporting unlikely	No other aspects which increase the risk of bias	Risk of bias – study level
				Patient	Treating physician/ therapist[1]/outcome assessor				
Neck or (lower) back	Williams 2003 [22]	Unclear[2]	Unclear[3]	No[4]	Unclear[5]	Unclear[6]	Unclear[7]	Yes[8]	High[9]
Neck or (lower) back	Tozzi 2011 [28]	Yes	Unclear[3]	No[10]	Yes[11]	Yes	Unclear[5]	Yes[8]	High[9]
Fibromyalgia	Castro-Sanchez 2011a [15]	No[12]	Unclear[13]	No[14]	Yes[15]	Yes[16]	Unclear[5]	Yes[8]	High[17]

[1] The nature of the intervention does not always allow to blind those who deliver osteopathy. Since it is not always possible to blind a treating physician/therapist to osteopathy, we assessed the blinding of the trials with ‘yes’, i.e. low risk of bias if we judged that the lack of blinding was not affecting the results.

[2] “The unit of randomization was the patient.” Random number tables wer used and „kept secure from all participants. Using information from the referral form, she stratified the sample by symptom location, the referring GPs’ perception of symptom severity and whether the pain was a first episode or a recurrence.“

[3] Insufficient information about the allocation concealment: no description of the used method.

[4] “The intervention group was referred to the osteopathic clinic based in Llanfairfechan health centre.”

[5] Insufficient information to permit judgement of ‘Yes’ or ‘No’.

[6] Insufficient reporting of reasons for missing data.

[7] Insufficient information (no study protocol available) to permit judgement of low risk of bias.

[8] No other aspects that can increase the risk of bias have been found.

[9] Due to the lack of blinding of the patients, a high risk of bias can be assumed.

[10] Only “the Sham-Control group blindly received a sham treatment.”

[11] “Two medical doctors … were asked to compare the results independently. They were blind to the groups (Experimental and Control) from which the images were obtained.” Treating physicians were blinded, but not in all treatment measurements.

[12] “Patients were randomly assigned by means of a balanced stratified assignment to an experimental (n=47) or placebo (n=47) group.” However, random components in the sequence generation process are not adequate.

[13] “The sequences assigned to patients were placed in envelopes containing the allocation to each study group.” However, method of concealment is not described in sufficient detail to allow a definite judgement. It remains unclear whether envelopes were sequentially numbered, opaque and sealed.

[14] “The patients themselves were not blinded to their status.”

[15] “Outcomes were determined by another researcher, who was blinded to the study group of patients. However, the physiotherapist (specialist in myofascial therapy) who administered both intervention protocols” was not blinded.

[16] Reasons for missing outcome data unlikely to be related to true outcome.

[17] Due to inadequate generation of randomisation sequence and the lack of blinding of the patients, a high risk of bias can be assumed.

Indication	Trial	Adequate generation of randomisation sequence	Adequate allocation concealment	Blinding: Patient	Blinding: Treating physician/ therapist[1]/outcome assessor	Incomplete outcome data addressed	Selective out-come reporting unlikely	No other aspects which increase the risk of bias	Risk of bias – study level
Fibromyalgia	Matarán-Penarrocha 2011 [27]	Unclear[18]	Unclear[3]	Yes[19]	Yes[20]	Yes	Unclear[5]	Yes[8]	Unclear
Foot	Ajimsha 2014 [31]	Unclear[5]	Unclear[3]	Yes[21]	Yes[22]	Yes	Unclear[5]	Yes[8]	Unclear
Foot	Bac 2022 [33]	Yes[23]	Unclear[3]	Unclear[5]	Yes[24]	Yes	Yes[25]	Yes[8]	Unclear
Knee	Zago 2021 [30]	Yes[26]	Yes[27]	Unclear[5]	Yes[28]	Yes	Yes[25]	Yes[8]	Unclear
Knee	Licciardone 2004 [32]	Unclear[5]	Unclear[29]	Yes[19]	Yes[30]	Yes	Unclear[5]	Yes[8]	Unclear
Shoulder/Arm	Hunter 2022 [29]	Yes[31]	Yes[32]	No[33]	Yes[34]	Yes	Unclear[35]	Yes[8]	High[9]
Shoulder/Arm	Mishra 2018 [26]	Yes[36]	Unclear[3]	Unclear[5]	Unclear[5]	Yes	Unclear[37]	Yes[8]	Unclear

---

[18] Patients „were randomly assigned by means of a balanced stratified assignment to an intervention (n=52) or placebo (n=52) group." However, insufficient information about the sequence generation process to permit judgement of 'Yes' or 'No'.

[19] This is a double-blinded study.

[20] This is a double-blinded study. However, no information is gives if the treating physician/therapist and/or outcome assessor were blinded.

[21] This is a double-blinded study. However, no information is gives if the patients were blinded.

[22] „Both groups were treated by clinicians blinded to the group and the outcome of the study." "Two evaluators blinded to the group to which the participants belonged analyzed scores."

[23] "Qualification was based on the simple randomization (coin toss) performed by the main author."

[24] "Until the final preparation of the database, the main author was the only person who knew which group each researched person was assigned to. The therapy was performed by other therapists, and the examinations were performed by another member of the therapeutic team."

[25] Study protocol available.

[26] "Randomization was performed on the first day (www.random.org)."

[27] "The numbers were placed in a sealed and opaque envelope and drawn by the participants."

[28] "Data analysis was performed by a fourth member of the research group to maintain data blinding" "Blinding of the researchers was also performed, and each intervention was made by 2 independent researchers."

[29] "Precoded cards in sealed envelopes were used to randomly allocate patients to groups" However, method of concealment is not described in sufficient detail to allow a definite judgement. It remains unclear whether envelopes were sequentially numbered and opaque.

[30] "All study personnel who were responsible for developing OMT plans or measuring primary outcomes were blinded to group assignments. The only personnel aware of these assignments were the undergraduate fellows who performed OMT and sham treatments; however, they did not measure any of the study outcomes."

[31] "Each participant was assigned to 1 of the 3 groups by simple randomization in a 1:1:1 ratio using a computerized random number generator."

[32] „Allocation concealment was achieved by using an external individual, independent to participant recruitment and the treating practitioner, placing the generated random numbers into sequentially numbered sealed opaque envelopes."

[33] This is a single-blinded trial.

[34] "The treating practitioner was not blinded; however, when providing manual therapy a practitioner cannot be blinded to the technique they apply" But ist is also stated: "Measurements were conducted by a registered osteopath with 7 years clinical experience, blinded to group allocation."

[35] Study protocol available. However, primary/secondary outcomes and measurements are not predefined in the protocol.

[36] Patients "were divided into two groups, Group A and Group B, 30 each, through the computer randomisation."

[37] Study protocol available, however, this was retrospectively registered.

Indication	Trial	Adequate generation of randomisation sequence	Adequate allocation concealment	Blinding		Incomplete outcome data addressed	Selective out-come reporting unlikely	No other aspects which increase the risk of bias	Risk of bias – study level
				Patient	Treating physician/ therapist[1]/outcome assessor				
Neck	Capó-Juan 2017 [25]	Yes[38]	Yes[39]	Yes[40]	No[40]	Yes[41]	Unclear[42]	Yes[8]	High[43]
Neck	Haller 2016 [21]	Yes[44]	Unclear[45]	Yes[46]	Yes[47]	Yes[48]	Yes[49]	Yes[8]	Unclear[50]
Neck	Klein 2013 [24]	Yes[51]	Yes[52]	Yes[53]	Yes[53]	Yes[54]	Unclear[55]	Yes[8]	Unclear[56]
Neck	Rodrıguez-Huguet 2020 [20]	Yes[57]	Unclear[58]	No[90]	Yes[59]	Yes[54]	Yes[60]	Yes[8]	High[61]
Osteoporosis	Papa 2012 [23]	Yes[62]	Unclear[135]	No[63]	Yes[64]	Yes[65]	Unclear[55]	Yes[8]	High[9]

[38] "The patients were assigned to different groups (A, B, or C) according to an allocation number generated from a random table."
[39] "Only the Principal Physiotherapist knew the assignment group of each patient."
[40] Single blinded randomised controlled trail – "In single blind trials the recipient party (patient) is blinded."
[41] No missing data, all participants evaluated.
[42] No study protocol available.
[43] Due to the lack of blinding of the outcome acessors, a high risk of bias can be assumed.
[44] "A nonstratified allocation sequence with randomly varying block lengths using the random number generator RANUNI from the SAS/STAT software" was used.
[45] Opaque envelops sorted in the ascending order of randomisation were used. However, method of concealment is not described in sufficient detail to allow a definite judgement. It remains unclear whether envelopes were sequentially sealed.
[46] "Patients were blinded to the group allocation and to the fact that 1 group would receive sham treatment as it was recommended for manual therapy trials; instead they were told that 2 different CST techniques would be tested."
[47] "Investigators assessing outcomes remained blind to patients' group allocation during the whole study period."
[48] No missing data.
[49] Outcome measurement according to the study protocol.
[50] One domain (patient blinding) was answered with "Unclear", which is why an unclear risk of bias can be assumed.
[51] The "Research Randomizer … with variable block sizes of 8, 10 and 12 (permuted block design)" was used.
[52] Sequentially numbered, opaque, sealed envelopes were used.
[53] "With patients, the study assistant and outcome assessor (AB) blinded."
[54] No missing outcome data.
[55] NI about a study protocol.
[56] Due to the lack of a study protocol, the risk for bias cannot be classified.
[57] "Allocation was created by … using a random allocation software program."
[58] Allocation „was concealed in sequentially numbered envelopes." However, method of concealment is not described in sufficient detail to allow a definite judgement. It remains unclear whether envelopes were opaque and sealed.
[59] "Data collection was conducted by a physician who was blinded as to which participants received experimental or comparison intervention."
[60] Outcomes were reported as intended in the protocol.
[61] Due to the lack of blinding of the participants, a high risk of bias can be assumed.
[62] "Patients were randomized through a computer-generated sequence."
[63] Single-blinded trail and blinded was an investigator.
[64] "An investigator blinded to group assignment."
[65] "Twenty-four of them did not complete the study for reasons not dependent on the current study, complications of underlying conditions for 4 and 6 for difficulty in reaching the venue of the study."

Interpretation: see at the end of Table S2

*Table S2: Quality appraisal of the **excluded** randomised controlled trials using the 'Cochrane Collaboration Tool 1' – study level*

Indication	Trial	Adequate generation of randomisation sequence	Adequate allocation concealment	Blinding		Incomplete outcome data addressed	Selective outcome reporting unlikely	No other aspects which increase the risk of bias	Risk of bias – study level
				Patient	Treating physician/ therapist[66]/outcome assessor				
Fibromyalgia	Castro-Sanchez 2011b [16]	No[67]	Unclear[68]	No[69]	Yes[70]	Yes	Unclear[71]	Yes[72]	High[73]
Fibromyalgia	Castro-Sanchez 2011c [70]	No[74]	Unclear[75]	Unclear[71]	Unclear[71]	Yes	Unclear[71]	Yes[72]	High[76]
Fibromyalgia	Coste 2021 [71]	Yes	Unclear[77]	No[78]	No[79]	No[80]	No[81]	Yes[72]	High[82]
Foot	Eisenhart 2003 [35]	Unclear[71]	Unclear[77]	Unclear[71]	Unclear[71]	Yes	Unclear[71]	Yes[72]	Unclear

66 The nature of the intervention does not always allow to blind those who deliver osteopathy. Since it is not always possible to blind a treating physician/therapist to osteopathy, we assessed the blinding of the trials with 'yes', i.e. low risk of bias if we judged that the lack of blinding was not affecting the results.

67 "The final study group of 92 patients (aged 16-65 years) were assigned by a balanced stratified random assignment method to an intervention group for craniosacral therapy (n=46 females) or a placebo group for sham treatment with disconnected magnetotherapy equipment (n=46 females)." However, random components in the sequence generation process are not adequate.

68 "The sequences assigned to patients were placed in envelopes containing the allocation to each study group." However, method of concealment is not described in sufficient detail to allow a definite judgement. It remains unclear whether envelopes were sequentially numbered, opaque and sealed.

69 "Patients were not blinded to the therapy allocation although the patients were not aware that one was a sham treatment."

70 "Craniosacral and magnetotherapy therapists were not blinded to the therapy allocation." However, „pain intensity and heart rate variability were evaluated by a blinded assessor, who did not know whether patients belonged to the intervention or placebo group."

71 Insufficient information to permit judgement of 'Yes' or 'No'.

72 No other aspects that can increase the risk of bias have been found.

73 Due to inadequate generation of randomisation sequence and the lack of blinding of the patients, a high risk of bias can be assumed.

74 The authors did not state any sequence generation. However, it is to be assumed that the sequence generation occurred as in their previous studies from 2011.

75 Patients "were randomly assigned to an experimental (n=32) or placebo (n=32) group by using sealed envelopes." However, method of concealment is not described in sufficient detail to allow a definite judgement. It remains unclear whether envelopes were sequentially numbered and opaque.

76 Due to inadequate generation of randomisation sequence, a high risk of bias can be assumed.

77 Insufficient information about the allocation concealment: no description of the used method.

78 „Patients were blind to treatment assignment." However, „the differential dropout rate before the end of the first treatment session, resulting in missing-not-at-random data, suggests that blinding was not successful for some of the included subjects."

79 "The therapists were necessarily unblinded to study group assignment given their role in delivering the assigned treatment, but they were not aware of block size and variation." However, „A blind interim assessment of treatment credibility and expectancies of improvement was conducted on the first 30 patients (2 × 15) included in the trial."

80 Imbalance in numbers or reasons for missing data across groups.

81 In the study protocol the Brief Pain Inventory (BPI) was defined as a secondary outcome measurement. However, results on pain measured by the BPI were not presented in the published article.

82 Due to the lack of blinding of the patients and therapists, imbalance in numbers or reasons for missing data across groups, and selective outcome reporting, a high risk of bias can be assumed.

Indication	Trial	Adequate generation of randomisation sequence	Adequate allocation concealment	Blinding		Incomplete outcome data addressed	Selective out-come reporting unlikely	No other aspects which increase the risk of bias	Risk of bias – study level
				Patient	Treating physician/ therapist[66]/outcome assessor				
Foot	Renan-Ordine 2011 [34]	Yes[83]	Unclear[77]	Unclear[71]	Yes[84]	Unclear[85]	Unclear[71]	Yes[72]	Unclear
Shoulder/Arm	Ajimsha 2012 [72]	Unclear[71]	Unclear[77]	No[86]	Yes[87]	Yes	Unclear[71]	Yes[72]	High[88]
Shoulder/Arm	Geldschläger 2004 [73]	No[89]	Unclear[77]	No[90]	No[91]	Unclear[92]	Unclear[71]	Yes[72]	High[93]
Shoulder/Arm	Iqbal 2020 [74]	Yes[94]	Unclear[77]	Yes[86]	No[86]	Yes	Unclear[95]	Yes[72]	High[96]
Shoulder/Arm	Schwerla 2020 [75]	Yes[97]	No[98]	No[99]	No[100]	Yes	Unclear[101]	Yes[72]	High[102]
Neck	Brück 2021 [76]	Yes[103]	Unclear[104]	No[105]	Yes[106]	Yes[107]	Unclear[101]	Unclear[108]	High[88]

83 “Participants were randomly assigned to 2 groups using a table of random numbers created by on-line software (www.randomization.com).”
84 “Pressure pain thresholds (PPT) levels and SF-36 scoring were assessed by an assessor blinded to group assignment.”
85 Insufficient reporting of attrition/exclusions to permit judgement of ‚Yes‘ or ‚No‘ (number of analysed patients not stated).
86 This is a single-blinded trial.
87 “Two evaluators blinded to the group to which the participants belonged analyzed scores.” However, the „practitioners could not be blinded.“
88 Due to the lack of blinding of the patients, a high risk of bias can be assumed.
89 Sequence generated by rules based on date of admission.
90 Patients were not blinded.
91 Therapists were not blinded.
92 No reasons for missing outcome data stated.
93 Due to inadequate generation of randomisation sequence, the lack of blinding of the patients and therapists, a high risk of bias can be assumed.
94 “The subjects were randomly allocated into two equal groups using the sealed envelope method.”
95 “The current RCT was not registered with the relevant registry due to the unavailability of trial registry in the country and the institution at the time.”
96 Due to lack of blinding of the therapists and outcme assessors, a high risk of bias can be assumed.
97 “A computer-generated randomization list with variable block lengths of 4-8 was held.”
98 “Participants’ allocation to the respective groups was revealed only after date of birth and initials had been conveyed by telephone, and documented in the original randomization list.”
99 No patient blinding.
100 No evaluator blinding.
101 Study protocol available, however, this was retrospectively registered.
102 Due to inadequate allocation concealment, the lack of blinding of the patients and therapists, a high risk of bias can be assumed.
103 “Patients were randomly allocated by drawing lots.”
104 “20 sealed envelopes were in one box. For each subject, one envelope was drawn by a blinded assessor.” However, method of concealment is not described in sufficient detail to allow a definite judgement. It remains unclear whether envelopes were sequentially numbered and opaque.
105 “The participants could not be blinded.”
106 “All data were collected by the practice staff who were not involved in the intervention and were not informed about group allocation”, “therapists could not be blinded to the intervention.”
107 No missing data.
108 The study protocol mentions 4 measurement points, but only 2 are mentioned in the study.

Indication	Trial	Adequate generation of randomisation sequence	Adequate allocation concealment	Blinding: Patient	Blinding: Treating physician/ therapist[66]/outcome assessor	Incomplete outcome data addressed	Selective out-come reporting unlikely	No other aspects which increase the risk of bias	Risk of bias – study level
Neck	Cholewicki 2021 [53]	Yes[109]	Yes[110]	No[90]	Yes[111]	Yes[112]	No[113]	Yes[72]	High[88]
Neck	El-Gendy 2019 [77]	Yes[114]	Yes[115]	No[116]	No[117]	Yes[118]	Unclear[119]	Yes[72]	High[120]
Neck	Groisman 2020 [78]	Yes[121]	Yes[122]	No[123]	Yes[124]	Yes[125]	No[126]	Yes[72]	High[127]
Neck	Leaver 2010 [79]	No[128]	Yes[129]	No[130]	Yes[131]	Yes[132]	Yes[133]	Yes[72]	High[88]

109 "Randomization module in REDCap was used to assign participants to group allocation."
110 "The allocation table was generated by a computer … no way to predict any participant's allocation before enrolment."
111 "The PI, statistician, and treating team physicians were all blinded to group assignment (i.e. OMT or waiting period)."
112 Information about missing data is given.
113 Change in medication was intended to be evaluated in the protocol but no information (NI) about that was given in the study.
114 "Patients were randomly assigned into 3 equal groups … with the use of a computer-based randomization program."
115 "Patients were blinded about which group they were allocated by an independent researcher."
116 Authors did not state that patients were blinded.
117 Authors did not state that assessors or treating physicians/therapists were blinded.
118 No missing data, no dropouts.
119 No study protocol available.
120 Due to the unclear information regarding the blinding and the lack of a study protocol, the risk of bias is high.
121 "An online software … was used to generate a randomization list, and 90 participants were allocated into two treatments groups."
122 "Generated numbers were placed in 90 sealed opaque envelopes…only opened after the participant had completed all the baseline assessments."
123 "All the participants were told about the existence of the EG and OMT/EG groups."
124 "The evaluators who carried out the assessments were blinded in relation to the group that each participant belonged"; "The therapists who performed the treatments could not be blinded."
125 "Test considers the missing data allowing for intent-to-treat analysis. Effects on time, group and time-by-group interaction were considered."
126 Other outcome measurements used as intended in the protocol.
127 Due to the lack of blinding of the patients and deviations from the protocol, a high risk of bias can be assumed.
128 "Randomization occurred at the point in the course of treatment at which the treating practitioner chose to introduce manipulation."
129 Numbered sealed opaque envelopes were used.
130 "It was not possible to blind the participants or practitioners to treatment allocation because of the nature of the interventions."
131 "Data collection and analysis were conducted by researchers who were blind to treatment allocation"; "It was not possible to blind … practitioners to treatment allocation."
132 Missing outcome data balanced in numbers across intervention groups, with similar reasons for missing data across groups.
133 Outcome data reported according to the study protocol.

Indication	Trial	Adequate generation of randomisation sequence	Adequate allocation concealment	Blinding		Incomplete outcome data addressed	Selective outcome reporting unlikely	No other aspects which increase the risk of bias	Risk of bias – study level
				Patient	Treating physician/ therapist[66]/outcome assessor				
Neck	Martínez-Segura 2006 [80]	Yes[134]	Unclear[135]	No[136]	Yes[137]	Yes[138]	Unclear[139]	Yes[72]	High[88]
Neck	McReynolds 2005 [81]	Yes[140]	Yes[141]	No[142]	Unclear[143]	Yes[138]	Unclear[139]	Yes[72]	High[88]
Neck	Osama 2021 [82]	Yes[144]	Unclear[145]	No[90]	Yes[146]	Yes[132]	No[147]	Yes[72]	High[148]
Neck	Rezkallah 2018 [83]	Yes[149]	Unclear[150]	Yes[151]	No[151]	Yes[132]	Unclear[139]	Yes[72]	High[152]
Neck	Rodríguez-Fuentes 2016 [84]	Unclear[153]	Unclear[135]	Yes[132]	No[151]	Yes[138]	Unclear[139]	Yes[72]	High[152]

134 "Were divided randomly into 2 groups using a table of random numbers."

135 NI about adequate allocation concealment.

136 The authors stated that they„ cannot say that subjects were truly blinded because patients could know that they had been allocated to receive high velocity-low amplitude thrust (joint cavitation) or control mobilization procedure (nontissue tension)."

137 "Outcomes were assessed by an examiner blinded to the treatment allocation of the subject."

138 No missing outcome data.

139 NI about a study protocol.

140 "Enrolling physicians randomly assigned patients to receive either OMT or IM ketorolac using a predetermined random number table."

141 "The treatment arm was not disclosed to patients until after informed consent was obtained."

142 "Attempts were not made to blind patients or physicians as to which treatment was being given at the time of treatment."

143 NI if outcome assessors were blinded.

144 "Randomly allocated via block randomization to the three treatment groups."

145 Sealed envelopes were used. However, method of concealment is not described in sufficient detail to allow a definite judgement. It remains unclear whether envelopes were sequentially numbered and opaque. However, it is not stated that they were numbered and opaque.

146 "It was a single blind study with the assessor being blind."

147 Cervical Range of Motion (CROM) and Neck Disability Index (NDI) not evaluated as written in the study protocol.

148 Due to the lack of blinding of the patients the deviations from the study protocol, a high risk of bias can be assumed.

149 "Randomization was implemented simply by means of a computer-generated randomized table using the SPSS programme."

150 "Individual and sequentially numbered index cards were secured in opaque envelopes." However, method of concealment is not described in sufficient detail to allow a definite judgement. It remains unclear whether envelopes were sealed.

151 Single blinded randomised controlled trail – "In single blind trials the recipient party (patient) is blinded."

152 Due to the lack of blinding of the outcome assessors, a high risk of bias can be assumed.

153 "Patients … were randomly distributed into two groups according to two therapeutic intervention programs" – NI about randomisation process.

Indication	Trial	Adequate generation of randomisation sequence	Adequate allocation concealment	Blinding: Patient	Blinding: Treating physician/ therapist[66]/outcome assessor	Incomplete outcome data addressed	Selective out-come reporting unlikely	No other aspects which increase the risk of bias	Risk of bias – study level
Neck	Rotter 2020 [85]	Yes[154]	Yes[155]	No[156]	No[157]	Yes[158]	Yes[159]	Yes[72]	High[160]

Interpretation:

Low risk of bias: Plausible bias unlikely to seriously alter the results. Low risk of bias for all key domains.

Unclear risk of bias: Plausible bias that raises some doubt about the results. Unclear risk of bias for one or more key domains.

High risk of bias: Plausible bias that seriously weakens confidence in the results. High risk of bias for one or more key domains.

[154] "Patients were randomized to one of the two treatment groups (1:1 ratio) by a computer-generated block randomization process in the study center with variable block length."

[155] "The allocation was performed in the study center by a study nurse and was concealed." The allocation was performed in the study center by a study nurse and was concealed.

[156] "The blinding of patients or the therapist with regard to group allocation was not feasible."

[157] "Blinding of outcome assessors (patients) was not feasible…"; "…the blinding of patients or the therapist with regard to group allocation was not feasible."

[158] Reasons for missing outcome data unlikely to be related to true outcome.

[159] Outcomes reported according to study protocol.

[160] Due to the lack of blinding of the patients and outcome assessors, a high risk of bias can be assumed.

Quality appraisal of the systematic review and meta-analysis concerning chronic non-specific low back pain using AMSTAR 2

Table S3: Quality appraisal of the systematic review and meta-analysis concerning chronic non-specific low back pain using AMSTAR 2

Dal Farra et al., 2021 [14]	Reviewers (LG, VH)
1. Did the research questions and inclusion criteria for the review include the components of PICO?	Yes
2. Did the report of the review contain an explicit statement that the review methods were established prior to the conduct of the review, and did the report justify any significant deviations from the protocol?	Yes
3. Did the review authors explain their selection of the study designs for inclusion in the review?	Yes
4. Did the review authors use a comprehensive literature search strategy?	Yes
5. Did the review authors perform study selection in duplicate?	Yes
6. Did the review authors perform data extraction in duplicate?	Yes
7. Did the review authors provide a list of excluded studies and justify the exclusions?	Partial Yes[161]
8. Did the review authors describe the included studies in adequate detail?	Yes
9. Did the review authors use a satisfactory technique for assessing the risk of bias (RoB) in individual studies included in the review?	Yes
10. Did the review authors report on the sources of funding for the studies included in the review?	No[162]
11. If meta-analysis was performed, did the review authors use appropriate methods for statistical combination of results?	Yes
12. If meta-analysis was performed, did the review authors assess the potential impact of RoB in individual studies on the results of the meta-analysis or other evidence synthesis?	Yes
13. Did the review authors account for RoB in individual studies when interpreting/discussing the results of the review?	Yes
14. Did the review authors provide a satisfactory explanation for and discussion of any heterogeneity observed in the results of the review?	Yes
15. If they performed quantitative synthesis, did the review authors carry out an adequate investigation of publication bias (small study bias) and discuss its likely impact on the results of the review?	Yes
16. Did the review authors report any potential sources of conflict of interest, including any funding they received for conducting the review?	Yes
Overall Confidence	High

Reasoning
No or one non-critical weakness: the systematic review provides an accurate and comprehensive summary of the results of the available studies that address the question of interest.

[161] Authors provided the number of excluded studies and exclusion reasons, but not a list of references of excluded studies.

[162] The authors did not provide information regarding the sources of funding for the studies included in the review.

Extraction tables

Overview of study characteristics of included studies

Table S4: Overview of study characteristics and description of interventions of included studies: neck part 1

Author, year [reference]	Haller 2016 [21]	Klein 2013 [24]
Indication	Neck pain	Neck pain
Acute vs chronic	Chronic	Acute
Country (corresponding author)	Germany	Germany
Study design	Double-blind RCT	Double-blind RCT
Number of randomised patients (age mean (SD))	54 (81.5% female; 44.6 ±10.0)	61 (45 female; IG: 47.9 (10.1); CG: 41.9 (10.4))
Dropout rate	Lost to assessment at week 8: 3<br>Lost to assessment at week 20: 9	0
Intervention/technique	Craniosacral therapy	Strain—counterstrain treatment
Intervention applied by (profession)	Licensed physiotherapists with advanced craniosacral therapy qualification, and on average 6 years of clinical practice	General practitioner with additional qualifications in sports medicine, manual therapies and completed full osteopathic curriculum (postgraduate) with 8 years of experience in using osteopathic treatments
Comparison	Light-touch sham treatment	Sham treatment[163]
Total number of sessions	8	1
Treatment period	8 weeks	1 session
Duration of each session	45 min	NR
Frequency of treatment	1x/week	NR
Follow-up assessment	3 months after treatment	None

Abbreviations: CG, control group. IG, intervention group. min, minutes. NR, not reported. RCT, randomised controlled trial. SD, standard deviation.

Table S5: Overview of study characteristics of included studies: neck part 2

Author, year [reference]	Capó-Juan 2017 [25]	Rodríguez-Huguet 2020 [20]
Indication	Cervical myofascial pain	Neck pain
Acute vs chronic	NR	Subacute-chronic
Country (corresponding author)	Spain	Spain
Study design	3-arm, experimental, single-blind RCT	Single-blind RCT
Number of randomised patients (age range)	75 (60 female; 20-55)	54 (26 female; inclusion criteria: 20-60)
Dropout rate	0	0
Intervention/technique	Pressure release	Myofascial release
Intervention applied by (profession)	NR	Therapist with 9 years of experience in myofascial release therapy technique and certificate
Comparison	Kinesiotaping; placebo[164]	Standard physical therapy[165]
Total number of sessions	1	5
Treatment period	1 session	2 weeks
Duration of each session	NR	45 min
Frequency of treatment	1x	2.5x/week

[163] „The finger of the therapist was placed at the height of C4 paravertebraly on the right hand side of the dorsal part and the head was rotated by 30° to the left to basic position without any flexion, extension or lateral flexion. This position was also held for 90 s. Afterwards, a slow reposition to basic position was carried out."

[164] algometric bilateral pressure

[165] massage, ultrasound therapy, and transcutaneous electric nerve stimulation

Author, year [reference]	Capó-Juan 2017 [25]	Rodríguez-Huguet 2020 [20]
Follow-up assessment	None	1 month

Abbreviations: Min, minutes. NR, not reported.

Table S6: Overview of study characteristics and description of interventions of included studies: neck or (lower) back

Author, year [reference]	Tozzi 2011 [28]	Williams 2003 [22]
Indication	Neck or low back pain	Neck or back pain
Acute vs chronic	Acute/Chronic	(Sub)acute
Country (corresponding author)	Italy	UK
Study design	RCT	Pragmatic RCT
Number of randomised patients (age range)	120 (IG: 18 female; 21-58; CG: 18 female; 18-56)	201 (female: NR; 16-65[166])
Dropout rate	NR	18[167]
Intervention/technique	Fascial release	Osteopathic spinal manipulation
Intervention applied by (profession)	Osteopath (5 years experience)	General practitioner registered as osteopath
Comparison	Sham treatment	Usual care
Total number of sessions	1	3-4
Treatment period	1 session	2 months
Duration of each session	4-8 min	NR
Frequency of treatment	NR	0.5-1x/week
Follow-up assessment	None	6 months

Abbreviations: CG, control group. IG, intervention group. NR, not reported. RCT, randomised controlled trial. UK, United Kingdom.

Table S7: Overview of study characteristics and description of interventions of included studies: shoulder

Author, year [reference]	Mishra 2018 [26]	Hunter 2022 [29]
Indication	Upper trapezius spasm	Shoulder impingement syndrome
Acute vs chronic	NR	NR
Country (corresponding author)	India	Australia
Study design	RCT	3-arm single-blind RCT
Number of randomised patients (age range or mean ± SD)	60 (31 female; 20-55)	75 (25 per group) (IG: 10 female; 62.0 ± 9.6; placebo group: 9 female; 61.4 ± 11.3; muscle energy technique + soft tissue massage group: 9 female; 56.9 ± 9.2)
Dropout rate	0	18 (until last FU)
Intervention/technique	Myofascial release	Muscle energy technique
Intervention applied by (profession)	Physiotherapists	Osteopath (14 years clinical experience)
Comparison	Active release technique	Muscle energy technique + soft tissue massage; placebo
Total number of sessions	NR	4
Treatment period	7 days	4 weeks (test point week 3)[168]
Duration of each session	NR	15 min
Frequency of treatment	NR	1x/week

[166] target population

[167] IG: 70 patients (76%) returned 2 month questionnaire. 63 patients (70%) returned 6 month questionnaire. Data from medical records: 86 patients (95%). CG: 72 patients (66%) returned 2 month questionnaire. 72 patients (66%) returned 6 month questionnaire. Data from medical records: 101 patients (93%).

[168] First test point at week 3 (= discharge) → discrepancy in article was found as the treatment period was 4 weeks

Author, year [reference]	Mishra 2018 [26]	Hunter 2022 [29]
Follow-up assessment	None	4 weeks after discharge (test point week 7), 6 months (test point week 29), 1 year (test point week 55)

Abbreviations: FU, follow-up. min, minutes. NR, not reported. RCT, randomised controlled trial.

Table S8: Overview of study characteristics and description of interventions of included studies: lower back

Author, year [reference]	Dal Farra, 2021 [14]
Indication	Low back pain
Acute vs chronic	Chronic
Country (corresponding author)	Italy
Study design	Systematic review and meta-analysis
Included study design	RCTs
Number of included studies	10
Number of included patients (age mean (SD))	1,160 (female: NR; mean age 43.3 +/- 7.7)
Dropout rate	Range: 0–77%
Intervention/technique	Osteopathic interventions, i.e. OMT (n=6), myofascial release (n=2), craniosacral treatment (n=1) and osteopathic visceral manipulation (n=1)
Intervention applied by (profession)	NR
Comparison	No active treatment (sham therapy or no intervention; n=5), active treatment (standard exercise, classic massage; n=5)
Total number of sessions	Range 1-24, mean 8.7 +/- 5.8
Treatment period	Ranged 2-24 weeks, mean 9.9 +/- 7.04
Duration of each session	15-60 min, mode: 45 min
Frequency of treatment	2x/week to1x/month
Follow-up assessment	4-24 weeks (in 6/10 studies)

Abbreviations: Min, minute. NR, not reported. OMT, osteopathic manipulative treatment.

Table S9: Overview of study characteristics and description of interventions of included studies: knee

Author, year [reference]	Zago 2021 [30]	Licciardone 2004 [32]
Indication	Patellofemoral pain syndrome	Knee or hip osteoarthritis, or hip fracture
Acute vs chronic	Chronic	Acute (postoperative)
Country (corresponding author)	Brazil	USA
Study design	3-arm RCT	Double-blind RCT
Number of randomised patients (age range)	82 (48 female; 18-35)	60 (42 female; 69.2 (10.3))
Dropout rate	Withdrawal from eligible patients: 5	Loss to 4-week postdischarge FU: 8
Intervention/technique	OMT	One or a combination of: myofascial release, strain—counterstrain, muscle energy, soft tissue, high-velocity low-amplitude (not at the surgical site), or craniosacral manipulation
Intervention applied by (profession)	Osteopath (8 years of experience)	Medical students (undergraduate fellows still in the training process; Department of Osteopathic Manipulative Medicine)
Comparison	Exercise programme; waiting list	Sham treatment (range-of-motion activities, light touch)
Total number of sessions	6	5.4

Author, year [reference]	Zago 2021 [30]	Licciardone 2004 [32]
Treatment period	3 weeks	NR
Duration of each session	40 min	10-30 min
Frequency of treatment	2x/week	2.4x/week
Follow-up assessment	30 days	4 weeks after discharge (only SF-36)

Abbreviations: Min, minutes. NR, not reported. OMT, osteopathic manipulative treatment. RCT, randomised controlled trial. USA, United States of America.

Table S10: Overview of study characteristics and description of interventions of included studies: foot

Author, year [reference]	Bac 2022 [33]	Ajimsha 2014 [31]
Indication	Flat foot with foot pain	Unilateral plantar heel pain
Acute vs chronic	NR	NR
Country (corresponding author)	Poland	Qatar
Study design	4-arm RCT	Double-blind RCT
Number of randomised patients (age range or mean ± SD)	70 (47 female after dropout; 20–49)	66 (49 female; IG: 42.4 ± 4.6; CG: 40.8 ± 7.1)
Dropout rate	10	1
Intervention/technique	Myofascial release	Myofascial release
Intervention applied by (profession)	Therapist	Physiotherapists certified in myofascial release (trained for min. 100 h, median experience of 12 months)
Comparison	Exercise programme; myofascial release and exercise programme[169]; no intervention	Sham ultrasound therapy
Total number of sessions	8	12
Treatment period	4 weeks	4 weeks
Duration of each session	40 min	30 min
Frequency of treatment	2x/week	3x/week
Follow-up assessment	None	12 weeks after randomisation

Abbreviations: CG, control group. h, hours. IG, intervention group. min., minimum. min, minutes. NR, not reported. RCT, randomised controlled trial. SD, standard deviation.

Table S11: Overview of study characteristics and description of interventions of included studies: osteoporosis

Author, year [reference]	Papa 2012 [23]
Indication	Osteoporosis
Acute vs chronic	NR
Country (corresponding author)	Italy
Study design	Single-blind RCT
Number of randomised patients (age mean (SD))	72 (51 female; IG: 77.2 (5.3); CG: 76.8 (8.2))
Dropout rate	0
Intervention/technique	OMT
Intervention applied by (profession)	Osteopath
Comparison	Sham manipulative treatment
Total number of sessions	6
Treatment period	6 weeks

[169] The control group 'myofascial release and exercise programme' was not compared in this report because it includes an osteopathic technique.

Author, year [reference]	Papa 2012 [23]
Duration of each session	30 min
Frequency of treatment	1x/week
Follow-up assessment	None

Abbreviations: OMT, osteopathic manipulative treatment. RCT, randomised controlled trial.

Table S12: Overview of study characteristics and description of interventions of included studies: fibromyalgia

Author, year [reference]	Matarán-Penarrocha 2011 [27]	Castro-Sanchez 2011 [15]
Indication	Fibromyalgia	Fibromyalgia
Acute vs chronic	Chronic	Chronic
Country (corresponding author)	Spain	Spain
Study design	Double-blind longitudinal clinical RCT	Single-blind RCT
Number of randomised patients (age range; mean (SD))	Randomised: 104<br>Analysed: 84 (81 females; range 34–63; mean 49.08 ± 14.17)	94 (female: NR; range 45-65; mean 54.4)
Dropout rate	20	8
Intervention/technique	Craniosacral therapy	Myofascial release
Intervention applied by (profession)	Expert craniosacral therapist	Physiotherapist (specialist in myofascial therapy)
Comparison	Placebo (simulated treatment with disconnected ultrasound)	Sham short-wave and ultrasound electrotherapy
Total number of sessions	50	10
Treatment period	25 weeks	20 weeks
Duration of each session	1 h	1 h
Frequency of treatment	2x/week	2x/week
Follow-up assessment	6 months, 1-year post-treatment	6 months, 1-year post-treatment

Abbreviations: H, hour. NR, not reported. RCT, randomised controlled trial.

Summary of effectiveness and description of interventions of included studies

Table S13: Summary of effectiveness of included studies: neck part 1

Author, year [reference]		Haller 2016 [21]	Klein 2013 [24]
Indication		Neck pain	Neck pain
Acute vs chronic		Chronic	Acute
Intervention/technique		Craniosacral therapy	Strain—counterstrain treatment
Comparison		Light-touch sham treatment	Sham treatment
Number of randomised patients (age mean (SD))		54 (81.5% female; 44.6 ±10.0)	61 (45 female; IG: 47.9 (10.1); CG: 41.9 (10.4))
Outcomes (measurements)		Pain intensity (VAS), pain on movement (POM), point of max. pain (PPT), musculus levator scapulae (PPT), musculus trapezius (PPT), musculus semispinalis capitis (PPT), functional disability (NDI), physical QoL (SF-12), physical well-being (FEW), mental QoL (SF-12), anxiety (HADS), depression (HADS), stress perception (PSQ), pain acceptance (ERDA),<br>body awareness (SBC), body dissociation (SBC), global improvement (PGI-I)	Mobility restriction (CROM),<br>pain intensity (NPDS)
Effectiveness outcomes	Absolute effects (95% CI or mean (SD); p-value of overall effect)	*Between-group difference* (95% CI; p-value):<br>Pain:<br>*Pain intensity: week 8:* -21.0 (-32.6 to -9.4); p=0.001; *week 20:* -16.8 (-27.5 to -6.1); p=0.003<br>*Pain on movement: week 8:* -18.6 (-29.2 to -8.0); p=0.001; *week 20:* -11.4 (-20.9 to -1.9); p=0.020<br>*Point of max. pain: week 8:* 50.3 (2.8 to 97.7); p=0.038; *week 20:* 23.9 (-9.9 to 57.3); n.s.<br>Pressure pain sensitivity:<br>*Musculus levator scapulae: week 8:* 34.2 (-2.9 to 71.3); n.s.; *week 20:* 10.4 (-25.1 to 45.8); n.s.<br>*Musculus trapezius: week 8:* 31.8 (1.2 to 62.4); p=0.042; *week 20:* 4.4 (-28.9 to 37.7); n.s.<br>*Musculus semispinalis capitis: week 8:* 5.8 (-19.2 to 30.8); n.s.; *week 20:* 15.1 (-12.4 to 42.5); n.s.<br>Physical health:<br>*Functional disability: week 8:* -8.2 (-14.4 to -2.1); p=0.010; *week 20:* -6.5 (-11.1 to -2.0); p=0.006<br>*Physical QoL: week 8:* 5.8 (1.3 to 10.4); p=0.013; *week 20:* 5.9 (2.8 to 9.1); p=0.000<br>*Physical well-being: week 8:* 0.2 (-0.2 to 0.5); n.s.; *week 20:* 0.2 (-0.1 to 0.7); n.s.<br>Mental health:<br>*Mental QoL: week 8:* 3.5 (-1.6 to 8.5); n.s.; *week 20:* 2.7 (-3.2 to 8.6); n.s.<br>*Anxiety: week 8:* -1.0 (-2.8 to 0.9); n.s.; *week 20:* -2.1 (-3.8 to -0.3); p=0.020<br>*Depression: week 8:* -0.7 (-2.2 to 0.8); n.s.; *week 20:* -1.9 (-3.9 to 0.2); n.s.<br>*Stress perception: week 8:* -0.4 (-8.2 to 7.4); n.s.; *week 20:* -6.4 (-15.5 to 2.8); n.s.<br>*Pain acceptance:* week 8: 0.1 (-0.2 to 0.4); n.s.; week 20: 0.2 (-0.1 to 0.4); n.s.	*Within-group differences* (mean (SD); p-value):<br>Pain intensity:<br>*baseline – after intervention 1:*<br>IG: 0.7 (0.7); p<0.001<br>CG: 0.3 (0.9); n.s.<br>Mobility restriction:<br>*baseline – after intervention 1:*<br>IG: 2.0 (6.9); n.s.<br>CG: 0.5 (5.7); n.s.<br>*Comparison between groups after intervention 1* (mean (SD); p-value):<br>Pain intensity: n.s.<br>Mobility restriction: n.s.

Author, year [reference]		Haller 2016 [21]	Klein 2013 [24]
		Body awareness:<br>*Body awareness: week 8:* 0.3 (0.1 to 0.5); p=0.001; *week 20:* 0.1 (-0.1 to 0.4); n.s.<br>*Body dissociation: week 8:* 0.9 (-0.1 to 0.4); n.s.; *week 20:* 0 (-0.3 to 0.3); n.s.<br>Global improvement:<br>*Global improvement: week 8:* -1.0 (-1.5 to -0.5); p=0.000; *week 20:* -0.7 (-1.3 to -0.1); p=0.029<br>*Intra-group pre-post difference of the IG* (mean (SD)):<br>Pain intensity:<br>Baseline: 64.1 (12.8); *week 8:* 31.7 (20.7); *week 20:* 31.6 (19.0)	
	Relative effects (95% CI; p-value of overall effect)	NR	NR
Safety outcomes	Adverse events: Relative effects (95% CI)	Serious adverse events: none<br>Minor adverse events during or subsequent to the treatment: n=6 (increased neck pain,<br>pain in the jaw area, shivering, tiredness, strong emotional reactions, weeping)	Mild transient adverse effects (n=4; pain apart from one verum patient reporting dizziness)
	Side effects	NR	NR
Conclusion		Craniosacral therapy was effective and safe in reducing neck pain intensity and may improve functional disability and the QoL up to 3 months after intervention.	Strain—counterstrain as a single intervention did not have immediate effects on mobility and pain over sham treatment.

Abbreviations: CG, control group. CI, confidence interval. CROM, Cervical Range of Motion. ERDA, Emotional/Rational Disease Acceptance Questionnaire. FEW, Questionnaire for Assessing Subjective Physical Well-being. HADS, Hospital Anxiety and Depression Scale. IG, intervention group. n.s., not significant. NDI, Neck Disability Index scale. NPDS, Neck Pain and Disability Scale. NR, not reported. PGI-I, Patients' Global Impression of Improvement. POM, Pain on Movement Questionnaire. PPT, pressure pain thresholds. PSQ, Perceived Stress Questionnaire. QoL, quality of life. SBC, Scale of Body Connection. SD, standard deviation. SF-12, 12-item Short Form Health Survey. VAS, Visual Analogue Scale.

Table S14: Summary of effectiveness and description of interventions of included studies: neck part 2

Author, year [reference]	Capó-Juan 2017 [25]	Rodríguez-Huguet 2020 [20]
Indication	Cervical myofascial pain	Neck pain
Acute vs chronic	NR	Subacute-chronic
Intervention/technique	Pressure release	Myofascial release
Comparison	Kinesiotaping; placebo[170]	Standard physical therapy[171]
Number of randomised patients (age range)	75 (20-55)	54 (26 female; inclusion criteria: 20-60)

[170] algometric bilateral pressure

[171] massage, ultrasound therapy, and transcutaneous electric nerve stimulation

Author, year [reference]		Capó-Juan 2017 [25]	Rodríguez-Huguet 2020 [20]
Outcomes (measurements)		Level of pain (subjective pain) (NPS), myofascial trigger points of sternocleidomastoid muscle (objective pain) (algometry), cervical joint range (objective pain) (goniometry), QoL (SF-12)	Pain intensity (NPRS), cervical active ROM (flexion, extension, side bending, rotation) (goniometer), pressure pain thresholds[172] (pressure algometer)
Effectiveness outcomes	Absolute effects (mean (SD); p-value of overall effect or mean difference; 95% CI; p-value; effect size)	*Pre-post differences* (mean (SD); p-value):<br>Level of pain (subjective pain):<br>IG: pre: 5.36 (0.37), post: 4.24 (0.38); p<0.001;<br>Placebo: pre: 5.04 (0.48), post: 4.40 (0.41); p>0.05;<br>Kinesiotaping: pre: 5.32 (0.42), post: 2.92 (0.52); p<0.001;<br>Myofascial trigger points of sternocleidomastoid muscle (objective pain):<br>*Sternocleidomastoid right:*<br>IG: pre: 1.76 (0.24), post: 3.12 (0.28); p<0.001;<br>Placebo: pre: 1.80 (0.27), post: 1.68 (0.33); p>0.05;<br>Kinesiotaping: pre: 1.88 (0.26), post: 4.72 (0.32); p<0.001;	*Between-group differences* (mean difference; 95% CI; p-value; effect size):<br>*2 weeks:*<br>**Pain intensity:**<br>-1.04; -1.71 to -0.36; **p<0.01**; medium<br>**Cervical active ROM:**<br>*Flexion:* -0.85; -5.28 to 3.57; n.s.; negligible<br>*Extension:* -1.15; -5.74 to 3.45; n.s.; negligible<br>*Side bending left:* 0.52; -3.09 to 4.13; n.s.; negligible<br>*Side bending right:* -0.52; -4.36 to 3.33; n.s.; negligible
Effectiveness outcomes *(continuation)*	Absolute effects (mean (SD); p-value of overall effect or mean difference; 95% CI; p-value; effect size)<br>*(continuation)*	*Sternocleidomastoid left:*<br>IG: pre: 2.08 (0.23), post: 3.52 (0.33); p<0.001;<br>Placebo: pre: 1.80 (0.28), post: 1.28 (0.28); p>0.05;<br>Kinesiotaping: pre: 2.12 (0.27), post: 4.28 (0.31); p<0.001;<br>Cervical joint range (objective pain):<br>*Flexion:*<br>IG: pre: 40.00 (1.77), post: 44.00 (1.29); p<0.001;<br>Placebo: 38.60 (1.81), post: 38.60 (1.17); p>0.05<br>Kinesiotaping: pre: 34.20 (1.59), post: 44.00 (1.82); p<0.001;<br>*Extension:*<br>IG: pre: 43.60 (1.51):, post: 45.80 (1.46); p<0.05;<br>Placebo: pre: 40.60 (1.66), post: 40.00 (1.63); p>0.05;<br>Kinesiotaping: pre: 35.80 (1.31), post: 46.20 (1.05); p<0.001;<br>*Right rotation:*<br>IG: pre: 55.00 (1.91), post: 56.60 (1.84); p>0.05;<br>Placebo: pre: 55.00 (1.47), post: 54.40 (1.64); p>0.05;<br>Kinesiotaping: pre: 46.00 (1.89), post: 61.20 (1.20); p<0.001;<br>*Left rotation:*<br>IG: pre: 54.20 (1.79), post: 56.80 (1.95); p>0.05;<br>Placebo: pre: 55.20 (1.51), post: 54.80 (1.71); p>0.05;<br>Kinesiotaping: pre: 47.80 (1.98), post: 63.20 (1.11); p<0.001;<br>QoL:<br>IG: pre: 24.36 (1.93), post: 19.32 (1.62); p<0.05;<br>Placebo: pre: 24.40 (2), post: 22.20 (1.74); p<0.05;<br>Kinesiotaping: pre: 25 (2.18), post: 14.68 (2.36); p<0.001<br>*Between-group differences at 3. appointment (p-value):*	*Rotation left:* 3.81; 0.13 to 7.50; **p<0.05**; medium<br>*Rotation right:* 3.37; -0.27 to 7.01; n.s.; small<br>**Pressure pain thresholds:**<br>*Suboccipita left:* 0.21; 0.03 to 0.40; **p<0.05**; medium<br>*Suboccipita right:* 0.32; 0.12 to 0.51; **p<0.01**; large<br>*Thoracic left:* 0.14; -0.09 to 0.37; n.s.; small<br>*Thoracic right:* 0.31; 0.06 to 0.56; **p<0.05**; medium<br>*1 month:*<br>**Pain intensity:**<br>-1.56; -2.30 to -0.81; **p<0.001**; large<br>**Cervical active ROM:**<br>*Flexion:* 2.26; -2.96 to 7.48; n.s.; negligible<br>*Extension:* 0.96; -5.36 to 7.28; n.s.; negligible<br>*Side bending left:* 4.52; -0.67 to 9.70; n.s.; small<br>*Side bending right:* 2.52; -1.85 to 6.88; n.s.; small<br>*Rotation left:* 7.37; 2.92 to 11.82; **p<0.01**; medium<br>*Rotation right:* 5.15; 0.68 to 9.61; **p<0.05**; medium<br>**Pressure pain thresholds:**<br>*Suboccipita left:* 0.34; 0.08 to 0.61; **p<0.05**; medium<br>*Suboccipita right:* 0.29; 0.04 to 0.54; **p<0.05**; medium<br>*Thoracic left:* 0.29; -0.02 to 0.59; n.s.; small<br>*Thoracic right:* 0.35; 0.03 to 0.66; **p<0.05**; medium<br>*Change score of the IG* (mean (SD)):<br>**Pain intensity:**<br>2 weeks: -4.82 (0.88); 1-month: -6.00 (1.07)

[172] i.e. „minimal amount of pressure necessary to evoke pain or discomfort at the trigger point“

Author, year [reference]		Capó-Juan 2017 [25]	Rodríguez-Huguet 2020 [20]
		Level of pain (subjective pain):<br>Pressure release vs placebo: n.s.<br>Pressure release vs kinesiotaping: p<0.05<br>Myofascial trigger points of sternocleidomastoid muscle (objective pain):<br>*Sternocleidomastoid right:*<br>Pressure release vs placebo: p<0.05<br>Pressure release vs kinesiotaping: n.s.<br>*Sternocleidomastoid left:*<br>Pressure release vs placebo: p<0.001<br>Pressure release vs kinesiotaping: n.s.<br>Cervical joint range (objective pain):<br>Pressure release vs placebo: n.s.<br>Pressure release vs kinesiotaping: n.s.<br>QoL:<br>Pressure release vs placebo: n.s.<br>Pressure release vs kinesiotaping: n.s.	
	Relative effects (95% CI; p-value of overall effect)	NR	NR
Safety outcomes	Adverse events: Relative effects (95% CI)	NR	None
	Side effects	NR	NR
Conclusion		Kinesiotaping and pressure release are two therapeutic techniques which help to reduce pain, show increased levels in Goniometry (cervical movements) and contribute to improve QoL. It seems that kinesiotaping could be more effective than pressure release.	Myofascial release therapy could be better than a standard physical therapy program for improving pain and suboccipital pressure pain thresholds in patients with neck pain. However, the difference between both treatments is less than the minimum detectable change of the numerical pain rating scale.

Abbreviations: IG, intervention group. NPRS, Numerical pain rating scale. NPS, Numerical Pain Scale. ROM, range of motion. QoL, quality of life. SF-12, 12-Item Short Form Survey.

Table S15: Summary of effectiveness of included studies: neck or (lower) back

Author, year [reference]	Tozzi 2011 [28]	Williams 2003 [22]
Indication	Neck or low back pain	Neck or back pain
Acute vs chronic	Acute/Chronic	(Sub)acute
Intervention/technique	Fascial release	Osteopathic spinal manipulation
Comparison	Sham treatment	Usual care
Number of randomised patients (age range)	120 (IG: 18 female; 21-58; CG: 18 female; 18-56)	201 (female: NR; 16-65[173])

[173] target population

Author, year [reference]		Tozzi 2011 [28]	Williams 2003 [22]
Outcomes (measurements)		Pain (SF-MPQ)[174]	Spinal pain and disability (EASPS), pain (SMPQ), physical and mental health (SF-12), QoL (EQ-5D)
Effectiveness outcomes	Absolute effects (mean (SD); 95% CI; p-value of overall effect)	*Between-group difference* (mean (SD); p-value):<br>Pain:<br>IG: pre: 24.7 (8.6); post: 15.5 (9.8); CG: pre: 24.9 (9.2); post: 25.1 (8.9); p<0.0001	*Improvement in mean scores (mean change) at 2 months:* (mean (SD); 95% CI; p-value):<br>Spinal pain and disability:<br>IG: 13.9 (12.8); CG: 8.6 (14.2); 0.7 to 9.8; p=0.02<br>Pain: IG: 4.6 (8.0); CG: 2.1 (7.0); -0.1 to 5.0; n.s.<br>Physical health: IG: 5.4 (8.9); CG: 4.1 (8.6); -1.7 to 4.3; n.s.<br>Mental health: IG: 7.9 (11.2); CG: 1.2 (12.0); 2.7 to 10.7; p=0.001<br>QoL: IG: 0.11 (0.28); CG: 0.06 (0.29); -0.04 to 0.15; n.s.<br>*Improvement in mean scores (mean change) at 6 months:* (mean (SD); 95% CI; p-value):<br>Spinal pain and disability: IG: 14.9 (16.1); CG: 10.4 (18.0); -1.5 to 10.4; n.s.<br>Pain: IG: 6.6 (8.8); CG: 3.7 (8.1); -0.05 to 5.8; n.s. (p=0.05)<br>Physical health: IG: 7.4 (10.3); CG: 5.5 (9.4); -1.6 to 5.4; n.s.<br>Mental health: IG: 6.8 (13.6); CG: 1.4 (11.3); 1.0 to 9.9; p=0.02<br>QoL: IG: 0.10 (0.30); CG: 0.10 (0.28); -0.1 to 0.1; n.s.
	Relative effects (95% CI; p-value of overall effect)	NR	NR
Safety outcomes	Adverse events: Relative effects (95% CI)	NR	None
	Side effects	NR	NR
Conclusion		Manual fascial techniques are effective manual techniques to improve pain perception over a short-term duration in people with non-specific neck or low back pain.	In a primary care osteopathy clinic, spinal pain and disability, and mental health could be improved.

Abbreviations: CG, control group. EASPS, Extended Aberdeen Spine Pain Scale. EQ-5D, EuroQol. IG, intervention group. n.s., not significant. QoL, quality of life. SF-12, 12-item Short Form Health Survey. SF-MPQ, Short-Form McGill Pain Assessment Questionnaire. SMPQ, Short-form McGill Pain Questionnaire.

Table S16: Summary of effectiveness of included studies: shoulder

Author, year [reference]	Mishra 2018 [26]	Hunter 2022 [29]
Indication	Upper trapezius spasm	Shoulder impingement syndrome
Acute vs chronic	NR	NR
Intervention/technique	Myofascial release	Muscle energy technique
Comparison	Active release technique	Muscle energy technique + soft tissue massage; placebo

[174] All ultrasound measures are not considered as they were not the focus of this report.

Author, year [reference]		Mishra 2018 [26]	Hunter 2022 [29]
Number of randomised patients (age range or mean ± SD)		60 (31 female; 20-55)	75 (IG: 10 female; 62.0 ± 9.6; placebo group: 9 female; 61.4 ± 11.3; muscle energy technique + soft tissue massage group: 9 female; 56.9 ± 9.2)
Outcomes (measurements)		Cervical ROM (neck ROM), neck disability (NDI), pain (VAS)	Arm, shoulder and hand disability (DASH), shoulder pain and disability (SPADI), pain (VAS), change in activities (GROC), activity/functionality (PSFS), ROM (inclinometer; only after treatment)
Effectiveness outcomes	Absolute effects (mean (SD) or mean difference; 95% CI; p-value of overall effect)	*Difference between groups* (mean (SD); p-value *in favour of CG*):<br>Pain: IG: -2.48 (0.86), CG: -4.79 (1.13); p<0.001<br>ROM:<br>*Cervical flexion:* IG: 5.63 (2.22), CG: 11.86 (4.05); p<0.001<br>*Cervical extension:* IG: 6.10 (4.02), CG: 11.70 (4.05); p<0.001<br>*Cervical side flexion (right):* IG: 5.73 (2.65), CG: 8.70 (3.78); p<0.001<br>*Cervical side flexion (left):* IG: 6.13 (4.00), CG: 11.93 (4.77); p<0.001<br>*Cervical rotation (right):* IG: 5.50 (2.82), CG: 9.20 (6.68); p<0.01<br>*Cervical rotation (left):* IG: 5.46 (4.32), CG: 9.86 (5.71); p<0.001<br>Neck disability: IG: -8.66 (4.67), CG: -13.33 (3.69); p<0.001<br>*Intra-group analysis (mean (SD)):*<br>Statistically significant pre-post differences in both groups (p<0.001 in all measurements)<br>Pain intensity:<br>IG: pre: 6.10 (1.17); post: 3.61 (1.26)<br>Mean of the difference of the IG: -2.48 (0.8585)	*Mean difference between groups* (mean difference; 95% CI; p-value):<br>Pain:<br>*IG vs placebo group (CG):*<br>*Week 3:* -15.5 (-24.5 to -6.5); p=0.001; *Week 7:* -10.8 (-20.4 to -1.3); p=0.03; *Week 29:* -14.1 (-26.0 to -2.2); p=0.02; *Week 55:* -17.3 (-30.9 to -3.8); p=0.01<br>*IG vs MET+STM:*<br>*Week 3:* -7.7 (-16.8 to 1.5); n.s.; *Week 7:* -6.6 (-16.2 to 3.0); n.s.<br>*Week 29:* -7.0 (-19.4 to 5.3); n.s.; *Week 55:* -14.8 (-28.6 to -1.1); p=0.04<br>Shoulder pain and disability:<br>*IG vs placebo group (CG):*<br>*Week 3:* -14.7 (-23.0 to -6.3); p=0.001; *Week 7:* -11.8 (-21.8 to -1.9); p=0.020<br>*Week 29:* -14.9 (-26.3 to -3.5); p=0.010; *Week 55:* -19.0 (-32.4, -5.7); p=0.005<br>*IG vs MET+STM:*<br>*Week 3:* -1.1 (-9.8 to 7.5); n.s.; *Week 7:* -5.8 (-16.0 to 4.3); n.s.<br>*Week 29:* -2.5 (-14.4 to 9.4); n.s.; *Week 55:* -7.6 (-21.2, 6.1); n.s.

Author, year [reference]		Mishra 2018 [26]	Hunter 2022 [29]
Effectiveness outcomes *(continuation)*	Absolute effects (mean (SD) or mean difference; 95% CI; p-value of overall effect) *(continuation)*		Arm, shoulder and hand disability:<br>*IG vs placebo group (CG):*<br>*Week 3:* -8.4 (-14.0 to -2.8); p=0.003; *Week 7:* -6.2 (-14.0 to 1.6); n.s.<br>*Week 29:* -11.1 (-18.6 to -3.7); p=0.004; *Week 55:* -13.4 (-23.9 to -2.9); p=0.013<br>*IG vs MET+STM:*<br>*Week 3:* -0.26 (-6.0 to 5.5); n.s.; *Week 7:* -3.4 (-11.2 to 4.5); n.s.<br>*Week 29:* -2.1 (-9.9 to 5.7); n.s.; *Week 55:* -4.1 (-14.8 to 6.7); n.s.<br>Change in activities:<br>*IG vs placebo group (CG):*<br>*Week 3:* 1.5 (0.9 to 2.2); p<0.001; *Week 7:* 1.0 (0.1 to 1.9); p=0.03<br>*Week 29:* 1.0 (-0.1 to 2.1); n.s.; *Week 55:* 1.4 (-0.1 to 2.8); n.s.<br>*IG vs MET+STM:*<br>*Week 3:* 0.3 (-0.4 to 1.0); n.s.; *Week 7:* 0.3 (-0.6 to 1.2); n.s.<br>*Week 29:* -0.2 (-1.3 to 0.9); n.s.; *Week 55:* 0.5 (-1.0 to 2.0); n.s.<br>Activity/functionality:<br>*IG vs placebo group (CG):*<br>*Week 3:* 1.3 (0.1 to 2.5); p=0.03; *Week 7:* 0.8 (-0.4 to 2.1); n.s.<br>*Week 29:* 0.6 (-0.7 to 1.9); n.s.; *Week 55:* 1.8 (0.5 to 3.2); p=0.008<br>*IG vs MET+STM:*<br>*Week 3:* 0.3 (-0.9 to 1.5); n.s.; *Week 7:* 0.7 (-0.5 to 2.0); n.s.<br>*Week 29:* -0.4 (-1.8 to 1.0); n.s.; *Week 55:* 0.2 (-1.2 to 1.5); n.s.<br>ROM:<br>*IG vs placebo group (CG) – week 3:*<br>Standing posture: 1.6 (-1.8 to 5.0); n.s.; Thoracic flexion: -0.2 (-2.6 to 2.1); n.s.<br>Thoracic extension: -1.3 (-5.2 to 2.6); n.s.; Total thoracic ROM: 1.2 (-2.8 to 5.2); n.s.<br>*IG vs MET+STM – week 3:*<br>Standing posture: 0.2 (-3.2 to 3.5); n.s.; Thoracic flexion: 0.5 (-1.9 to 2.8); n.s.<br>Thoracic extension: -1.5 (-5.4 to 2.5); n.s.; Total thoracic ROM: 1.9 (-2.0 to 5.9); n.s.
	Relative effects (95% CI; p-value of overall effect)	NR	NR
Safety outcomes	Adverse events: Relative effects (95% CI)	NR	None
	Side effects	NR	NR
Conclusion		Both techniques are effective in alleviating pain, ROM, and neck disability. However, active release therapy gave better results as compared to myofascial release.	Muscle energy technique of the thoracic spine with or without soft tissue massage improved the pain and disability in individuals with shoulder impingement syndrome and may be recommended as a treatment approach.

Abbreviations: CG, control group. CI, confidence interval. DASH, Disabilities of the Arm Shoulder and Hand questionnaire. GROC, Global Rating of Change. IG, intervention group. MET+STM, muscle energy technique + soft tissue massage. n.s., not significant. NDI, Neck Disability Index scale. NR, not reported. PSFS, Patient-Specific Functional Scale. ROM, range of motion. SD, standard deviation. SPADI, Shoulder Pain and Disability Index. VAS, Visual Analogue Scale.

Table S17: Summary of effectiveness of included studies: lower back

Author, year [reference]		Dal Farra, 2021 [14]
Indication		Low back pain
Acute vs chronic		Chronic
Intervention/technique		Osteopathic interventions
Comparison		No active treatment (sham therapy or no intervention), active treatment (standard exercise, classic massage)
Number of included patients (age mean (SD))		1,160 (female: NR; mean age 43.3 +/- 7.7)
Outcomes (measurements)		Pain (VAS, NRS, MGPQ)<br>Functional status (ODI, RMDQ, QBPDS)
Effectiveness outcomes	Absolute effects (95% CI; p-value of overall effect)	NR
	Relative effects (95% CI; p-value of overall effect)	Pain:<br>ES =-0.59 [-0.81, -0.36]; P<0.00001; heterogeneity: moderate-to-substantial and significant (I^2=59%; P=0.005); 10 studies (12 articles) (n=1,049 patients)<br>FU (12 weeks):<br>ES = -0.73 [-1.09, -0.37]; P<0.0001; heterogeneity: heterogenous and not significant (I^2=0%; P=0.93);<br>2 studies (n=128 patients)<br><br>Functional status:<br>ES=-0.42 [-0.68, -0.15]; P=0.002; heterogeneity: substantial and significant (I^2=72%; P<0.0001); 10 studies (12 articles) (n=1,055 patients)<br>FU (12 weeks):<br>ES= -0.32 [-0.74, 0.09]; P=0.13; heterogeneity: substantial and significant (I^2=77%; P=0.002);<br>4 studies (5 articles) (n=676 patients)
Safety outcomes	Adverse events: Relative effects (95% CI)	Increased pain in 10 subjects during the first week of myofascial release treatment (1/10 study);<br>Increased back muscle spasticity in one occasion (1/10 study);<br>No data collection (1/10 study);<br>NR<br>(7/10 studies)
	Side effects	NR
Conclusion		Osteopathy is effective in pain levels and functional status improvements in chronic low<br>back pain patients. Myofascial release reported better level of evidence for pain reduction<br>if compared to other interventions.

Abbreviations: ES, effect size. FU, follow-up. MGPQ, McGill Pain Questionnaire. NR, not reported. NRS, Numerical Rating Scale. ODI, Oswestry Disability Index. QBPDS, Quebec Pain Disability Scale. RMDQ, Roland and Morris Disability Questionnaire. VAS, Visual Analogue Scale.

Table S18: Summary of effectiveness of included studies: knee

Author, year [reference]		Zago 2021 [30]	Licciardone 2004 [32]
Indication		Patellofemoral pain syndrome	Knee or hip osteoarthritis, or hip fracture
Acute vs chronic		Chronic	Acute
Intervention/technique		OMT	One or a combination of: myofascial release, strain—counterstrain, muscle energy, soft tissue, high-velocity low-amplitude (not at the surgical site), or craniosacral manipulation
Comparison		Exercise programme & waiting list	Sham treatment (range-of-motion activities, light touch)
Number of randomised patients (age range)		82 (48 female; 18–35)	60 (42 female; 69.2 (10.3))
Outcomes (measurements)		Pain (VAS), functionality (LKSS), dynamic knee valgus (SDT), plantar pressure in middle foot (SB), posterior thigh flexibility (SRT),<br>hip ROM (fleximetry)	Functional independence (FIM), daily analgesic medication use (mg/d), length of stay (days), rehabilitation efficiency (FIM total score change per rehabilitation<br>unit day), general health (SF-36)
Effectiveness outcomes	Absolute effects (mean (SD); 95% CI; p-value of overall effect)	*Change from admission to discharge* (mean (SD); p-value):<br>Functional independence: OMT: 26.5 (7.0); CG: 26.2 (6.5); n.s.<br>Daily analgesic medication use:<br>*Acetaminophen:* OMT: -741 (1471); CG: -371 (1715); n.s.<br>*Hydrocodone:* OMT: -9.9 (16.9); CG: -8.0 (13.3); n.s.<br>*At rehabilitation unit discharge* (mean (SD); p-value):<br>Length of stay: OMT: 15.4 (6.6); CG: 12.3 (7.4); n.s.<br>Rehabilitation efficiency: OMT: 2.0 (0.7); CG: 2.6 (1.1); p=0.01<br>*Change from admission to 4 weeks after discharge* (mean (SD); p-value):<br>General health:<br>*Physical functioning:* OMT: -10.0 (31.3); CG: -15.0 (27.2); n.s.<br>*Physical role limitations:* OMT: -16.3 (42.4); CG: -7.0 (37.9); n.s.<br>*Bodily pain:* OMT: 22.9 (36.7); CG: 13.3 (38.0); n.s.<br>*General health:* OMT: 4.9 (19.9); CG: 3.3 (17.9); n.s.<br>*Vitality:* OMT: 9.2 (23.2); CG: 9.0 (33.7); n.s.<br>*Social functioning:* OMT: 16.4 (41.5); CG: 1.0 (32.5); n.s.<br>*Emotional role limitations:* OMT: 24.4 (58.5); CG: 22.7 (45.9); n.s.<br>*Mental health:* OMT: 10.6 (23.4); CG: 4.8 (12.7); n.s.	*Difference between groups after 3 weeks* (mean (SD); (95% confidence interval)); p-value):<br>Pain:<br>OMT group: −6.56 (2.03); (−7.64 to −5.48); EP group: −4.43 (1.26); (−5.11 to −3.76);<br>CG: −0.18 (0.91); (−0.67 to 0.29); OMT vs EP: NR; OMT vs CG: p<0.05<br>Functionality:<br>OMT group: 31.86 (18.41); (21.23 to 42.49); EP group: 19.75 (13.45); (12.58 to 26.92);<br>CG: −0.37 (2.27); (−1.58 to 0.83); OMT vs EP: s.s.; OMT vs CG: NR<br>Dynamic knee valgus:<br>OMT group: 7.81 (5.30); (4.98 to 10.64); EP group: 1.50 (9.47); (−3.54 to 6.54);<br>CG: 1.81 (10.54); (−3.80 to 7.42); OMT vs EP: s.s.; OMT vs CG: s.s.<br>Plantar pressure in middle foot:<br>OMT group: −0.42 (0.16); (−0.51 to −0.33); EP group: −0.18 (0.28); (−0.33 to −0.02);<br>CG: −0.02 (0.30); (−0.18 to 0.13); OMT vs EP: s.s.; OMT vs CG: s.s.<br>Posterior thigh flexibility:<br>OMT group: 5.62 (4.20); (3.37 to 7.86); EP group: 6.13 (4.01); (3.99 to 8.27);<br>CG: −1.08 (1.54); (−1.90 to −0.25); OMT vs EP: NR; OMT vs CG: s.s.<br>Hip ROM:<br>OMT group: −0.93 (12.71); (−7.71 to 5.83); EP group: 6.50 (9.73); (1.31 to 11.68);<br>CG: −0.68 (3.32); (−2.45 to 1.08); OMT vs EP: n.s.; OMT vs CG: n.s.<br>*Difference between groups at 30-day FU* (mean (SD) (95% confidence interval)):

Author, year [reference]		Zago 2021 [30]	Licciardone 2004 [32]
			Pain:<br>OMT group: −6.56 (2.03); (−7.65 to −5.48); EP group: −4.43 (1.26); (−5.11 to −3.76);<br>CG: 0.50 (1.03); (−1.05 to 0.05); s.s.<br>Functionality:<br>OMT group: 30.21 (19.72); (18.83 to 41.60); EP group: 18 (13.02); (11.06 to 24.94);<br>CG: 1.06 (3.31); (−0.70 to 2.82); s.s.
	Absolute effects (mean (SD); 95% CI; p-value of overall effect) *(continuation)*		Dynamic knee valgus:<br>OMT group: 10 (6.14); (6.72, 13.27); EP group: 1.50 (9.47); (−3.54 to 6.54);<br>CG: −0.75 (10.18); (−6.17 to 4.67); n.s.<br>Plantar pressure in middle foot:<br>OMT group: −0.40 (0.21); (−0.52 to −0.29); EP group: −0.18 (0.28); (−0.33 to −0.02);<br>CG: −0.02 (0.10); (−0.08 to 0.03); s.s.<br>Posterior thigh flexibility:<br>OMT group: 5.58 (3.74); (3.58 to 7.57); EP group: 5.58 (4.04); (3.43 to 7.74);<br>CG: −0.77 (1.54); (−1.59 to 0.04); n.s.<br>Hip ROM:<br>OMT group: 5.31 (5.32); (−5.56 to 16.19); EP group: 6.56 (5.43); (−4.54 to 17.67);<br>CG: −0.59 (5.25); (−11.32 to 10.13); n.s.<br>*Intra-group pre-post difference of the IG* (mean (SD)):<br>Pain: pre: 6.62 (2.02); post: 0.62 (0.25); FU: 0.06 (0.25)
	Relative effects (95% CI; p-value of overall effect)	NR	NR
Safety outcomes	Adverse events: Relative effects (95% CI)	NR	NR
	Side effects	NR	NR
Conclusion		Both OMT and EP are effective in reducing knee pain in runners with PFPS, but there were no differences between the 2 interventions. However, OMT had greater effects on PPMF, DKV, and ROM of hip extension.	OMT does not appear to be efficacious in acute rehabilitation patients who recently underwent surgery for knee or hip osteoarthritis or a hip fracture. The only significant difference between groups was decreased rehabilitation efficiency with OMT.

Abbreviations: CG, control group. EP, exercise programme. FIM, Functional Independence Measure. FU, follow-up. LKSS, Lysholm Knee Scoring Scale. n.s., not significant. NR, not reported. OMT, osteopathic manipulative treatment. ROM, range of motion. SB, static baropodometry. SDT, step-down test. SF-36, Medical Outcomes Study Short Form-36. SRT, sit and reach test. s.s., statistically significant. VAS, Visual Analogue Scale.

Table S19: Summary of effectiveness of included studies: foot

Author, year [reference]		Bac 2022 [33]	Ajimsha 2014 [31]
Indication		Flat foot with foot pain	Unilateral plantar heel pain
Acute vs chronic		NR	NR
Intervention/technique		Myofascial release	Myofascial release
Comparison		Exercise programme; myofascial release and exercise programme[169];<br>no intervention	Sham ultrasound therapy
Number of randomised patients (age range or mean ± SD)		70 (47 female after dropout; 20–49)	66 (49 female; IG: 42.4 ± 4.6; CG: 40.8 ± 7.1)
Outcomes (measurements)		Pain intensity (NRS scale), foot load distribution and selected static/dynamic foot indicators (FreeMed ground reaction force platform)	Pain, disability and activity restriction (FFI), pressure pain thresholds (mechanical pressure algometer)
Effectiveness outcomes	Absolute effects (mean (SD) or mean ± SD (95% CI of the mean);<br>p-value of overall effect)	*Differences before and after*[175] (mean (SD);<br>p-value of between measurements comparison):<br>Pain intensity:<br>*Left foot:*<br>IG: −3.26 (2.54); p=0.002; E: −1.93 (2.12); p=0.012; CG: −0.80 (1.69); n.s.<br>Between-group comparison (p-value): IG vs E: n.s.; IG vs CG: p=0.018<br>*Right foot:*<br>IG: −2.66 (1.63); p=0.001; E: −1.66 (1.79); p=0.012; CG: −0.80 (1.61); n.s.<br>Between-group comparison (p-value): IG vs E: n.s.; IG vs CG: p=0.015	*Differences after 4 weeks* (mean ± SD (95% CI of the mean); p-value):<br>Pain, disability and activity restriction: IG: pre: 63.01 ± 4.44 (59.43–64.79), post: 17.39 ± 4.02 (16.08–21.26); 72.4% reduction; CG: pre: 61.38 ± 5.22 (58.58–64.15), post: 56.85 ± 6.91 (53.02–58.88); 7.4% reduction; group comparison: p<0.001<br>Pressure pain thresholds (group-by-time interactions for changes)**:**<br>*Gastrocnemius:* IG: pre: 1.8 ± 0.44 (1.7–2.1), post: 2.9 ± 0.82 (2.8–3.1); CG: pre: 2.0 ± 0.22 (1.8–2.1), post: 2.2 ± 0.51 (2.0–2.4); p<0.001<br>*Soleus:* IG: pre: 2.0 ± 0.48 (1.9–2.2), post: 3.1 ± 0.91 (2.8–3.2); CG: pre: 2.2 ± 0.52 (2.0–2.4), post: 2.2 ± 0.31 (2.1–2.3); p<0.001<br>*Calcaneus:* IG: pre: 2.1 ± 0.38 (1.9–2.2), post: 3.4 ± 0.95 (3.1–3.6); CG: pre: 2.3 ± 0.77 (2.2–2.7), post: 2.5 ± 0.67 (2.3–2.6); p<0.001<br>*Differences after 12 weeks* (mean ± SD (95% CI of the mean):<br>Pain, disability and activity restriction: IG: post: 24.81 ± 3.98 (22.73–26.89); 60.6% reduction; CG: post: 60.15 ± 8.11 (56.05–63.26); 2.0% reduction; group comparison: p<0.001<br>Pressure pain thresholds:<br>*Gastrocnemius:* IG: post: 2.6 ± 0.54 (2.4–2.7); CG: post: 2.1 ± 0.32 (2.0–2.2); p<0.001<br>*Soleus:* IG: post: 2.7 ± 0.65 (2.6–2.9); CG: post: 2.1 ± 0.72 (2.0–2.3); p<0.001<br>*Calcaneus:* IG: post: 3.1 ± 0.78 (2.9–3.2); CG: post: 2.4 ± 0.48 (2.2–2.7); p<0.001
	Relative effects (95% CI; p-value of overall effect)	NR	NR
Safety outcomes	Adverse events: Relative effects (95% CI)	NR	Serious adverse events: none

[175] Foot load distribution was not extracted as it was not the objective of this report.

Author, year [reference]		Bac 2022 [33]	Ajimsha 2014 [31]
	Side effects	NR	NR
Conclusion		A limited influence of both exercises and myofascial release techniques on pain and selected static and dynamic indicators of a flat foot could be observed.	Myofascial release was more effective than sham ultrasound therapy regarding pain, disability and activity restriction, and pressure pain.

Abbreviations: CG, control group. CI, confidence interval. E, exercise group. FFI, Foot Function Index. IG, intervention group. NR, not reported. NRS, Numerical Rating Scale. SD, standard deviation.

Table S20: Summary of effectiveness of included studies: osteoporosis

Author, year [reference]		Papa 2012 [23]
Indication		Osteoporosis
Acute vs chronic		NR
Intervention/technique		OMT
Comparison		Sham manipulative treatment
Number of randomised patients (age mean (SD))		72 (51 female; IG: 77.2 (5.3); CG: 76.8 (8.2))
Outcomes (measurements)		Pain (VAS), QoL (QUALEFFO-41)
Effectiveness outcomes	Absolute effects (mean ± SD; p-value of overall effect)	*Between-group difference* (mean ± SD; p-value):<br>Pain:<br>IG: pre: 4.4 ± 2.6, post: 4.1 ± 1.9; CG: pre: 4.8 ± 2.5, post: 4.6 ± 2.7; n.s.<br>QoL:<br>IG: pre: 107 ± 25, post: 91 ± 29; CG: pre: 112 ± 27, post: 110 ± 31; p=0.001<br>*Subscales:*<br>Pain: p=0.003; Perception of health: p=0.005; Path/Mobility: p=0.049; Mental well-being: n.s.; Daily activities: n.s.; Housework: n.s.; Leisure activities: n.s.
	Relative effects (95% CI; p-value of overall effect)	NR
Safety outcomes	Adverse events: Relative effects (95% CI)	None
	Side effects	NR
Conclusion		In a group of elderly subjects affected by osteoporosis, OMT was able to increase self-reported QoL, while the effect on body pain perception is unclear.

Abbreviations: OMT, osteopathic manipulative treatment. QoL, quality of life. QUALEFFO-41, Quality of Life Questionnaire of the European Foundation for Osteoporosis. VAS, Visual Analogue Scale.

Table S21: Summary of effectiveness of included studies: fibromyalgia

Author, year [reference]		Matarán-Penarrocha 2011 [27]	Castro-Sanchez 2011 [15]
Indication		Fibromyalgia	Fibromyalgia
Acute vs chronic		Chronic	Chronic
Intervention/technique		Craniosacral therapy	Myofascial release
Comparison		Placebo (simulated treatment with disconnected ultrasound)	Sham short-wave and ultrasound electrotherapy
Number of randomised patients (age range; mean (SD))		Randomised: 104<br>Analysed: 84 (81 female; range 34–63; mean 49.08 ± 14.17)	94 (female: NR; range 45-65; mean 54.4)
Outcomes (measurements)		Pain (VAS), QoL (SF-36), sleep quality (PSQI), depression (BDI), anxiety (STAI)	Physical functioning (FIQ), mood (NDFG), pain (MPQ), fatigue (FIQ), tiredness on walking (FIQ), stiffness (FIQ), pain: sensory (MPQ), pain: affective (MPQ), pain: sensory + affective (MPQ), pain (VAS), clinical severity (CGIs), clinical improvement (CGIi), postural stability (stabilometer platform)[176]
Effectiveness outcomes	Absolute effects (mean (SD); p-value of overall effect)	*25 weeks after intervention* (mean (SD); p-value):<br>Pain: IG: p<0.05; CG: NR; between-group difference: p<0.05<br>QoL:<br>*Physical function:* IG: pre: 49.43 (6.90), post: 45.90 (5.87); p<0.05;<br>CG: pre: 51.90 (9.92), post: 50.53 (9.12); n.s.; between-group difference: p<0.01<br>*Physical role:* IG: pre: 25.17 (6.88), post: 22.10 (6.84); p<0.05;<br>CG: pre: 25.86 (7.35), post: 25.80 (6.98); n.s.; between-group difference: p<0.05<br>*Body pain:* IG: pre: 75.76 (7.20), post: 73.12 (6.08); p<0.05;<br>CG: pre: 78.43 (12.75), post: 78.00 (13.07); n.s.; between-group difference: p<0.05<br>*General health:* IG: pre: 67.02 (4.25), post: 64.40 (4.65); p<0.05;<br>CG: pre: 68.28 (6.84), post: 68.35 (6.39); n.s.; between-group difference: p<0.05<br>*Vitality:* IG: pre: 60.05 (5.23), post: 62.73 (5.27); p<0.05;<br>CG: pre: 58.90 (6.27), post: 59.48 (7.73); n.s.; between-group difference: p<0.05<br>*Social function*: IG: pre: 63.23 (7.12), post: 58.75 (6.74); p<0.05;<br>CG: pre: 63.93 (12.41), post: 63.50 (11.57); n.s.; between-group difference: p<0.05<br>*Emotional role:* between-group difference: n.s.<br>*Mental health:* between-group difference: n.s.<br>State anxiety: IG: p<0.05; CG: n.s.; between-group difference: n.s.<br>Trait anxiety: IG: p<0.05; CG: n.s.; between-group difference: p<0.05<br>Depression: between-group difference: n.s.<br>Sleep quality: IG: p<0.05**;** CG: NR; between-group difference<br>*(only sleep duration/disturbance, subjective quality)*: p<0.05	*Differences between groups* (mean (SD); p-value):<br>Pain (MPQ):<br>*Pre:* IG: 9.2 (0.6), CG: 8.9 (1.1); *20 weeks:* IG: 7.3 (1.4), CG: 8.2 (1.1); p=0.036<br>*6 months:* IG: 8.5 (0.7), CG: 8.0 (1.3); p=0.042; *1 year:* IG: 8.8 (0.5), CG: 8.7 (0.7); n.s.<br>Pain: sensory:<br>*Pre:* IG: 19.3 (9.2), CG: 19.9 (10.6); *20 weeks:* IG: 16.5 (8.6), CG: 20.3 (6.5); p=0.021<br>*6 months:* IG: 17.3 (7.8), CG: 20.7 (7.1); p=0.042; *1 year:* IG: 18.2 (8.3), CG: 21.2 (7.9); p=0.038<br>Pain: affective:<br>*Pre:* IG: 5.6 (3.4), CG: 4.9 (4.2); *20 weeks:* IG: 4.2 (3.4), CG: 5.3 (4.1); p=0.029<br>*6 months:* IG: 4.5 (2.9), CG: 5.2 (3.8); p=0.042; *1 year:* IG: 4.8 (3.6), CG: 5.1 (2.9); n.s.<br>Pain: sensory + affective:<br>*Pre:* IG: 24.9 (12.6), CG: 25.3 (10.7); *20 weeks:* IG: 20.6 (6.3), CG: 25.9 (5.3); p=0.019<br>*6 months:* IG: 21.9 (7.2), CG: 26.2 (6.8); p=0.022; *1 year:* IG: 23.2 (7.6), CG: 26.7 (6.9); p=0.036<br>Pain (VAS):<br>*Pre:* IG: 9.13 (0.8), CG: 8.90 (1.3); *20 weeks:* IG: 7.98 (1.03), CG: 8.87 (1.01); p=0.038<br>*6 months:* IG: 8.25 (1.13), CG: 8.94 (1.34); p=0.043; *1 year:* IG: 8.74 (1.08), CG: 8.92 (0.96); n.s.<br>Physical functioning:<br>*Pre:* IG: 64.95 (18.2), CG: 63.94 (16.4); *20 weeks:* IG: 56.10 (17.3), CG: 65.85 (18.5); p=0.038<br>*6 months:* IG: 58.60 (16.3), CG: 64.08 (18.1); p=0.048; *1 year:* IG: 62.80 (20.1), CG: 65.01 (19.8); n.s.<br>Mood:<br>*Pre:* IG: 1.84 (1.56), CG: 2.04 (2.10); *20 weeks:* IG: 3.24 (1.46), CG: 1.96 (1.67); p=0.028<br>*6 months:* IG: 2.88 (1.56), CG: 2.01 (1.44); p=0.036; *1 year:* IG: 2.55 (1.76), CG: 1.99 (1.62); p=0.047
	Absolute effects (mean (SD); p-value of overall effect) *(continuation)*	*6 months post-intervention* (mean (SD); p-value):<br>State anxiety, depression, pain: IG: n.s.; CG: n.s.; between-group difference: n.s.<br>QoL:<br>*Physical function:* IG: post: 46.05 (4.61) p<0.05; CG: post: 49.05 (8.03); n.s.; between-group difference: p<0.05	Fatigue:<br>*Pre:* IG: 8.1 (1.5), CG: 8.6 (1.3); *20 weeks:* IG: 7.2 (2.2), CG: 8.7 (1.9); p=0.026<br>*6 months:* IG: 7.4 (1.9), CG: 8.5 (1.7); p=0.037; *1 year:* IG: 7.8 (2.3), CG: 8.8 (1.6); p=0.038

[176] The analyses of tender points were not considered as they were not the focus of this report.

Author, year [reference]		Matarán-Penarrocha 2011 [27]	Castro-Sanchez 2011 [15]
		Vitality: IG: post: 60.80 (5.11); n.s.; CG: post: 58.72 (7.78); n.s.; between-group difference: p<0.05<br>*All other items:* IG: n.s.; CG: n.s.; between-group difference: n.s.<br>Sleep quality: between-group difference *(only sleep duration/disturbance, habitual sleep efficiency)*: p<0.05<br>*1 year post-intervention* (mean (SD); p-value):<br>Sleep quality *(only sleep duration, habitual sleep efficiency, daily dysfunction)*: IG: p<0.05; CG: NR; between-group difference: p<0.05<br>Anxiety, depression, pain, QoL: IG: n.s.; CG: n.s.; between-group difference: n.s.	Tiredness on walking:<br>*Pre:* IG: 8.5 (2.3), CG: 7.9 (2.6); *20 weeks:* IG: 7.1 (2.1), CG: 7.9 (2.3); p=0.044<br>*6 months:* IG: 7.5 (1.9), CG: 7.6 (1.8); n.s.; *1 year:* IG: 7.8 (2.2), CG: 7.7 (1.9); n.s.<br>Stiffness:<br>*Pre:* IG: 7.8 (1.9), CG: 6.9 (2.7)[177]; *20 weeks:* IG: 6.6 (2.8), CG: 7.5 (1.9); p=0.042<br>*6 months:* IG: 6.9 (2.5), CG: 7.8 (2.4); p=0.043; *1 year:* IG: 7.3 (2.5), CG: 7.8 (2.1); n.s.<br>Clinical severity:<br>*Pre:* IG: 6.25 (0.73), CG: 5.92 (0.84); *20 weeks:* IG: 5.08 (1.03), CG: 6.02 (0.96); p=0.044<br>*6 months:* IG: 5.28 (0.97), CG: 5.98 (0.84); p=0.048; *1 year:* IG: 5.49 (0.74), CG: 6.17 (0.91); n.s.<br>Clinical improvement:<br>*Pre: IG:* −5.38 (0.79), CG: −5.47 (0.46); *20 weeks:* IG: 5.28 (0.97), CG: 6.13 (1.03); p=0.043<br>*6 months:* IG: 5.62 (0.88), CG: 6.30 (0.97); 0.046; *1 year:* IG: 5.83 (1.24), CG: 6.49 (0.89); p=0.049<br>Postural stability:<br>*Pre:* IG: NR, CG: NR; *20 weeks:* IG: 5.10 (1.89), CG: 5.49 (0.94); n.s.<br>*6 months:* IG: 5.42 (1.97), CG: 5.52 (1.06); n.s.; *1 year:* IG: 5.39 (1.24), CG: 5.50 (1.37); n.s.
	Relative effects (95% CI; p-value of overall effect)	NR	NR
Safety outcomes	Adverse events: Relative effects (95% CI)	None	None
	Side effects	NR	NR
Conclusion		Approaching fibromyalgia through craniosacral therapy improves anxiety and QoL levels. Craniosacral therapy reduces the perception of pain and fatigue and improves their night rest, increasing physical function.	Myofascial release techniques can be a complementary therapy for pain symptoms, physical function and clinical severity but do not improve postural stability in patients with fibromyalgia syndrome.

Abbreviations: BDI, Beck depression inventory. CG, control group. CGIi, Clinical Global Impression Improvement Scale. CGIs, Clinical Global Impression Severity Scale. FIQ, 10-item Fibromyalgia Impact Questionnaire. IG, intervention group. MPQ, McGill Pain Questionnaire. n.s., not significant. NDFG, number of days feeling good. NR, not reported. PSQI, Pittsburgh Sleep Quality Index. QoL, quality of life. SF-36, short form-36 health survey. STAI, State Trait Anxiety Inventory. VAS, Visual Analogue Scale.

[177] The two groups statistically significantly differed in the pre testing.

Literature search strategies for the primary search

Search strategy for Embase

Search date: 18.05.2022		
No.	Query Results	Results
#62.	#60 NOT #61	621
#61.	#60 AND 'Conference Abstract'/it	120
#60.	#59 AND ([english]/lim OR [german]/lim)	741
#59.	#17 OR #53 OR #58	756
#58.	#57 AND [2017-2022]/py	166
#57.	#54 OR #56	376
#56.	#16 AND #55	366
#55.	('meta analysis'/exp OR 'systematic review'/exp OR ((meta NEAR/3 analy*):ab,ti) OR metaanaly*:ab,ti OR review*:ti OR overview*:ti OR ((synthes* NEAR/3 (literature* OR research* OR studies OR data)):ab,ti) OR (pooled AND analys*:ab,ti) OR (((data NEAR/2 pool*):ab,ti) AND studies:ab,ti) OR medline:ab,ti OR medlars:ab,ti OR embase:ab,ti OR cinahl:ab,ti OR scisearch:ab,ti OR psychinfo:ab,ti OR psycinfo:ab,ti OR psychlit:ab,ti OR psyclit:ab,ti OR cinhal:ab,ti OR cancerlit:ab,ti OR cochrane:ab,ti OR bids:ab,ti OR pubmed:ab,ti OR ovid:ab,ti OR (((hand OR manual OR database* OR computer*) NEAR/2 search*):ab,ti) OR ((electronic NEAR/2 (database* OR 'data base' OR 'data bases')):ab,ti) OR bibliograph*:ab OR 'relevant journals':ab OR (((review* OR overview*) NEAR/10 (systematic* OR methodologic* OR quantitativ* OR research* OR literature* OR studies OR trial* OR effective*)):ab)) NOT ((((retrospective* OR record* OR case* OR patient*) NEAR/2 review*):ab,ti) OR (((patient* OR review*) NEAR/2 chart*):ab,ti) OR rat:ab,ti OR rats:ab,ti OR mouse:ab,ti OR mice:ab,ti OR hamster:ab,ti OR hamsters:ab,ti OR animal:ab,ti OR animals:ab,ti OR dog:ab,ti OR dogs:ab,ti OR cat:ab,ti OR cats:ab,ti OR bovine:ab,ti OR sheep:ab,ti) NOT ('editorial'/exp OR 'erratum'/de OR 'letter'/exp) NOT (('animal'/exp OR 'nonhuman'/exp) NOT (('animal'/exp OR 'nonhuman'/exp) AND 'human'/exp))	1,459,069
#54.	#16 AND ([cochrane review]/lim OR [systematic review]/lim OR [meta analysis]/lim)	181
#53.	#16 AND #52	601
#52.	#37 NOT #51	5,146,497
#51.	#38 OR #39 OR #40 OR #41 OR #42 OR #43 OR #44 OR #45 OR #46 OR #47 OR #48 OR #49 OR #50	3,981,020
#50.	'animal experiment'/de NOT ('human experiment'/de OR 'human'/de)	2,425,194
#49.	(rat:ti,tt OR rats:ti,tt OR mouse:ti,tt OR mice:ti,tt OR swine:ti,tt OR porcine:ti,tt OR murine:ti,tt OR sheep:ti,tt OR lambs:ti,tt OR pigs:ti,tt OR piglets:ti,tt OR rabbit:ti,tt OR rabbits:ti,tt OR cat:ti,tt OR cats:ti,tt OR dog:ti,tt OR dogs:ti,tt OR cattle:ti,tt OR bovine:ti,tt OR monkey:ti,tt OR monkeys:ti,tt OR trout:ti,tt OR marmoset*:ti,tt) AND 'animal experiment'/de	1,155,971
#48.	(databases NEAR/5 searched):ab	54,263
#47.	'update review':ab	123
#46.	'we searched':ab AND (review:ti,tt OR review:it)	41,678
#45.	review:ab AND review:it NOT trial:ti,tt	980,019
#44.	('random cluster' NEAR/4 sampl*):ti,ab,tt	1,555
#43.	'random field*':ti,ab,tt	2,661
#42.	nonrandom*:ti,ab,tt NOT random*:ti,ab,tt	17,786
#41.	'systematic review':ti,tt NOT (trial:ti,tt OR study:ti,tt)	208,879
#40.	'case control*':ti,ab,tt AND random*:ti,ab,tt NOT ('randomised controlled':ti,ab,tt OR 'randomized controlled':ti,ab,tt)	19,707
#39.	'cross-sectional study' NOT ('randomized controlled trial'/de OR 'controlled clinical study'/de OR 'controlled study'/de OR 'randomised controlled':ti,ab,tt OR 'randomized controlled':ti,ab,tt OR 'control group':ti,ab,tt OR 'control groups':ti,ab,tt)	331,157
#38.	((random* NEXT/1 sampl* NEAR/8 ('cross section*' OR questionnaire* OR survey OR surveys OR database OR databases)):ti,ab,tt) NOT ('comparative study'/de OR 'controlled study'/de OR 'randomised controlled':ti,ab,tt OR 'randomized controlled':ti,ab,tt OR 'randomly assigned':ti,ab,tt)	2,855
#37.	#18 OR #19 OR #20 OR #21 OR #22 OR #23 OR #24 OR #25 OR #26 OR #27 OR #28 OR #29 OR #30 OR #31 OR #32 OR #33 OR #34 OR #35 OR #36	5,808,135
#36.	trial:ti,tt	364,652
#35.	'human experiment'/de	576,775
#34.	volunteer:ti,ab,tt OR volunteers:ti,ab,tt	269,222
#33.	(controlled NEAR/8 (study OR design OR trial)):ti,ab,tt	415,712
#32.	assigned:ti,ab,tt OR allocated:ti,ab,tt	446,741

#31.	((assign* OR match OR matched OR allocation) NEAR/6 (alternate OR group OR groups OR intervention OR interventions OR patient OR patients OR subject OR subjects OR participant OR participants)):ti,ab,tt	418,365
#30.	crossover:ti,ab,tt OR 'cross over':ti,ab,tt	116,567
#29.	(parallel NEXT/1 group*):ti,ab,tt	29,355
#28.	'double blind procedure'/de	195,409
#27.	((double OR single OR doubly OR singly) NEXT/1 (blind OR blinded OR blindly)):ti,ab,tt	258,280
#26.	(open NEXT/1 label):ti,ab,tt	96,567
#25.	(evaluated:ab OR evaluate:ab OR evaluating:ab OR assessed:ab OR assess:ab) AND (compare:ab OR compared:ab OR comparing:ab OR comparison:ab)	2,493,827
#24.	compare:ti,tt OR compared:ti,tt OR comparison:ti,tt	587,566
#23.	placebo:ti,ab,tt	341,563
#22.	'intermethod comparison'/de	285,035
#21.	'randomization'/de	93,809
#20.	random*:ti,ab,tt	1,787,537
#19.	'controlled clinical trial'/de	436,903
#18.	'randomized controlled trial'/de	710,556
#17.	#16 AND [randomized controlled trial]/lim	297
#16.	#7 AND #15	2,447
#15.	#8 OR #9 OR #10 OR #11 OR #12 OR #13 OR #14	11,245
#14.	'myofascial release'	606
#13.	'myofascial release'/exp	138
#12.	(craniosacral OR 'cranio sacral') NEAR/1 (therap* OR treatment* OR manipulat*)	262
#11.	'craniosacral therapy'/exp	196
#10.	osteopat*:ti,ab,lnk,kw,de	10,536
#9.	'osteopathic manipulation'/exp	546
#8.	'osteopathic medicine'/exp	5,475
#7.	#1 OR #2 OR #3 OR #4 OR #5 OR #6	2,020,431
#6.	backache*	64,460
#5.	neckache*	36
#4.	headache*	328,548
#3.	pain* OR ache* OR aching OR sore*	1,769,208
#2.	'headache and facial pain'/exp	353,024
#1.	'musculoskeletal pain'/exp	174,096

Search strategy for Cochrane

Search Name: Osteopathy for musculoskeletal pain

Last Saved: 18/05/2022 13:46:37

Comment: LG/VH 180522

ID Search

#1 MeSH descriptor: [Pain] explode all trees

#2 MeSH descriptor: [Musculoskeletal Pain] explode all trees

#3 MeSH descriptor: [Shoulder Pain] explode all trees

#4 MeSH descriptor: [Headache Disorders] explode all trees

#5 ((pain* OR ache* OR aching OR sore*)) (Word variations have been searched)

#6 (headache*) (Word variations have been searched)

#7 (neckache*) (Word variations have been searched)

#8 (backache*) (Word variations have been searched)

#9 #1 OR #2 OR #3 OR #4 OR #5 OR #6 OR #7 OR #8

#10 MeSH descriptor: [Osteopathic Medicine] explode all trees

#11 MeSH descriptor: [Manipulation, Osteopathic] explode all trees

#12 (osteopat*) (Word variations have been searched)

#13 ((craniosacral OR cranio-sacral) NEAR (therap* OR treatment* OR manipulat*)) (Word variations have been searched)

#14 MeSH descriptor: [Myofascial Release Therapy] explode all trees

#15 ("myofascial release") (Word variations have been searched)

#16 #10 OR #11 OR #12 OR #13 OR #14 OR #15

#17 #9 AND #16

#18 #9 AND #16 in Trials

#19 #9 AND #16 with Cochrane Library publication date Between Jan 2017 and May 2022, in Cochrane Reviews, Cochrane Protocols

#20 #18 OR #19

#21 (conference abstract):pt

#22 (abstract):so

#23 (clinicaltrials OR trialsearch OR ANZCTR OR ensaiosclinicos OR Actrn OR chictr OR cris OR ctri OR registroclinico OR clinicaltrialsregister OR DRKS OR IRCT OR Isrctn OR rctportal OR JapicCTI OR JMACCT OR jRCT OR JPRN OR Nct OR UMIN OR trialregister OR PACTR OR R.B.R.OR REPEC OR SLCTR OR Tcr):so (Word variations have been searched)

#24 #21 OR #22 OR #23

#25 #20 NOT #24

458 Hits

Search strategy for INAHTA

Search step #	Search query,"Hits","Searched At"
18	(((backache*) OR (neckache*) OR (headache*) OR (pain* OR ache* OR aching OR sore*) OR ("Headache Disorders"[mhe]) OR ("Shoulder Pain"[mhe]) OR ("Musculoskeletal Pain"[mhe]) OR

	("Pain"[mhe])) AND ((myofascial release) OR ("Myofascial Release Therapy"[mhe]) OR ((craniosacral OR cranio-sacral) AND (therap* OR treatment* OR manipulat*)) OR (osteopat*) OR ("Manipulation Osteopathic"[mhe]) OR ("Osteopathic Medicine"[mhe]))) AND (English OR German)[Language],"9","2022-05-18T13:11:06.000000Z"
17	((backache*) OR (neckache*) OR (headache*) OR (pain* OR ache* OR aching OR sore*) OR ("Headache Disorders"[mhe]) OR ("Shoulder Pain"[mhe]) OR ("Musculoskeletal Pain"[mhe]) OR ("Pain"[mhe])) AND ((myofascial release) OR ("Myofascial Release Therapy"[mhe]) OR ((craniosacral OR cranio-sacral) AND (therap* OR treatment* OR manipulat*)) OR (osteopat*) OR ("Manipulation Osteopathic"[mhe]) OR ("Osteopathic Medicine"[mhe])),"9","2022-05-18T13:09:58.000000Z"
16	(backache*) OR (neckache*) OR (headache*) OR (pain* OR ache* OR aching OR sore*) OR ("Headache Disorders"[mhe]) OR ("Shoulder Pain"[mhe]) OR ("Musculoskeletal Pain"[mhe]) OR ("Pain"[mhe]),"1427","2022-05-18T13:09:37.000000Z"
15	backache*,"1","2022-05-18T13:08:55.000000Z"
14	neckache*,"0","2022-05-18T13:08:44.000000Z"
13	headache*,"113","2022-05-18T13:08:27.000000Z"
12	pain* OR ache* OR aching OR sore*,"1232","2022-05-18T13:08:11.000000Z"
11	"Headache Disorders"[mhe],"58","2022-05-18T13:07:41.000000Z"
10	"Shoulder Pain"[mhe],"9","2022-05-18T13:07:09.000000Z"
9	"Musculoskeletal Pain"[mhe],"8","2022-05-18T13:06:36.000000Z"
8	"Pain"[mhe],"603","2022-05-18T13:05:27.000000Z"
7	(myofascial release) OR ("Myofascial Release Therapy"[mhe]) OR ((craniosacral OR cranio-sacral) AND (therap* OR treatment* OR manipulat*)) OR (osteopat*) OR ("Manipulation Osteopathic"[mhe]) OR ("Osteopathic Medicine"[mhe]),"33","2022-05-18T13:04:17.000000Z"
6	myofascial release,"0","2022-05-18T13:02:56.000000Z"
5	"Myofascial Release Therapy"[mhe],"0","2022-05-18T13:02:32.000000Z"
4	(craniosacral OR cranio-sacral) AND (therap* OR treatment* OR manipulat*),"29","2022-05-18T13:01:19.000000Z"
3	osteopat*,"4","2022-05-18T13:00:35.000000Z"
2	"Manipulation Osteopathic"[mhe],"2","2022-05-18T12:59:59.000000Z"
1	"Osteopathic Medicine"[mhe],"1","2022-05-18T12:59:26.000000Z"

Total hits:	9
Date of search:	18.05.2022

Search strategy for MEDLINE

Database: Ovid MEDLINE(R) and In-Process, In-Data-Review & Other Non-Indexed Citations and Daily <1946 to May 13, 2022>, Ovid MEDLINE(R) and Epub Ahead of Print, In-Process, In-Data-Review & Other Non-Indexed Citations and Daily <2018 to May 13, 2022>

Search Strategy:

----

1 exp Pain/ (507674)

2 exp Musculoskeletal Pain/ (9796)

3 exp Shoulder Pain/ (6866)

4 exp Headache Disorders/ (44912)

5 (pain* or ache* or aching or sore*).mp. (1199383)

6 headache*.mp. (133229)

7 neckache*.mp. (33)

8 backache*.mp. (4434)

9 1 or 2 or 3 or 4 or 5 or 6 or 7 or 8 (1374797)

10 exp Osteopathic Medicine/ (3644)

11 exp Manipulation, Osteopathic/ (1404)

12 osteopat*.mp. (9819)

13 ((craniosacral or cranio-sacral) adj (therap* or treatment* or manipulat*)).mp. (121)

14 exp Myofascial Release Therapy/ (28)

15 myofascial release.mp. (720)

16 10 or 11 or 12 or 13 or 14 or 15 (10515)

17 9 and 16 (1832)

18 limit 17 to randomized controlled trial (238)

19 ((randomized controlled trial or controlled clinical trial).pt. or randomized.ab. or placebo.ab. or drug therapy.fs. or randomly.ab. or trial.ab. or groups.ab.) not (exp animals/ not humans.sh.) (5905382)

20 17 and 19 (710)

21 limit 17 to (meta analysis or "systematic review") (112)

22 (((comprehensive* or integrative or systematic*) adj3 (bibliographic* or review* or literature)) or (meta-analy* or metaanaly* or "research synthesis" or ((information or data) adj3 synthesis) or (data adj2 extract*))).ti,ab. or (cinahl or (cochrane adj3 trial*) or embase or medline or psyclit or (psycinfo not "psycinfo database") or pubmed or scopus or "sociological abstracts" or "web of science").ab. or ("cochrane database of systematic reviews" or evidence report technology assessment or evidence report technology assessment summary).jn. or Evidence Report: Technology Assessment*.jn. or ((review adj5 (rationale or evidence)).ti,ab. and review.pt.) or meta-analysis as topic/ or Meta-Analysis.pt. (888917)

23 17 and 22 (222)

24 21 or 23 (224)

25 limit 24 to yr="2017 - 2022" (140)

26 18 or 20 or 25 (760)

27 limit 26 to (english or german) (739)

28 remove duplicates from 27 (505)

****

18.05.2022

Search strategy for PEDro

Datum der Suche: 19.05.2022

CT Suche (69 Hits)

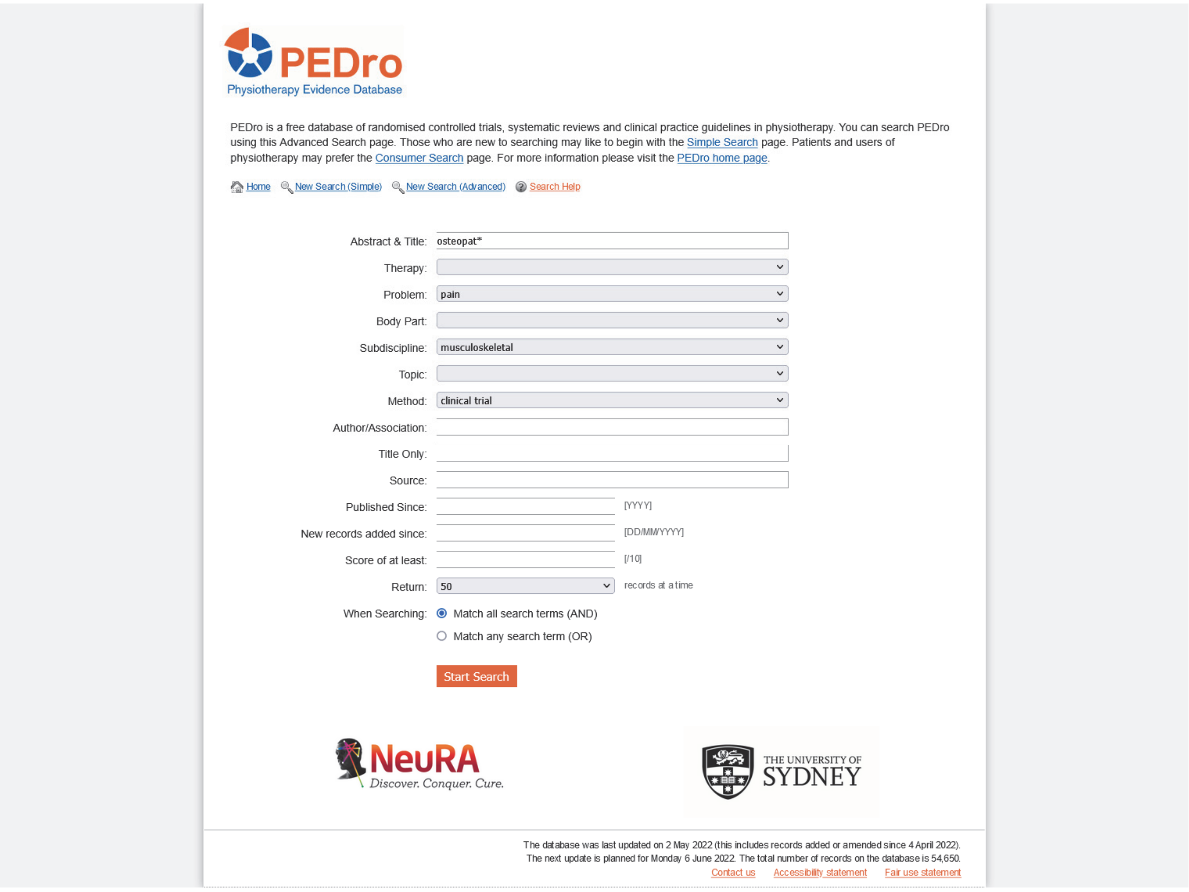

PEDro
Physiotherapy Evidence Database

PEDro is a free database of randomised controlled trials, systematic reviews and clinical practice guidelines in physiotherapy. You can search PEDro using this Advanced Search page. Those who are new to searching may like to begin with the Simple Search page. Patients and users of physiotherapy may prefer the Consumer Search page. For more information please visit the PEDro home page.

Home | New Search (Simple) | New Search (Advanced) | Search Help

Abstract & Title: osteopat*
Therapy:
Problem: pain
Body Part:
Subdiscipline: musculoskeletal
Topic:
Method: clinical trial
Author/Association:
Title Only:
Source:
Published Since: [YYYY]
New records added since: [DD/MM/YYYY]
Score of at least: [/10]
Return: 50 records at a time
When Searching: Match all search terms (AND) / Match any search term (OR)

Start Search

NeuRA Discover. Conquer. Cure.
THE UNIVERSITY OF SYDNEY

The database was last updated on 2 May 2022 (this includes records added or amended since 4 April 2022).
The next update is planned for Monday 6 June 2022. The total number of records on the database is 54,650.
Contact us | Accessibility statement | Fair use statement

SR Suche (16 Hits)

PEDro
Physiotherapy Evidence Database

PEDro is a free database of randomised controlled trials, systematic reviews and clinical practice guidelines in physiotherapy. You can search PEDro using this Advanced Search page. Those who are new to searching may like to begin with the Simple Search page. Patients and users of physiotherapy may prefer the Consumer Search page. For more information please visit the PEDro home page.

Home | New Search (Simple) | New Search (Advanced) | Search Help

Abstract & Title:	osteopat*
Therapy:	
Problem:	pain
Body Part:	
Subdiscipline:	musculoskeletal
Topic:	
Method:	systematic review
Author/Association:	
Title Only:	
Source:	
Published Since:	2017 [YYYY]
New records added since:	[DD/MM/YYYY]
Score of at least:	[/10]
Return:	20 records at a time
When Searching:	(•) Match all search terms (AND)
	( ) Match any search term (OR)

Start Search

NeuRA Discover. Conquer. Cure.

THE UNIVERSITY OF SYDNEY

The database was last updated on 2 May 2022 (this includes records added or amended since 4 April 2022). The next update is planned for Monday 6 June 2022. The total number of records on the database is 54,650.

Contact us | Accessibility statement | Fair use statement

Literature search strategies for the update search

Search strategy for Embase

Embase

Session Results

....

No. Query Results Results Date

#65. #64 AND [18-05-2022]/sd NOT [24-07-2025]/sd 289 23 Jul 2025

#64. #60 NOT #63 877 23 Jul 2025

#63. #61 OR #62 533,531 23 Jul 2025

#62. 'clinical trial':dtype 533,362 23 Jul 2025

#61. #60 AND 'Conference Abstract'/it 169 23 Jul 2025

#60. #59 AND ([english]/lim OR [german]/lim) 1,444 23 Jul 2025

#59. #17 OR #53 OR #58 1,471 23 Jul 2025

#58. #57 AND [2017-2022]/py 224 23 Jul 2025

#57. #54 OR #56 577 23 Jul 2025

#56. #16 AND #55 560 23 Jul 2025

#55. ('meta analysis'/exp OR 'systematic review'/exp 1,935,303 23 Jul 2025
OR ((meta NEAR/3 analy*):ab,ti) OR
metaanaly*:ab,ti OR review*:ti OR overview*:ti OR
((synthes* NEAR/3 (literature* OR research* OR
studies OR data)):ab,ti) OR (pooled AND
analys*:ab,ti) OR (((data NEAR/2 pool*):ab,ti)
AND studies:ab,ti) OR medline:ab,ti OR
medlars:ab,ti OR embase:ab,ti OR cinahl:ab,ti OR
scisearch:ab,ti OR psychinfo:ab,ti OR
psycinfo:ab,ti OR psychlit:ab,ti OR psyclit:ab,ti
OR cinhal:ab,ti OR cancerlit:ab,ti OR
cochrane:ab,ti OR bids:ab,ti OR pubmed:ab,ti OR
ovid:ab,ti OR (((hand OR manual OR database* OR
computer*) NEAR/2 search*):ab,ti) OR ((electronic
NEAR/2 (database* OR 'data base' OR 'data
bases')):ab,ti) OR bibliograph*:ab OR 'relevant

journals':ab OR (((review* OR overview*) NEAR/10 (systematic* OR methodologic* OR quantitativ* OR research* OR literature* OR studies OR trial* OR effective*)):ab)) NOT ((((retrospective* OR record* OR case* OR patient*) NEAR/2 review*):ab,ti) OR (((patient* OR review*) NEAR/2 chart*):ab,ti) OR rat:ab,ti OR rats:ab,ti OR mouse:ab,ti OR mice:ab,ti OR hamster:ab,ti OR hamsters:ab,ti OR animal:ab,ti OR animals:ab,ti OR dog:ab,ti OR dogs:ab,ti OR cat:ab,ti OR cats:ab,ti OR bovine:ab,ti OR sheep:ab,ti) NOT ('editorial'/exp OR 'erratum'/de OR 'letter'/exp) NOT (('animal'/exp OR 'nonhuman'/exp) NOT (('animal'/exp OR 'nonhuman'/exp) AND 'human'/exp))

#54. #16 AND ([cochrane review]/lim OR [systematic review]/lim OR [meta analysis]/lim) 309 23 Jul 2025

#53. #16 AND #52 1,275 23 Jul 2025

#52. #37 NOT #51 6,615,725 23 Jul 2025

#51. #38 OR #39 OR #40 OR #41 OR #42 OR #43 OR #44 OR #45 OR #46 OR #47 OR #48 OR #49 OR #50 4,908,309 23 Jul 2025

#50. 'animal experiment'/de NOT ('human experiment'/de OR 'human'/de) 2,764,638 23 Jul 2025

#49. (rat:ti,tt OR rats:ti,tt OR mouse:ti,tt OR mice:ti,tt OR swine:ti,tt OR porcine:ti,tt OR murine:ti,tt OR sheep:ti,tt OR lambs:ti,tt OR pigs:ti,tt OR piglets:ti,tt OR rabbit:ti,tt OR rabbits:ti,tt OR cat:ti,tt OR cats:ti,tt OR dog:ti,tt OR dogs:ti,tt OR cattle:ti,tt OR bovine:ti,tt OR monkey:ti,tt OR monkeys:ti,tt OR trout:ti,tt OR marmoset*:ti,tt) AND 'animal experiment'/de 1,310,165 23 Jul 2025

#48. (databases NEAR/5 searched):ab	83,200	23 Jul 2025
#47. 'update review':ab	154	23 Jul 2025
#46. 'we searched':ab AND (review:ti,tt OR review:it)	58,952	23 Jul 2025
#45. review:ab AND review:it NOT trial:ti,tt	1,307,637	23 Jul 2025
#44. ('random cluster' NEAR/4 sampl*):ti,ab,tt	1,791	23 Jul 2025
#43. 'random field*':ti,ab,tt	3,196	23 Jul 2025
#42. nonrandom*:ti,ab,tt NOT random*:ti,ab,tt	21,222	23 Jul 2025
#41. 'systematic review':ti,tt NOT (trial:ti,tt OR study:ti,tt)	332,941	23 Jul 2025
#40. 'case control*':ti,ab,tt AND random*:ti,ab,tt NOT ('randomised controlled':ti,ab,tt OR 'randomized controlled':ti,ab,tt)	24,266	23 Jul 2025
#39. 'cross-sectional study' NOT ('randomized controlled trial'/de OR 'controlled clinical study'/de OR 'controlled study'/de OR 'randomised controlled':ti,ab,tt OR 'randomized controlled':ti,ab,tt OR 'control group':ti,ab,tt OR 'control groups':ti,ab,tt)	481,775	23 Jul 2025
#38. ((random* NEXT/1 sampl* NEAR/8 ('cross section*' OR questionnaire* OR survey OR surveys OR database OR databases)):ti,ab,tt) NOT ('comparative study'/de OR 'controlled study'/de OR 'randomised controlled':ti,ab,tt OR 'randomized controlled':ti,ab,tt OR 'randomly assigned':ti,ab,tt)	3,745	23 Jul 2025
#37. #18 OR #19 OR #20 OR #21 OR #22 OR #23 OR #24 OR #25 OR #26 OR #27 OR #28 OR #29 OR #30 OR #31 OR #32 OR #33 OR #34 OR #35 OR #36	7,487,443	23 Jul 2025
#36. trial:ti,tt	560,140	23 Jul 2025
#35. 'human experiment'/de	722,866	23 Jul 2025
#34. volunteer:ti,ab,tt OR volunteers:ti,ab,tt	322,701	23 Jul 2025
#33. (controlled NEAR/8 (study OR design OR	638,448	23 Jul 2025

trial)):ti,ab,tt

#32. assigned:ti,ab,tt OR allocated:ti,ab,tt 601,163 23 Jul 2025

#31. ((assign* OR match OR matched OR allocation) NEAR/6 (alternate OR group OR groups OR intervention OR interventions OR patient OR patients OR subject OR subjects OR participant OR participants)):ti,ab,tt 556,463 23 Jul 2025

#30. crossover:ti,ab,tt OR 'cross over':ti,ab,tt 155,668 23 Jul 2025

#29. (parallel NEXT/1 group*):ti,ab,tt 52,510 23 Jul 2025

#28. 'double blind procedure'/de 306,428 23 Jul 2025

#27. ((double OR single OR doubly OR singly) NEXT/1 (blind OR blinded OR blindly)):ti,ab,tt 367,027 23 Jul 2025

#26. (open NEXT/1 label):ti,ab,tt 185,185 23 Jul 2025

#25. (evaluated:ab OR evaluate:ab OR evaluating:ab OR assessed:ab OR assess:ab) AND (compare:ab OR compared:ab OR comparing:ab OR comparison:ab) 3,291,794 23 Jul 2025

#24. compare:ti,tt OR compared:ti,tt OR comparison:ti,tt 708,596 23 Jul 2025

#23. placebo:ti,ab,tt 462,087 23 Jul 2025

#22. 'intermethod comparison'/de 315,420 23 Jul 2025

#21. 'randomization'/de 101,160 23 Jul 2025

#20. random*:ti,ab,tt 2,465,839 23 Jul 2025

#19. 'controlled clinical trial'/de 459,872 23 Jul 2025

#18. 'randomized controlled trial'/de 1,090,602 23 Jul 2025

#17. #16 AND [randomized controlled trial]/lim 737 23 Jul 2025

#16. #7 AND #15 3,765 23 Jul 2025

#15. #8 OR #9 OR #10 OR #11 OR #12 OR #13 OR #14 14,141 23 Jul 2025

#14. 'myofascial release' 1,389 23 Jul 2025

#13. 'myofascial release'/exp 858 23 Jul 2025

#12. (craniosacral OR 'cranio sacral') NEAR/1 (therap* OR treatment* OR manipulat*) 344 23 Jul 2025

#11. 'craniosacral therapy'/exp 273 23 Jul 2025

#10. osteopat*:ti,ab,lnk,kw,de 12,682 23 Jul 2025

#9. 'osteopathic manipulation'/exp 1,131 23 Jul 2025

#8. 'osteopathic medicine'/exp 6,699 23 Jul 2025

#7. #1 OR #2 OR #3 OR #4 OR #5 OR #6 2,550,228 23 Jul 2025

#6. backache* 80,614 23 Jul 2025

#5. neckache* 42 23 Jul 2025

#4. headache* 405,538 23 Jul 2025

#3. pain* OR ache* OR aching OR sore* 2,247,292 23 Jul 2025

#2. 'headache and facial pain'/exp 436,606 23 Jul 2025

#1. 'musculoskeletal pain'/exp 223,985 23 Jul 2025

....

Search strategy for Cochrane

Search Name: Osteopathy for musculoskeletal pain (Update 2025)

Last Saved: 23/07/2025 15:58:46

Comment: LG/VH 180522

ID	Search
#1	MeSH descriptor: [Pain] explode all trees
#2	MeSH descriptor: [Musculoskeletal Pain] explode all trees
#3	MeSH descriptor: [Shoulder Pain] explode all trees
#4	MeSH descriptor: [Headache Disorders] explode all trees
#5	((pain* OR ache* OR aching OR sore*)) (Word variations have been searched)
#6	(headache*) (Word variations have been searched)
#7	(neckache*) (Word variations have been searched)
#8	(backache*) (Word variations have been searched)
#9	#1 OR #2 OR #3 OR #4 OR #5 OR #6 OR #7 OR #8
#10	MeSH descriptor: [Osteopathic Medicine] explode all trees
#11	MeSH descriptor: [Manipulation, Osteopathic] explode all trees
#12	(osteopat*) (Word variations have been searched)
#13	((craniosacral OR cranio-sacral) NEAR (therap* OR treatment* OR manipulat*)) (Word variations have been searched)

#14 MeSH descriptor: [Myofascial Release Therapy] explode all trees

#15 ("myofascial release") (Word variations have been searched)

#16 #10 OR #11 OR #12 OR #13 OR #14 OR #15

#17 #9 AND #16

#18 #9 AND #16 in Trials

#19 #9 AND #16 with Cochrane Library publication date Between Jan 2017 and May 2022, in Cochrane Reviews, Cochrane Protocols

#20 #18 OR #19

#21 (conference abstract):pt

#22 (abstract):so

#23 (clinicaltrials OR trialsearch OR ANZCTR OR ensaiosclinicos OR Actrn OR chictr OR cris OR ctri OR registroclinico OR clinicaltrialsregister OR DRKS OR IRCT OR Isrctn OR rctportal OR JapicCTI OR JMACCT OR jRCT OR JPRN OR Nct OR UMIN OR trialregister OR PACTR OR R.B.R.OR REPEC OR SLCTR OR Tcr):so (Word variations have been searched)

#24 #21 OR #22 OR #23

#25 #20 NOT #24

#26 #20 NOT #24 with Cochrane Library publication date Between May 2022 and Jul 2025

245 Hits

Search strategy for INAHTA

Search step #	Search query,"Hits","Searched At"
	((((backache*) OR (neckache*) OR (headache*) OR (pain* OR ache* OR aching OR sore*) OR ("Headache Disorders"[mhe]) OR ("Shoulder Pain"[mhe]) OR ("Musculoskeletal Pain"[mhe]) OR ("Pain"[mhe])) AND ((myofascial release) OR ("Myofascial Release Therapy"[mhe]) OR ((craniosacral OR cranio-sacral) AND (therap* OR treatment* OR manipulat*)) OR (osteopat*) OR ("Manipulation Osteopathic"[mhe]) OR ("Osteopathic Medicine"[mhe]))) AND (English OR German)[Language]) FROM 2022 TO 2025,"1","2025-07-23T14:55:21.000000Z"
	(((backache*) OR (neckache*) OR (headache*) OR (pain* OR ache* OR aching OR sore*) OR ("Headache Disorders"[mhe]) OR ("Shoulder Pain"[mhe]) OR ("Musculoskeletal Pain"[mhe]) OR ("Pain"[mhe])) AND ((myofascial release) OR ("Myofascial Release Therapy"[mhe]) OR ((craniosacral OR cranio-sacral) AND (therap* OR treatment* OR manipulat*)) OR (osteopat*) OR ("Manipulation Osteopathic"[mhe]) OR ("Osteopathic Medicine"[mhe]))) AND (English OR German)[Language],"3","2025-07-23T14:55:02.000000Z"
Total hits	1
Date of search	23.07.2025

Search strategy for MEDLINE

Database: Ovid MEDLINE(R) ALL <1946 to July 22, 2025>

Search Strategy:

----

1 exp Pain/ (491624)

2 exp Musculoskeletal Pain/ (8575)

3 exp Shoulder Pain/ (6491)

4 exp Headache Disorders/ (43079)

5 (pain* or ache* or aching or sore*).mp. (1119283)

6 headache*.mp. (124571)

7 neckache*.mp. (26)

8 backache*.mp. (4113)

9 1 or 2 or 3 or 4 or 5 or 6 or 7 or 8 (1285731)

10 exp Osteopathic Medicine/ (3473)

11 exp Manipulation, Osteopathic/ (1303)

12 osteopat*.mp. (9322)

13 ((craniosacral or cranio-sacral) adj (therap* or treatment* or manipulat*)).mp. (118)

14 exp Myofascial Release Therapy/ (46)

15 myofascial release.mp. (706)

16 10 or 11 or 12 or 13 or 14 or 15 (10005)

17 9 and 16 (1734)

18 limit 17 to randomized controlled trial (239)

19 ((randomized controlled trial or controlled clinical trial).pt. or randomized.ab. or placebo.ab. or drug therapy.fs. or randomly.ab. or trial.ab. or groups.ab.) not (exp animals/ not humans.sh.) (5575486)

20 17 and 19 (685)

21 limit 17 to (meta analysis or "systematic review") (116)

22 (((comprehensive* or integrative or systematic*) adj3 (bibliographic* or review* or literature)) or (meta-analy* or metaanaly* or "research synthesis" or ((information or data) adj3 synthesis) or (data adj2 extract*))).ti,ab. or (cinahl or (cochrane adj3 trial*) or embase or medline or psyclit or (psycinfo not "psycinfo database") or pubmed or scopus or "sociological abstracts" or "web of science").ab. or ("cochrane database of systematic reviews" or evidence report technology assessment or evidence report technology assessment summary).jn. or Evidence Report: Technology

Assessment*.jn. or ((review adj5 (rationale or evidence)).ti,ab. and review.pt.) or meta-analysis as topic/ or Meta-Analysis.pt. (892774)

23 17 and 22 (236)

24 21 or 23 (237)

25 limit 24 to yr="2017 - 2022" (86)

26 18 or 20 or 25 (716)

27 limit 26 to (english or german) (701)

28 remove duplicates from 27 (700)

29 (CN-01752910 or "32010000843" or "32011000900" or "32014000953" or "32014001216" or CN-02111368 or CN-01368141 or CN-01395887 or CN-02126903 or "35481320" or "20201159" or "21943616" or "25603749" or "24703512" or "22236639" or "24725797" or "34552791" or "31563370" or "29884165" or "28892807" or "32001000127" or "31453500" or "21609913" or CD012622 or "34790245" or "16942472" or "10547405" or "33446440" or CD012987 or "31980366" or CD011388 or "30784788" or "28441294" or "30168356" or "21135862" or "34444389" or "23337558" or "1402408" or "23981319" or "33694338" or "23843760" or CN-00707228 or "35082352" or "35414546" or "31790129" or "24327820" or "19804629" or CN-00209283 or "34999574" or "23294689" or "32598457" or "17986674" or "18528681" or "32831879" or "33777508" or "25694375" or "15953306" or "32056816" or CN-00580103 or "8432669" or "32026838" or CN-02112486 or CN-01339261 or "27235304" or "34440589" or "23697915" or "15089958" or CN-00724786 or CN-00729730 or "11562654" or "34092587" or CD012367 or CN-00174963 or "10905437" or "34364317" or "32554836" or "31384337" or CN-00630418 or CN-00399726 or "32930638" or "26059857" or CN-00613467 or CN-02112088 or "12884943" or "31126577" or "29681188" or CN-02112098 or "33991702" or "27347698" or "21673013" or "21234327" or "20702514" or "30691766" or "12361180" or "18154193" or "28139112" or CN-00892749 or "34991428" or "32081945" or "25872943" or "32615581" or "25847552" or "28352200" or "34768531" or "19767483" or "29037655" or CN-02112294 or "30100300" or "31563369" or "26642754" or "33984499" or CN-00395377 or "35233332" or CN-02082476 or "33935056" or "34719122" or "9348143" or "19712459" or CN-00641875 or "21135197" or "24605910" or "33856751" or "33517104" or "33948127" or "33596925" or "16182028" or CN-00707946 or "23372389" or "20226365" or "30050749" or "34011108" or "28241328" or "21732734" or CN-02146642 or "35389057" or "33197571" or "16368905" or CN-02306576 or CN-02303892 or "33789971" or "33276229" or CN-02308809 or "11982831" or "31658037" or "31987543" or "6809281" or "17908831" or "17908827" or CN-00579324 or "28456600" or "31404469" or "8846540" or "35248270" or CD004407 or "34522466" or "26945216" or "31847158" or CN-02112087 or "30691765" or "27210859" or CN-01656546 or "34517916" or "16944082" or CN-00474533 or "29454150" or 29780605; 31635110 or "14527076" or "23369067" or "32827553" or "10073623" or CN-00779737 or "30572544" or CN-01248602 or "26928164" or "29708766" or "33831981" or "20308941" or "16033646" or CN-02107812 or "16826363" or "29499728" or "22018755" or "16052120" or "19539119" or "29238246" or "33714517" or "1399600" or "35275574" or "33064421" or "20659716" or "23036874" or "29037623" or "25175885" or "23833154" or "33635954" or "33818033" or "28750310" or CN-00036451 or CN-00699035 or CD013042 or CN-00580104 or "17371579" or "28731832" or "29135458" or "16249365" or "12090649" or "30202408" or "25284739" or "15138373" or CN-00789667 or CN-02147644 or "29266633" or "30425565" or CN-00577902 or "28029069" or "32318454" or "32426194" or "21970918" or CD012720 or "2860453" or CN-02334429 or "16118354" or "29502293" or CN-

00700258 or "29582181" or "35404394" or CN-01171480 or "26118527" or "32507144" or "28306531" or "30787664" or "6218150" or CN-00789569 or "33909266" or "27661020" or "19425461" or "26340656" or "31892357" or "25453528" or "18806078" or "10436401" or "33694345" or "21214395" or "12635637" or "27455099" or CD015074 or "32017000072" or "32017000393" or "32018000066" or "23588488" or CN-00090108 or CN-00983534 or "25068560" or "27802557" or "31566694" or "27802555" or "30799839" or "28027445" or "21943613" or "30276016" or CN-00399327 or CN-01986911 or "12370877" or "17079523" or CN-01779511 or CN-01779512 or "18387190" or CN-00325106 or "23009547" or CN-00962119 or "25029347" or "21641520" or CN-00782686 or "35331719" or CD010842 or "27166404" or "31790128" or CN-00754154 or CN-00680967 or "33475581" or CN-01383532 or "22415216" or "29262824" or "22182954" or "33992303" or CN-00168245 or CN-00987596 or "11392208" or CN-02110824 or CN-00632718 or "19874246" or "34945240" or "21147420" or CN-00452682 or "28532889" or "31103113" or "34185226" or "28801412" or "33888163" or "35452024" or "34092603" or "22027034" or "21486710" or CN-01748582 or "23374199" or "33005994" or "12138953" or "33728108" or "33749637" or "21696647" or "27367949" or CN-00781234 or CN-02308837 or "33232012" or "28956477" or CN-00783337 or "34662041" or "33919315" or "29570571" or "30860417" or "33947735" or "20801246" or "17324291" or CN-00762722; CN-02007476 or "23562352" or "30935517" or CN-02273519 or CN-00348090 or "17371583" or "19122835" or "34852185" or "24084800" or "24965494" or "16080794" or "19766977" or "31985765" or "26927909" or "26927908" or "23212429" or "22802542" or "24704126" or "22984233" or "23739758" or "23759340" or "18439282" or "23508598" or "16943515" or "32765058" or "15176518" or "12838090" or "26422811" or "23768283" or "34331755" or "33705610" or "18824395" or "34391228" or "30327397" or "30691751" or CN-00329230 or CN-00082520 or "18211590" or "2141951" or "27651843" or "34344606" or "25920314" or CN-00892528 or CN-00689162 or "18541041" or CN-00510509 or CN-01723372 or CN-00704958 or CN-01785471 or CN-01768541 or "29298077" or "29782181" or CN-00567780 or "26165865" or "29787734" or "19705093" or "23354911" or CN-02115334 or "19729492" or "32598462" or CN-02309161 or "28532881" or CN-02122342 or "15784928" or "23036875" or CD014699 or "32514278" or "32005000248" or "29097951" or "16762670" or "20226363" or "32734929" or "34955570" or CN-02136830 or CD013423 or "32673652" or "18380529" or "33512387" or "28858681" or "23463485" or "27012575" or "35176817" or "31790124" or "32826011" or "24917634" or "35160211" or "33989990" or CN-00633341 or "22384933" or "11130229" or "33720272" or "18154191" or "19814829" or "12372311" or "34660797" or CD013018 or CN-00760045 or "8017259" or "23570655" or "33523036" or CN-00775967 or "34575151" or "31631992" or "24618345" or CD005015 or "23289034" or "22582197" or "30866915" or "28532888" or "32537276" or "29181524" or "12196279" or "34067152" or "23904227" or "32796456" or CN-00740377 or CN-00616092 or "24439098" or "25357218" or "16914363" or "22078065" or "8064652" or "17161532" or "31864435" or "2793535" or CN-01074972 or "29175715" or CD013041 or "19011230" or "29809257" or "15040418" or "21053038" or "28952872" or "34166796" or "9133925" or "33694350" or "24481801" or "27814849" or "28508278" or CN-02368280 or CN-02134996 or "28109570" or "26205357" or "9615558" or "22863643" or "34776157" or "35044712" or "16118359" or "17908829" or "31050562" or CN-00691581 or "32946545" or "33721921" or "23918915" or CN-00778410 or CN-00633805 or CN-00633808 or "4253659" or "34484377" or CN-02114859 or "28646809" or "24410925" or "30973196" or "33567080" or "33128074" or "30261029" or "26745225" or "32928567" or "28678033" or "32317109" or CN-02389618 or "25170037" or "29043110" or "27884147" or "32825989" or CN-00616087 or "33654501" or "6848057" or "34515079" or 33354233; 16211338 or CN-00349103 or "22972127" or "35038641" or CN-00620890 or CN-02297769 or "23322328" or "34266436" or CN-00754018 or "33946640" or "34753085" or CN-02136126 or "24678115" or CN-01343842 or "19706829" or "1310461" or "23315555" or

"34620110" or "35258631" or "32967389" or "30640585" or "6424236" or "18617745" or "32825990" or "23273259" or "26111129" or "30398571" or "31322903" or "26500355" or "33218555" or "30470892" or "28532891" or "16627780" or CN-02387442 or CN-02200974 or "30534176" or "22628581" or "34844288" or CD013589 or "32338846" or "30179389" or CD009514 or "29589380" or "19445800" or "21663705" or "24005089" or "21357494" or "18723455" or "29480916" or "22904246" or "21220082" or CN-02107690 or CN-00725119 or CN-01707268 or "11317015" or "33431282" or CN-00390066 or CN-00443815 or CN-01712162 or "29742744" or "33241157" or "9439253" or "29234493" or "24917632" or "12889861" or "35076554" or CD012650 or "35280247" or "35248268" or "35206825" or "27858681" or "19836601" or "27455103" or "33423376" or "821993" or "31741344" or "31462989" or CN-01108740 or "18586346" or "8932590" or CN-01783349 or "21943614" or "22703751" or "33214374" or "32091560" or CN-02134884 or "24986566" or "34507243" or "34948580" or "33184777" or "30475751" or "16880877" or "3061913" or CN-00758213 or "15632984" or "30388155" or "25792906" or "31372099" or "32962529" or "22658268" or "3934322" or CN-00641217 or CD011618 or "21385086" or "7512066" or "24614605" or CN-00164202 or CN-00906248 or "33354391" or "31103106" or CN-00088830 or "10453576" or "16181182" or "32873418" or "32900544" or "15456045" or "10769433" or "17544863" or CN-00621970 or "33870003" or "17565394" or "30765920" or "34079600" or "9474832" or "15531626" or "14701889" or "30860410" or "3043956" or "24725782" or "24205786" or "34395477" or "21349889" or "34592023" or CN-01306672 or CN-02144427 or "26304789" or "31136541" or "35488711" or "30479519" or "27821941" or "25457196" or "34462283" or "33333491" or "24117063" or "22891634" or "34916778" or "35228957" or "8629827").ui. (498)

30 28 not 29 (203)

****

23.07.2025

Search strategy for PEDro

Datum der Suche: 23.07.2025

CT Suche (9 Hits)

PEDro is a free database of randomised controlled trials, systematic reviews and clinical practice guidelines in physiotherapy. You can search PEDro using this Advanced Search page. Those who are new to searching may like to begin with the Simple Search page. Patients and users of physiotherapy may prefer the Consumer Search page. For more information please visit the PEDro home page.

Home   New search (Simple)   New Search (Advanced)   Search Help

Abstract & Title:	osteopat*	
Therapy:		
Problem:	pain	
Body Part:		
Subdiscipline:	musculoskeletal	
Topic:		
Method:	clinical trial	
Author/Association:		
Title Only:		
Source:		
Published Since:		[YYYY]
New records added since:	19/05/2022	[DD/MM/YYYY]
Score of at least:		[/10]
Return:	50	records at a time
When Searching:	(•) Match all search terms (AND)	
	( ) Match any search term (OR)	

Start Search

SR Suche (8 Hits)

PEDro is a free database of randomised controlled trials, systematic reviews and clinical practice guidelines in physiotherapy. You can search PEDro using this Advanced Search page. Those who are new to searching may like to begin with the Simple Search page. Patients and users of physiotherapy may prefer the Consumer Search page. For more information please visit the PEDro home page.

Home | New search (Simple) | New Search (Advanced) | Search Help

Abstract & Title:	osteopat*	
Therapy:		
Problem:	pain	
Body Part:		
Subdiscipline:	musculoskeletal	
Topic:		
Method:	systematic review	
Author/Association:		
Title Only:		
Source:		
Published Since:		[YYYY]
New records added since:	19/05/2022	[DD/MM/YYYY]
Score of at least:		[/10]
Return:	50	records at a time
When Searching:	◉ Match all search terms (AND)	
	○ Match any search term (OR)	

Start Search
